# The critical role of GRP78/BiP MARylation in ER stress of KRAS-mutant colorectal cancer

**DOI:** 10.1172/jci.insight.182809

**Published:** 2026-01-23

**Authors:** Shuxian Zhang, Xiaodan Chen, Qian Gong, Jing Huang, Yi Tang, Ming Xiao, Ming Li, Qingshu Li, Yalan Wang

**Affiliations:** 1 Molecular Medicine and Cancer Research Center, Basic Medicine College,; 2 Molecular Medicine Diagnostic and Testing Center, and; 3 Department of Pathology, The First Affiliated Hospital of Chongqing Medical University, Chongqing, China.

**Keywords:** Cell biology, Gastroenterology, Oncology, Cell stress, Colorectal cancer

## Abstract

Nearly 50% of patients with KRAS-mutant colorectal cancer (CRC) currently lack effective targeted therapy. The accumulation of KRAS-mutant proteins can trigger a sustained high level of endoplasmic reticulum (ER) stress, and the UPR-based long-term protective regulatory pathway inhibits the aggregation of unfolded proteins, thereby maintaining the stability of the ER and enabling the continued survival of KRAS-mutant tumors. However, the critical factors that affect the regulation of ER homeostasis in KRAS-mutant CRC are still unclear. Mono-ADP ribosylation (MARylation) catalyzed by ART1 is the most important modification of GRP78/BiP and stabilizes the internal environment of the ER. In this study, KRAS mutation increased the levels of ART1, ER stress, and MARylated GRP78/BiP in CRC cells. Inhibiting MARylated GRP78/BiP can impede the downstream IRE1α/XBP1/TFAF2/JNK and PERK/eIF2α/ATF4 cascades by affecting the binding and dissociation of GRP78/BiP with receptors to hinder the growth of KRAS-mutant CRC cells and accelerate their apoptosis. We propose that KRAS-mutant CRC cells are more sensitive to intervention with MARylated GRP78/BiP because more modifications are needed to maintain ER stability. We also conducted a preliminary study on the specific site of function. Clarifying this molecular mechanism can provide a experimental basis for identifying effective targets for the intervention of KRAS-mutant CRC.

## Abstract 2

mono-ADP-ribosylated GRP78/BiP affects the ER stress-related signaling pathway to regulate endoplasmic reticulum homeostasis and plays a vital role in the survival of KRAS mutant colorectal cancer cells.

## Graphical abstract

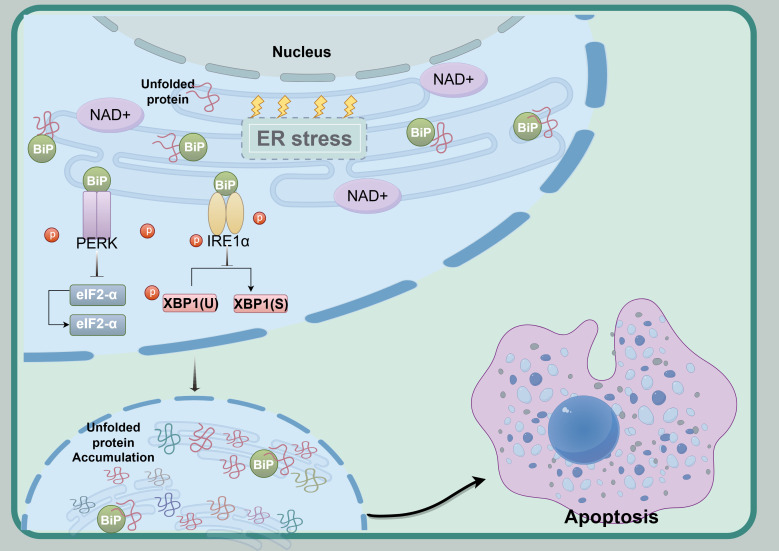

## Introduction

Colorectal cancer (CRC) remains one of the most common and deadly malignancies. Despite recent advances in early screening and precise molecular targeted therapy, the overall survival rate of patients with advanced CRC is still unsatisfactory, especially for those with metastatic CRC, which is the leading cause of high mortality (1). Approximately 45% of CRC cases involve oncogenic KRAS mutations, and mutation rates are higher in patients with multiple liver metastases (34% vs. 18%, *P* = 0.02) and multiple liver metastases within 1 year (36% vs. 14%, *P* = 0.005) (2). Oncogenic KRAS, which drives the activation of multiple signaling pathways (PI3K/PDK1/AKT and RAF/MEK1/2/ERK1/2), broadly promoting cell survival and proliferation, has become a bottleneck in the clinical treatment of CRC (3–5). The 2021 National Comprehensive Cancer Network (NCCN) practice guidelines for CRC noted that EGFR inhibitors (cetuximab and panitumumab) provide clear clinical benefits in the treatment of patients with RAS-WT metastatic CRC but cannot benefit patients with KRAS or NRAS mutations (6). Although promising, other targeted inhibitors (direct downregulation of KRAS or blockade of downstream signaling pathways) are challenging and difficult (7–9). Successfully targeting KRAS will require not only strategies to focus on targeting KRAS directly but also the critical identification of novel therapeutic targets.

A range of stressful cellular conditions accompanied by tumor growth may disrupt endoplasmic reticulum (ER) homeostasis and trigger ER stress (ERS) by activating the unfolded protein response (UPR) to maintain the normal function of tumor cells or induce apoptosis when severe ERS is sustained (10). In recent years, researchers have reported that ERS-related biomolecules seem to have therapeutic potential in CRC because of their bidirectional regulatory mechanism (8, 11). However, oncogenic RAS also causes ERS; once triggered, the UPR is engaged as a protective mechanism characterized by increased expression of GRP78 (78 kDa glucose-regulated protein 78, also known as BiP) and other chaperones to reduce protein accumulation caused by KRAS mutations (12, 13). However, the ERS state and its regulatory mechanism in KRAS-mutant CRC are still unclear.

The mono-ADP-ribosylation (MARylation) reaction involves the transfer of a single ADP-ribose residue from nicotinamide adenine dinucleotide (NAD^+^) to a specific amino acid of the substrate protein, and the release of nicotinamide is a reversible posttranslational modification. Known modifying enzymes involved in MARylation reactions have been classified as ADP-ribosyltransferases, diphtheria toxin–like (ARTDs) and ARTCs (clostridia toxin–like), and ARTC1 (ART1) has been characterized as an arginine-specific enzyme with a wide range of biological functions. Our research group also reported that the expression of ART1 in CRC tissues was significantly greater than that in paracancerous tissues and was correlated with the growth and metastasis of CRC (14). Increased expression of ART1 was detected in KRAS-mutated CRC cells (CT26 and LoVo cells) (15), and in KRAS-mutated CT26 CRC cells, ART1 inhibited the apoptosis induced by CDDP (16). Arginine MARylation may be related to the progression of CRC with KRAS mutations, but this relationship has not yet been studied in depth. At present, GRP78/BiP, integrin α7, neutrophil defensin 1 (HNP-1), fibroblast growth factor 2 (FGF2), and platelet-derived growth factor-BB (PDGF-BB) have recently been confirmed as substrates of ART1 (17, 18). GRP78/BiP is an ER chaperone that plays a key role in protein folding and quality control. Researchers have provided evidence that GRP78/BiP undergoes ADP ribosylation and that ADP ribosylation, as a rapid posttranslational mechanism for reversible inactivation of GRP78/BiP through interference with the allosteric coupling of the 2 domains (Arg470 and Arg492) of GRP78/BiP, may play an important role in the rapid regulation of the unfolded protein load (19, 20). More direct evidence has shown that MARylated GRP78/BiP provides a buffering system that balances protein processing rates with those of protein synthesis (21). However, the deep molecular mechanism of this modification is still unclear, and whether increased MARylated GRP78/BiP can affect the release of GRP78/BiP interactors and subsequently determine the downstream signaling pathways that can participate in it remains unclear. Therefore, the arginine MARylation of GRP78 catalyzed by ART1 may be a key link in the regulation of ERS in KRAS-mutant CRC and may be an important factor that promotes the progression of KRAS-mutant CRC.

## Results

### KRAS mutation significantly contributes to poor patient prognosis in CRC.

**Figure 1:**
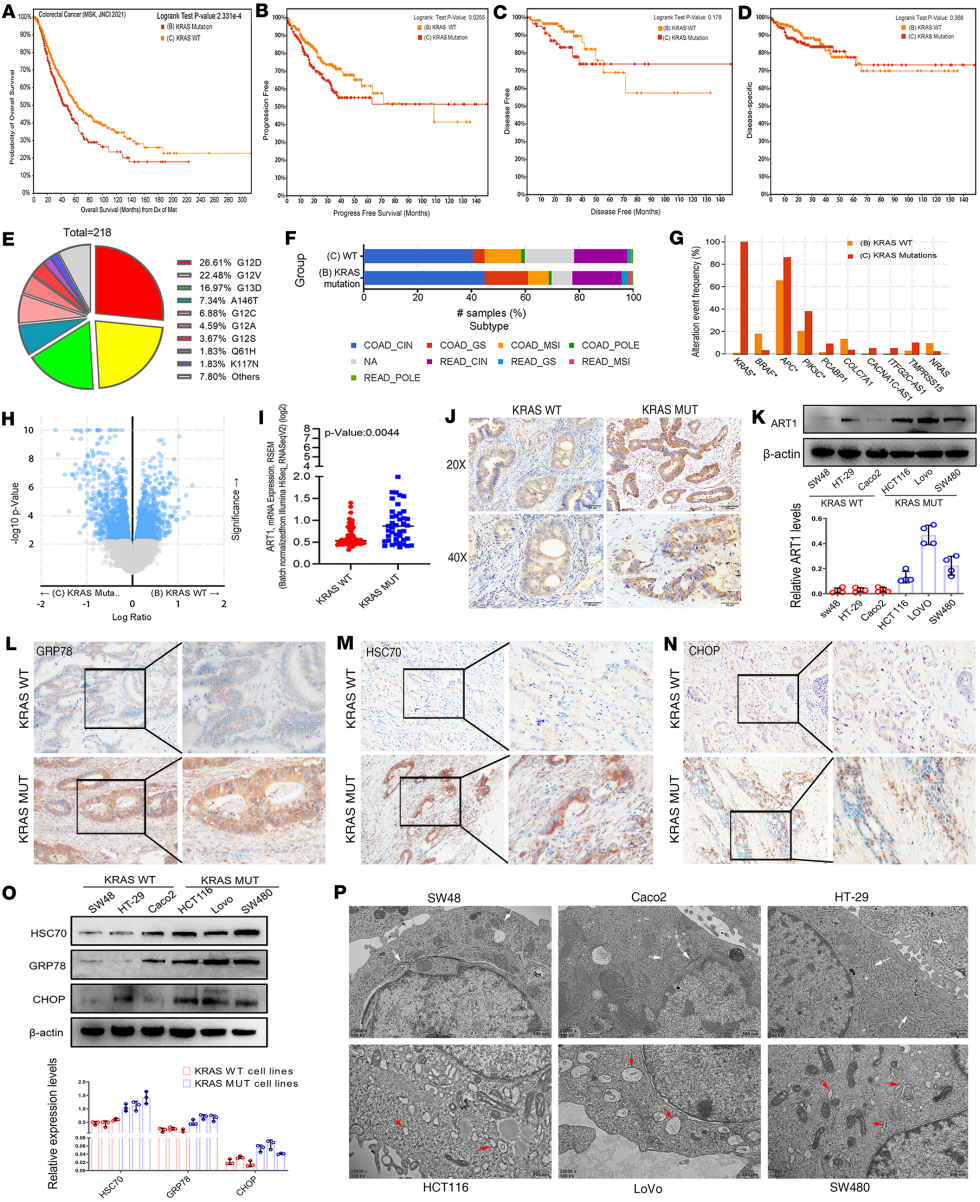
Expression and correlation of ART1 and ER stress markers in KRAS-mutant CRC. The analysis of overall survival (**A**), progression-free survival (**B**), disease-free survival (**C**), and disease-specific survival (**D**) analysis of patients with KRAS-WT and -mutant CRC based on the Cbioportal database. (**E**) The proportion of each mutation subtype in KRAS-mutant CRC. (**F**) The proportion of patients with KRAS-WT and -mutant patients in each subtype of CRC. (**G**) The mutation event frequency of various genes in KRAS-WT and -mutant CRC. (**H**) Differentially expressed genes in patients with KRAS-WT and -mutant CRC. (**I**) ART1 mRNA levels in KRAS-WT and -mutant CRC tissues. *P* < 0.05 by *t* test. (**J**) The positive intensity of ART1 in KRAS-WT and -mutant CRC tissues detected by immunohistochemistry. Original magnification, ×20 (top) and ×40 (bottom). (**K**) The expression of ART1 protein in KRAS-WT (SW48, Caco2, HT-29) and -mutant CRC cell lines (LoVo, HCT-116, SW480) observed by Western blot. Immunohistochemical detection of the expression of GRP78/BiP (**L**), HSC70 (**M**), and CHOP (**N**) in KRAS-WT and -mutant CRC tissues. Original magnification, ×200 (left) and ×400 (right). (**O**) The expression of GRP78/BiP, HSC70, and CHOP proteins in KRAS-WT and -mutant CRC cell lines detected by Western blot. (**P**) Transmission electron microscopy shows the morphology of the ER (arrows) in KRAS-WT and -mutant CRC cell lines. Original magnification, ×25,000.

First, we analyzed the types of KRAS mutations in CRC and their impact on overall survival (OS) (Figure 1A), progression-free survival (PFS) (Figure 1B), disease-free survival (DFS) (Figure 1C), and disease-specific survival (DSS) (Figure 1D) of patients with CRC. Our results revealed that patients with KRAS mutations had significantly poorer OS than did those with WT KRAS (*P* = 2.331 × 10^–4^), and the 10-year PFS of patients with KRAS-mutant CRC was significantly shorter than that of patients with KRAS-WT CRC (*P* = 0.0255). Statistics on the percentages of various types of KRAS mutations in patients with CRC indicated that the most common KRAS mutations occurred in exons 12 and 13, and G12D, G12V, G13D, A146T, and G12C were the 5 most common mutation types (Figure 1E). The proportions of patients with WT and mutant KRAS in each subtype of CRC are shown in Figure 1F. As shown in Figure 1G, the frequency of mutations in various genes differed between the 2 groups, namely, the KRAS-WT and -mutant groups. Among them, the frequency of BRAF mutations was lower in the KRAS-mutated group and higher in the KRAS-WT group, whereas APC and PIK3CA mutations showed the opposite trend.

### ART1 expression and ERS levels are greater in KRAS-mutant CRC than in KRAS-WT CRC.

A total of 2193 differentially expressed genes were extracted from the analysis of transcriptome sequencing data from 316 patients with WT KRAS and 218 patients with KRAS-mutant CRC from The Cancer Genome Atlas (TCGA) database; compared with those in the KRAS-WT CRC group, 1063 genes were highly expressed in KRAS-mutant CRC tissue, and 1130 genes were expressed at low levels in the KRAS-mutant CRC group (Figure 1H). The expression of ART1 at the mRNA level in the KRAS mutants was greater than that in the WT group (*P* = 0.0044) (Figure 1I). The results of ART1 immunohistochemical staining in 79 cases of CRC (58 cases were KRAS-mutant CRC, and 21 cases were KRAS-WT CRC) revealed that ART1 was expressed in the cytoplasm in a fine granular form (Figure 1J), and in KRAS-mutant tissues, the positive intensity of ART1 was generally greater than that in KRAS-WT tissues (*P* < 0.01). The results of the ART1-positive-level assessment are shown in Table 1. The level of ART1 was significantly correlated with the KRAS mutation status according to the χ^2^ test (*P* < 0.0001). Western blot analysis revealed that the expression of the ART1 protein in KRAS-mutant CRC cell lines (LoVo, HCT-116, and SW480) was greater than that in KRAS-WT cell lines (SW48, Caco2, and HT-29). More importantly, the expression level of ART1 was the highest in LoVo cells harboring 2 KRAS mutation sites (Figure 1K).

We subsequently explored the changes in the level of ERS in patients with KRAS mutations. First, the positive intensity of GRP78/BiP, HSC70, and CHOP in KRAS-mutant tissues was generally greater than that in KRAS-WT CRC tissues (Figure 1, L–N), and the degree of positive staining was significantly correlated with the KRAS mutation status, as determined through immunohistochemical staining (Table 2, Table 3, Table 4). In particular, GRP78/BiP-positive particles were stably expressed in the cytoplasm of tumor cells; however, the nuclei were strongly positive in 9 cases of mutant CRC tissues (Figure 1L). CHOP was expressed in the cytoplasm, and in 49 of the 51 cases of KRAS-mutant CRC, it was positively expressed in the nucleus; only 1 of the 28 cases of KRAS-WT CRC had positive nuclear expression (Figure 1N). Similarly, Western blot analysis revealed that the expression levels of 3 ERS markers were greater in LoVo, HCT-116, and SW480 cells than in SW48, Caco2, and HT-29 cells (Figure 1O). Transmission electron microscopy revealed that the rough ER in LoVo, HCT-116, and SW480 cells was round and swollen, whereas the rough ER in SW48, Caco2, and HT-29 cells was flat, cystic, and neatly arranged, and the ER cavity was narrow (Figure 1P). These findings suggest that the KRAS mutation promotes an increase in the level of ERS. Correlation analysis of the protein expression levels of ART1 and GRP78/BIP based on the immunohistochemical results of 51 cases of KRAS-mutated CRC revealed a positive correlation between ART1 and GRP78/BIP, with a correlation coefficient of 0.549 (*P* < 0.05) (Table 5).

### ART1 affects the level of arginine-MARylated GRP78/BiP in KRAS-mutant CRC cells.

**Figure 2:**
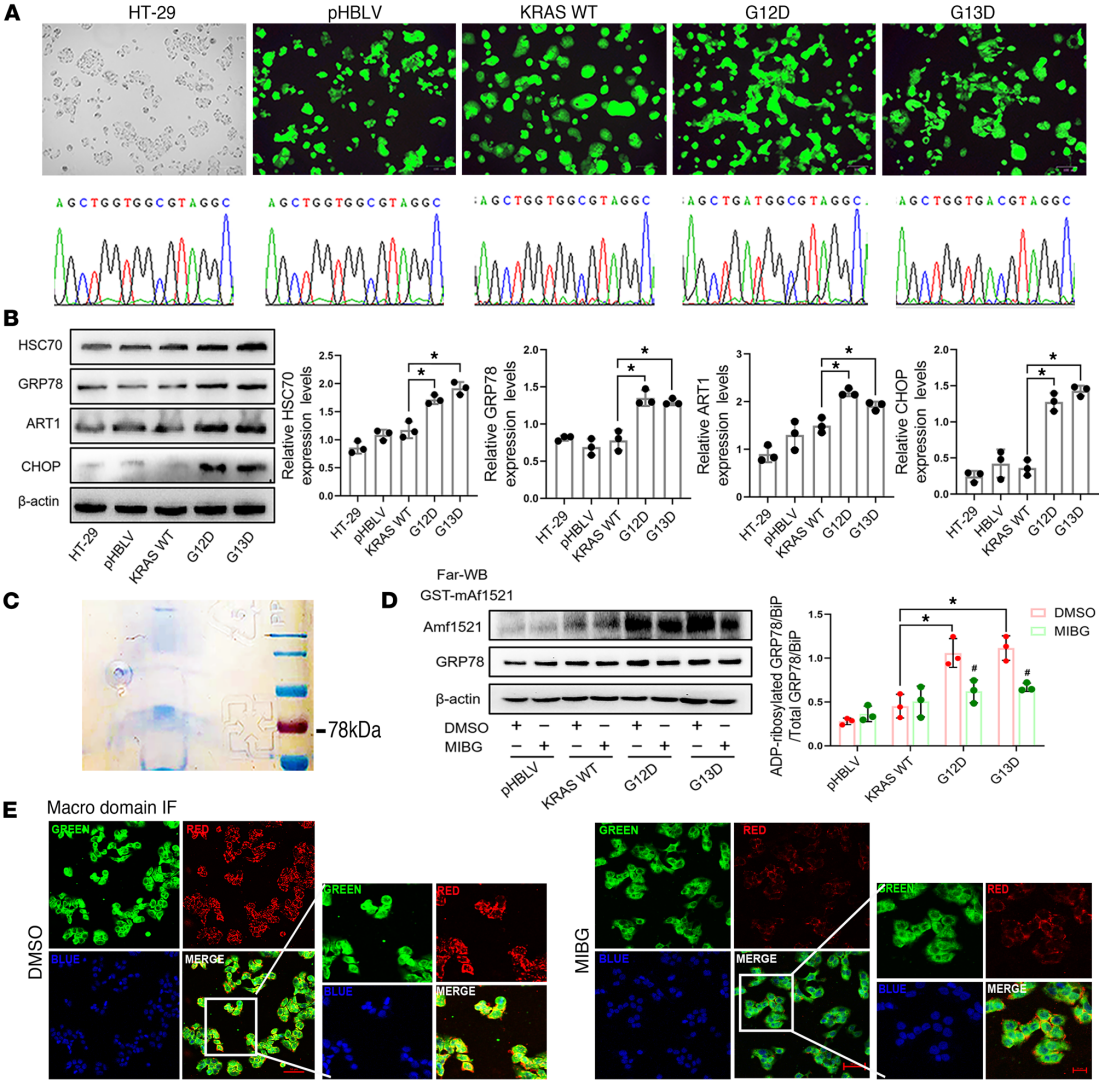
Effect of ART1 on the level of arginine–mono-ADP-ribosylated GRP78/BiP in KRAS-mutant CRC cells. (**A**) The lentivirus infection efficiency and sequencing results of pHBLV, KRAS-WT, G12D, and G13D cells. Original magnification, ×20. (**B**) The expression level of ART1, HSC70, GRP78, and CHOP in pHBLV, KRAS-WT, G12D, and G13D cells. *P < 0.01 by 1-way ANOVA with Tukey’s HSD test (mean ± SEM, *n* = 3). (**C**) GST-mAf1521 pull-down assay was used to identify the existence of mono-ADP-ribosylated GRP78/BiP in G12D cells. (**D**) Effect of ART1 inhibitor MIBG on the level of mono-ADP-ribosylated GRP78/BiP in pHBLV, KRAS-WT, G12D, and G13D cells observed by far-Western blot. *P < 0.01 by 1-way ANOVA with Tukey’s HSD test (mean ± SEM, *n* = 3). (**E**) Effect of MIBG on the colocalization of GST-mAf1521 and GRP78/BiP in G12D cells. Original magnification, ×20 (left) and ×40 (right).

Since the experimental results are biased by other interfering factors when a variety of KRAS-mutant and -WT CRC cell lines are used as research subjects, we chose to construct KRAS-mutant cell lines by using the HT-29 cell line (KRAS WT), and 2 mutation sites with a relatively high probability of occurrence in CRC were selected. By fluorescence microscopy, nearly 100% of the cells in the 4 groups, pHBLV (empty load), KRAS WT (overexpression control), G12D (KRAS G12D site mutation), and G13D (KRAS G12D site mutation), presented green fluorescence. Sanger sequencing also revealed that the gene sequences in the pHBLV and KRAS-WT groups were identical to the KRAS-WT gene sequence, and G12D and G13D mutated from G to A at sites 35 and 38 of the KRAS gene, respectively (Figure 2A). These results indicated that the HT-29 KRAS-mutant cell line was constructed successfully. Moreover, consistent with the above conclusions, Western blot results revealed that, compared with those in KRAS-WT cells, the protein levels of ART1 and ERS markers were significantly increased in KRAS G12D and G13D mutant cells (*P* < 0.01) (Figure 2B). Then, the levels of arginine-MARylated proteins were determined by constructing an mAf1521 bait protein and performing macrodomain GST pull-down experiments. Coomassie brilliant blue staining revealed a distinct band at 78 kDa corresponding to the molecular weight of all possible MARylated proteins in G12D cells (Figure 2C). Additional far-Western blot detection revealed that the levels of arginine-MARylated GRP78/BiP in G12D and G13D cells were greater than those in the KRAS-WT group (*P* < 0.01). The level of arginine-MARylated GRP78/BiP in G12D and G13D cells was attenuated by treatment with the ART1 inhibitor *m*-iodobenzylguanidine hemisulfate salt (MIBG) (*P* < 0.01) (Figure 2D). Moreover, confocal microscopy revealed that GST-mAf1521 (red) can colocalize with GRP78/BiP (green) in the cytoplasm and membrane of G12D cells, but after MIBG treatment, although some of the colocalized proteins remained in the membrane, most of the modified GRP78/BiP in the cytoplasm had disappeared (Figure 2E).

### Attenuation of MARylated GRP78/BiP inhibits the growth of KRAS-mutant CRC cells both in vivo and in vitro.

**Figure 3:**
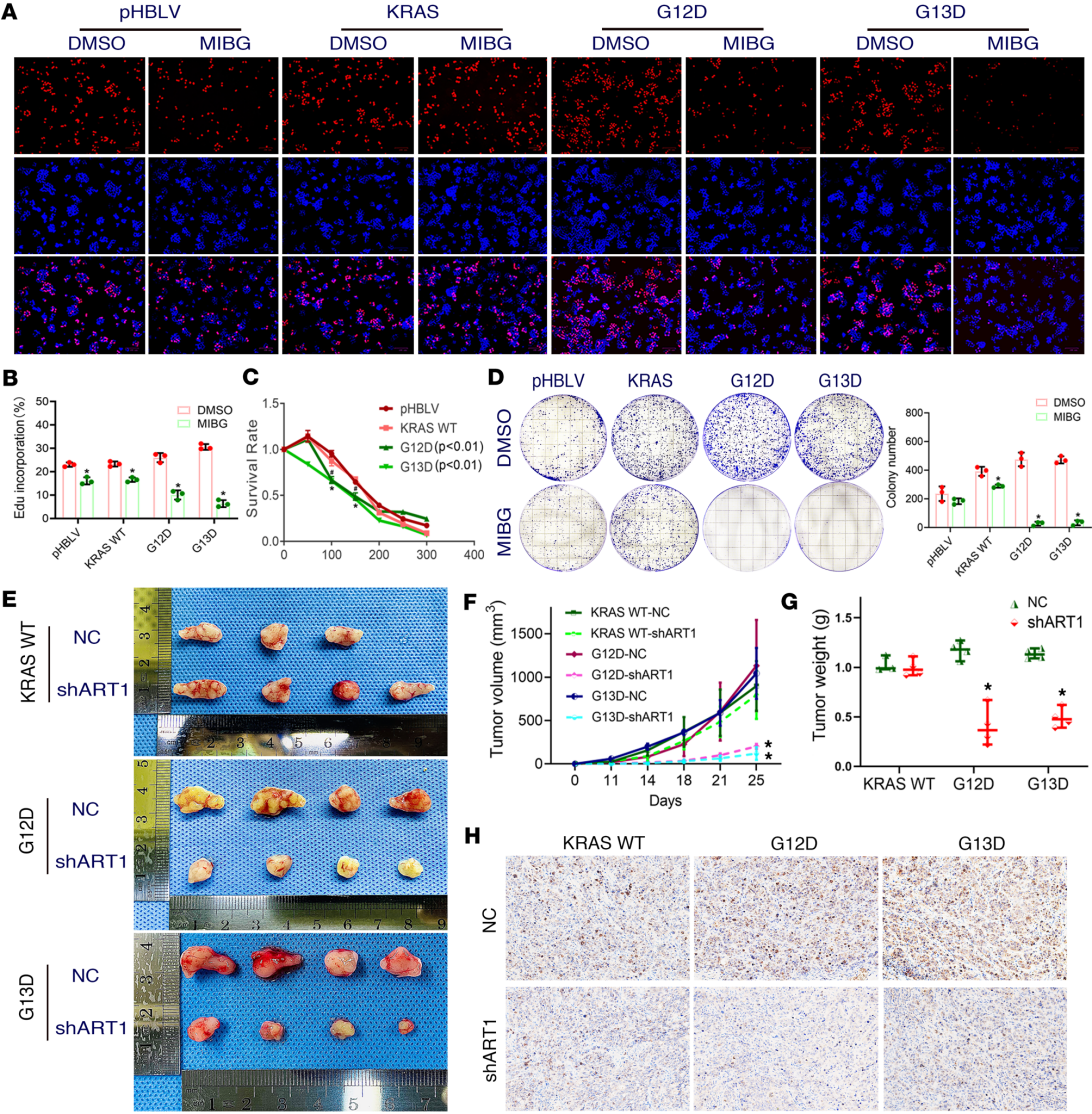
Inhibition of mono-ADP-ribosylated GRP78/BiP affects the growth of KRAS-mutant CRC cells. (**A** and **B**) The effects of ART1 inhibitor MIBG on the proliferation ability of pHBLV, KRAS-WT, G12D, and G13D cells. Original magnification, ×10. *P < 0.01 by *t* test (mean ± SEM, *n* = 3). (**C**) Survival rate of pHBLV, KRAS-WT, G12D, and G13D cells when treated with ART1 inhibitor MIBG. *P < 0.01 by *t* test (mean ± SEM, *n* = 3). (**D**) The effects of MIBG on the clone formation of pHBLV, KRAS-WT, G12D, and G13D cells. *P < 0.01 by *t* test (mean ± SEM, *n* = 3). (**E**) The size of subcutaneous xenograft tumors in KRAS-WT, G12D, and G13D groups after knocking down ART1 with lentiviral infection. The volume (**F**) and weight (**G**) of subcutaneous tumors in nude mice in KRAS WT-shART1, G12D-shART1, G13D-shART1 and KRAS WT-NC, G12D-NC, G13D-NC groups. *P < 0.01 by 1-way ANOVA with Tukey’s HSD test (mean ± SEM, *n* = 3). (**H**) The positive intensity of Ki67 staining in subcutaneously xenografted tumors of nude mice detected by immunohistochemistry after knocking down ART1 with lentiviral infection. Original magnification, ×200.

On the basis of the above studies, we aimed to further explore whether ART1 affects the growth and apoptosis of KRAS-mutant CRC cells by changing the level of arginine-MARylated GRP78/BiP. EdU-based cell proliferation experiments revealed that the number of proliferating cells in the G12D and G13D groups was greater than that in the pHBLV and KRAS-WT groups for the same initial cell number. Compared with those in the DMSO group, the cell proliferation abilities of the pHBLV, KRAS-WT, G12D, and G13D groups were significantly lower after MIBG treatment (*P* < 0.01). In particular, the reduction in red fluorescence in the G12D and G13D groups was far greater than that in the pHBLV and KRAS-WT groups (Figure 3, A and B). Additionally, through CCK8, we observed that after MIBG treatment, the number of surviving pHBLV, KRAS-WT, G12D, and G13D cells decreased significantly. However, the degree of cell growth inhibition in the G12D and G13D groups was significantly greater than that in the KRAS-WT group at concentrations of 100 μmol/mL and 150 μmol/mL (*P* < 0.01) (Figure 3C). These findings suggest that KRAS-mutated CRC cells are more vulnerable to ART1 inhibitors. Finally, we continued the plate cloning experiment to observe cell growth. The number of colonies formed increased when was KRAS mutated, and the number of colonies formed in the G12D and G13D groups was significantly lower than that in the DMSO group after 15 days of treatment with MIBG (*P* < 0.01) (Figure 3D).

**Figure 4:**
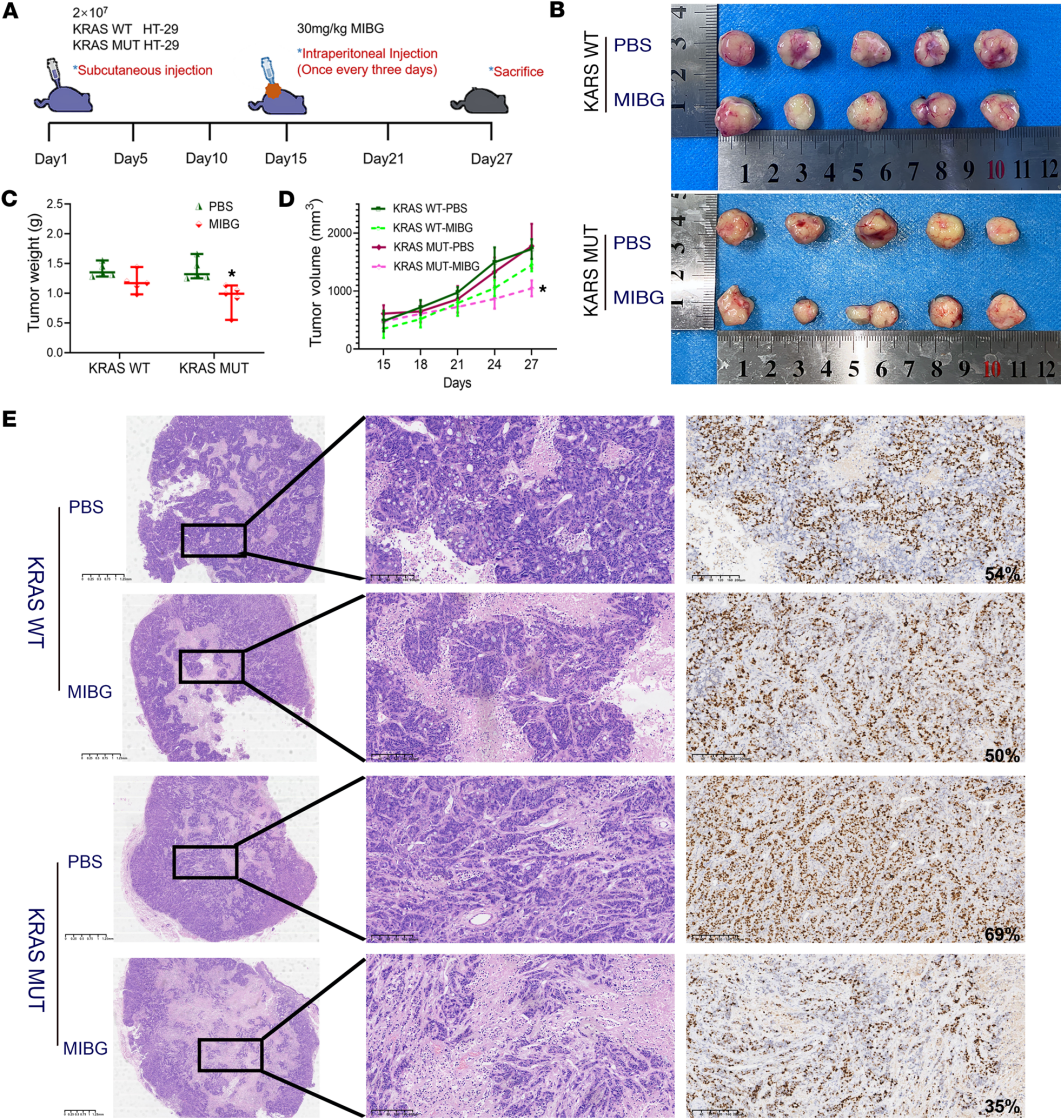
Effect of intraperitoneal injection of the ART1 inhibitor MIBG on the growth of KRAS-mutant and -WT CRC xenografts. (**A**) Schematic of transplanted-tumor growth and MIBG administration schedule. MIBG application concentration was 30 mg/kg. (**B**) The size of subcutaneous xenograft tumors in KRAS-WT and -mutant groups after inhibition of ART1 with MIBG treatment; PBS was used to treat the control group. The weight (**C**) and volume (**D**) of subcutaneous tumors in nude mice in 4 groups. *P < 0.01 by *t* test (**C**) or 1-way ANOVA with Tukey’s HSD test (**D**) (mean ± SEM, *n* = 3). (**E**) The H&E staining and positive intensity of Ki67 staining in subcutaneously xenografted tumors of nude mice after inhibition of ART1 with MIBG treatment. Scale bars: 1.25 mm (left column) and 200 μm (right columns).

In vivo, stable knockdown of ART1 in 3 groups, namely, the KRAS-WT, G12D, and G13D groups, was achieved by a knockdown lentivirus, and the groups were named KRAS WT-shART1, G12D-shART1 and G13D-shART1, respectively. The tumor volume and weight in the G12D-shART1 and G13D-shART1 groups were lower than those in the negative control (NC) group after 28 days of subcutaneous tumor growth (*P* < 0.01). However, the volume and weight of the transplanted tumors in the KRAS WT-shART1 group were not significantly different from those in the NC group (*P* > 0.05) (Figure 3, E–G). Furthermore, immunohistochemistry revealed that the percentage of Ki67-positive transplanted tumors in the G12D-shART1 and G13D-shART1 groups was lower than that in the G12D-NC and G13D-NC groups (*P* < 0.01) (Figure 3H and Table 6). The growth rate of the transplanted KRAS-mutant tumors was significantly lower after the intraperitoneal injection of MIBG than after the PBS treatment (*P* < 0.01) (Figure 4D). The volume and weight of the transplanted KRAS-mutant tumors resulting from MIBG injection (Figure 4A) were lower than those resulting from PBS injection (*P* < 0.01) (Figure 4, B and C). H&E staining revealed that after MIBG treatment, large areas of necrosis appeared in the center of the transplanted KRAS-mutant tumors (Figure 4E), and immunohistochemistry revealed that the percentage of the cell proliferation marker Ki67 was significantly decreased (Figure 4E).

### Attenuation of MARylated GRP78/BiP promotes the apoptosis of KRAS-mutant CRC cells both in vivo and in vitro.

**Figure 5:**
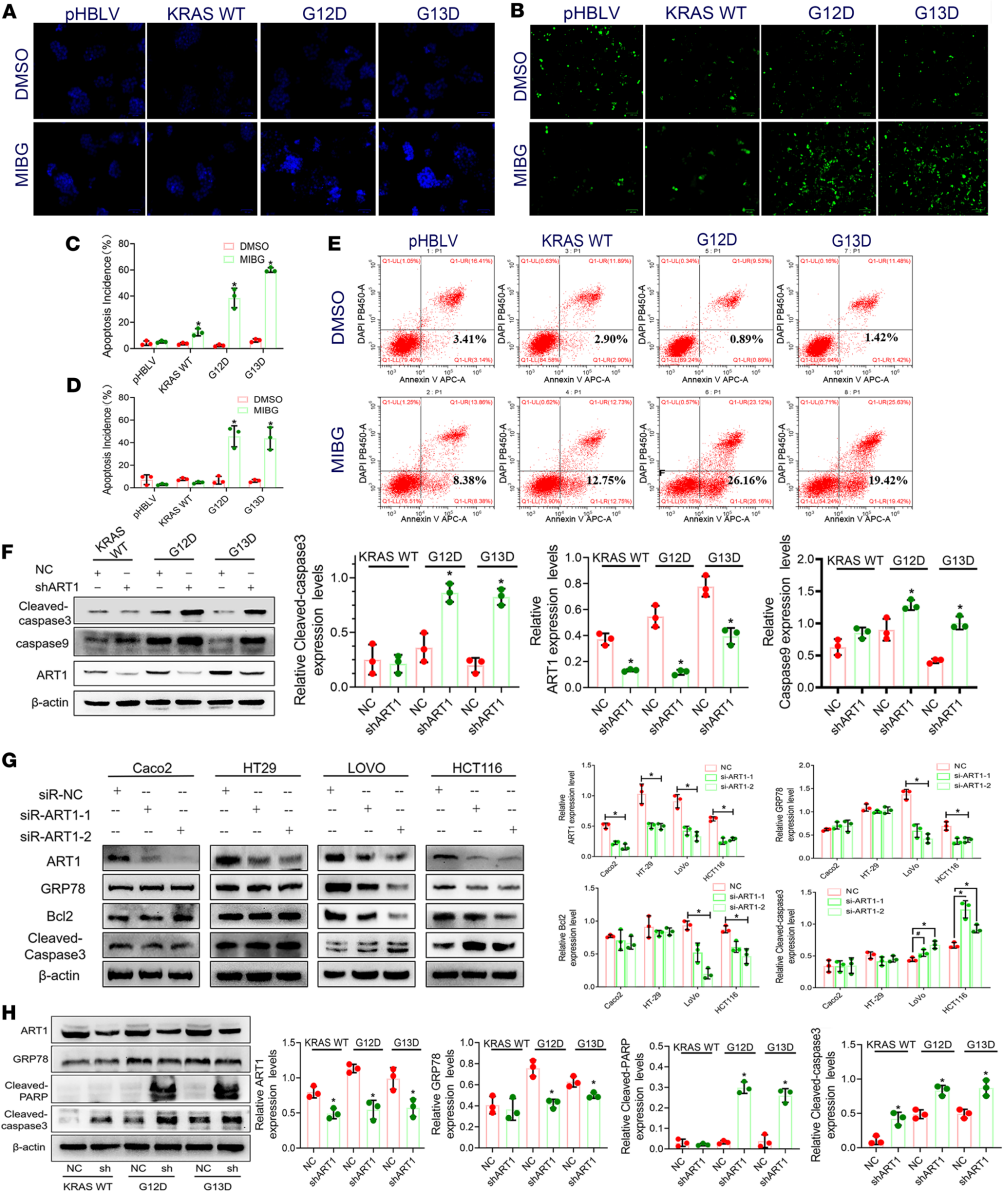
Inhibition of mono-ADP-ribosylated GRP78/BiP affects the apoptosis of KRAS-mutant CRC cells. (**A** and **C**) The number of apoptotic cells in pHBLV, KRAS-WT, G12D, and G13D after MIBG treatment detected by Hoechst staining. *P < 0.01 by *t* test, representative of 3 replicates. Scale bars: 100 μm. (**B** and **D**) The number of apoptotic cells in pHBLV, KRAS-WT, G12D, and G13D after MIBG treatment detected by TUNEL staining. *P < 0.01 by *t* test, representative of 3 replicates. (**E**) The apoptosis rate of pHBLV, KRAS-WT, G12D, and G13D cells after ART1 inhibitor MIBG treatment shown by Annexin V/PI double-staining flow cytometry. (**F**) The expression levels of apoptotic proteins cleaved caspase-3 and caspase-9 in KRAS-WT, G12D, and G13D cells after ART1 knockdown. *P < 0.01 by *t* test (mean ± SEM, *n* = 3). (**G**) The expression of ART1, GRP78, Bcl2, and cleaved caspase-3 in Caco2, HT-29, LoVo, and HCT116 cells with transfection of siART1. ^#^*P* < 0.05, *P < 0.01 by 1-way ANOVA with Tukey’s HSD test (mean ± SEM, *n* = 3). (**H**) The effect of ART1 knockdown on the expression of GRP78/BiP and cleaved caspase-3/PARP in subcutaneous tumors. *P < 0.01 by *t* test (mean ± SEM, *n* = 3).

Hoechst 33258 staining, TUNEL assays, and Annexin V/PI flow cytometry revealed that the number of apoptotic events in the G12D and G13D groups increased significantly after MIBG treatment. After Hoechst 33258 was used to label the nuclei in the G12D and G13D groups subjected to MIBG treatment, more nuclei were agglutinated, and stronger, fragmented, dense staining was observed (*P* < 0.01) (Figure 5, A and C). TUNEL apoptosis detection revealed that the proportion of apoptotic cells among the total cells increased significantly in the G12D and G13D groups of cells treated with MIBG (*P* < 0.01) (Figure 5, B and D). Annexin V/PI double-staining flow cytometry revealed that the percentage of apoptotic cells in the G12D and G13D groups increased from 0.89% and 1.42% to 26.16% and 19.42%, respectively, after MIBG was applied for 24 hours (Figure 5E). However, compared with the DMSO group, there was no corresponding change in the incidence of apoptotic events in the pHBLV and KRAS-WT groups when MIBG was applied. Compared with those in the G12D-NC and G13D-NC groups, the expression levels of cleaved caspase-3 and caspase-9 were significantly greater in the G12D-shART1 and G13D-shART1 groups (*P* < 0.01) (Figure 5F). Moreover, we tested the effect of ART1 knockdown on the apoptosis of different KRAS-mutant CRC cell lines. Western blot results showed that after ART1 was knocked down in LoVo and HCT116 (KRAS-mutant) cells, the expression of GRP78 and Bcl2 was significantly downregulated (*P* < 0.01), and the expression of cleaved caspase-3 was upregulated (*P* < 0.05) (Figure 5G). This change was not obvious in the 2 KRAS-WT cell lines (Caco2 and HT-29). In addition, the transcription levels of the key signaling molecules *BAK*, *BAX*, *BIM*, *NOXA*, and *CASP9* in the ER and mitochondrial apoptosis signaling pathways in LOVO and HCT-116 (KRAS-mutant) cells were significantly increased (*P* < 0.01), and the transcription levels of *Bcl2* and *Bcl-xL* were significantly decreased (*P* < 0.01) (Supplemental Figures 2 and 3; supplemental material available online with this article; https://doi.org/10.1172/jci.insight.182809DS1).

In vivo, Western blot analysis revealed that cleaved caspase-3 and cleaved PARP, both of which are core members of apoptosis, had significantly higher expression levels in the transplanted tumors of nude mice in the G12D-shART1 and G13D-shART1 groups than in those in the G12D-NC and G13D-NC groups (*P* < 0.01) (Figure 5H).

### MARylated GRP78/BiP affects the expression level of the GRP78/BiP protein in the KRAS-mutant state.

**Figure 6:**
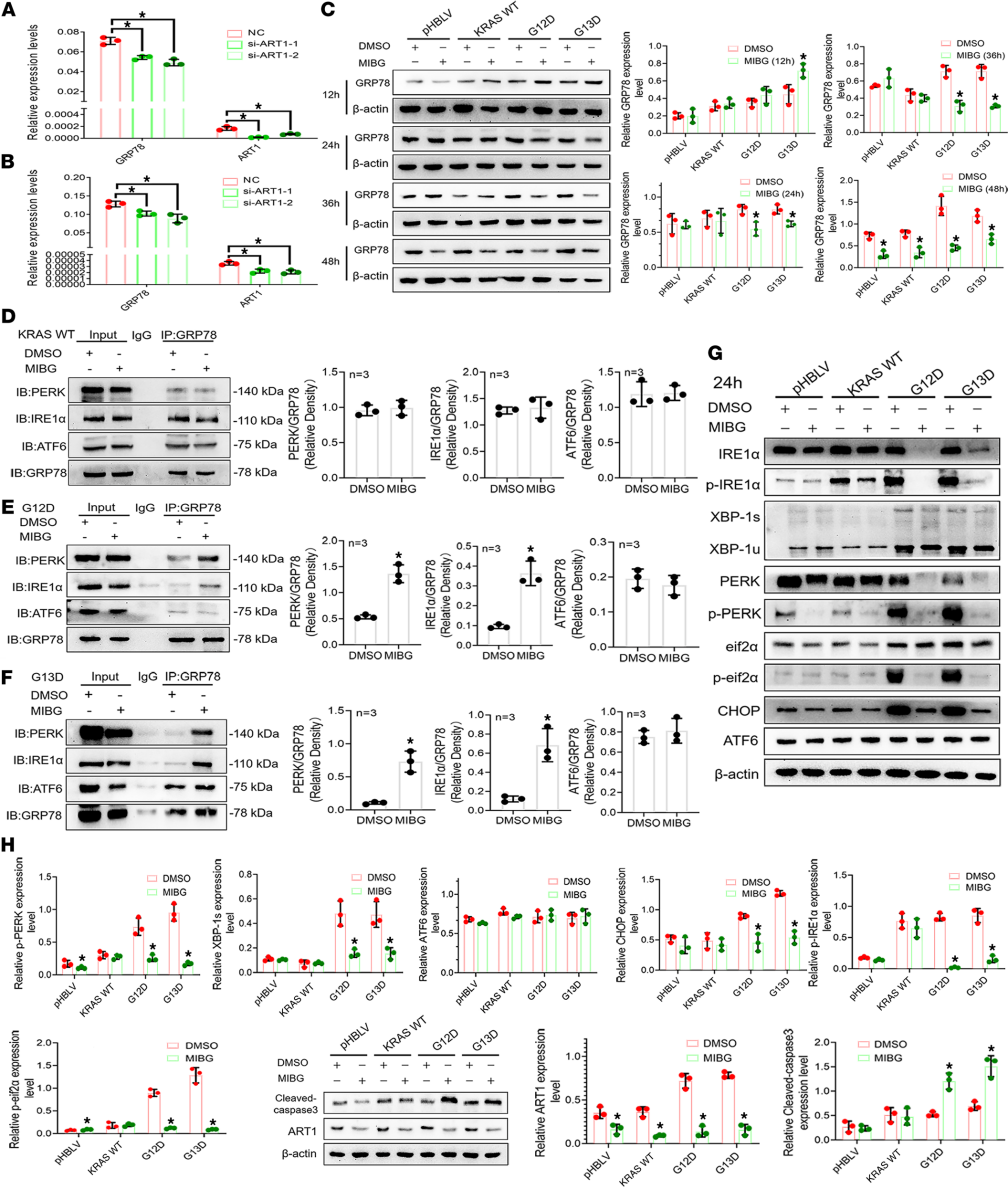
Molecular mechanism of mono-ADP-ribosylated GRP78/BiP affecting UPR signaling pathway. The mRNA levels of GRP78/BiP in LoVo (**A**) and HCT116 (**B**) cells after ART knockdown. *P < 0.01 by 1-way ANOVA with Tukeys HSD test (mean ± SEM, *n* = 3). (**C**) The protein expression levels of GRP78/BiP in pHBLV, KRAS-WT, G12D, and G13D cells treated with ART1 inhibitor MIBG for 12, 24, 36, and 48 hours. *P < 0.01 by *t* test (mean ± SEM, *n* = 3). Effects of GRP78/BiP arginine mono-ADP-ribosylation modification by MIBG on the binding of GRP78/BiP to its receptors PERK (**D**), IRE1α (**E**), and ATF6 (**F**) in KRAS-WT, G12D, and G13D cell lines. *P < 0.01 by *t* test (mean ± SEM, *n* = 3). (**G** and **H**) The expression levels of key proteins in the UPR signaling pathways IRE1α/XBP1/TFAF2/JNK, PERK/eIF2α/ATF4, and ATF6/S1P/S2P/CHOP in pHBLV, KRAS-WT, G12D, and G13D cells treated with MIBG. *P < 0.01 by *t* test (mean ± SEM, *n* = 3). (**I**) The effect of ART1 inhibitor MIBG on the expression of cleaved caspase-3 in KRAS-WT, G12D, and G13D cells. *P < 0.01 by *t* test (mean ± SEM, *n* = 3).

In the above studies, we determined that more arginine-MARylated GRP78/BiP existed in the KRAS-mutant state, which stabilized the continuous growth of the mutant cells. Next, we conducted a further detailed study on the molecular mechanism involved. The expression level of the GRP78/BiP protein changed significantly when arginine MARylation of GRP78/BiP occurred. The Western blot results revealed that the expression of GRP78/BiP was significantly inhibited in LoVo and HCT116 cells after ART1 was knocked down by siRNA (*P* < 0.01) (Figure 5G). The change at the gene level was consistent with the change at the protein level. Real-time PCR revealed that the expression level of *GRP78/BiP* mRNA was significantly lower than that in the control group (*P* < 0.01) (Figure 6, A and B). Cells in the pHBLV, KRAS-WT, G12D, and G13D groups were further treated with the ART1 inhibitor MIBG for 12, 24, 36, and 48 hours, and the expression levels of GRP78/BiP in G12D and G13D cells increased slightly at 12 hours. The level of GRP78/BiP expression began to decrease significantly at 24 hours with ART1 inhibitor treatment, which lasted until 36 and 48 hours (*P* < 0.01). However, the level of GRP78/BiP expression did not change at 12, 24, or 36 hours in pHBLV and KRAS-WT cells treated with the ART1 inhibitor, and a significant decrease in the level of GRP78/BiP expression was observed at 48 hours (*P* < 0.01) (Figure 6C).

### MARylated GRP78/BiP regulates the IRE1α/XBP1/TFAF2/JNK and PERK/eIF2α/ATF4 pathways by affecting the association and dissociation interactions between GRP78 and its interactors.

Studies have shown that GRP78/BiP associates with or dissociates from its interactors IRE-1α, ATF6, and PERK located in the ER membrane to regulate the IRE1α/XBP1/TFAF2/JNK, PERK/eIF2α/ATF4, and ATF6/S1P/S2P/CHOP signaling pathways (22, 23). In general, the activation of these 3 signaling pathways results in the production of more GRP78/BiP, which ultimately relieves the pressure of excess unfolded protein on tumor cells through the binding of GRP78/BiP to unfolded protein. In this study, we investigated whether MARylated GRP78/BiP regulates the IRE1α/XBP1/TFAF2/JNK and PERK/eIF2α/ATF4 pathways by affecting the association and dissociation interactions between GRP78/BiP and its interactors. As shown in Figure 6, D–F, the interaction between PERK and IRE1α and GRP78/BiP was significantly increased in G12D and G13D cells when an ART1 inhibitor was used to interfere with the modification of GRP78/BiP (*P* < 0.01), and the binding state between GRP78/BiP and the ATF6 protein did not change significantly (*P* > 0.05). However, in the KRAS-WT group, the binding of GRP78/BiP to PERK, IRE1α, and ATF6 did not change significantly (*P* > 0.05). Next, we detected the activation status of the 3 signaling pathways involved in the UPR. We first observed that the expression levels of p-PERK, p-eIF2α, and p-IRE1α were significantly increased in G12D and G13D cells compared with those in the KRAS-WT group. According to the coimmunoprecipitation (co-IP) results, more GRP78/BiP interacted with IRE1α and PERK receptors when ART1 inhibitors were applied to G12D and G13D cells; therefore, the IRE1α/XBP1/TFAF2/JNK and PERK/eIF2α/ATF4 signaling pathways were not activated. As shown in Figure 6, G and H, compared with those in the DMSO group, the expression levels of PERK, p-PERK, p-eIF2α, IRE1α, p-IRE1α, XBP1u/s, CHOP, and GRP78 were significantly lower, and the expression level of ATF6 did not significantly change. In contrast, the IRE1α/XBP1/TFAF2/JNK and PERK/eIF2α/ATF4 signaling pathways did not show changes similar to those in KRAS-mutant cells after MIBG was applied to pHBLV and KRAS-WT cells. Finally, the expression level of the apoptotic protein cleaved caspase-3 was significantly increased after G12D and G13D cells were treated with the ART1 inhibitor MIBG (Figure 6I). In addition, we knocked down ART1 by using 3 different siRNA sequences and detected the inactivation of the IRE1α/XBP1/TFAF2/JNK and PERK/eIF2α/ATF4 signaling pathways again (Supplemental Figures 4 and 5). Therefore, ART1 can affect the binding of GRP78/BiP to PERK and IRE1α by catalyzing the arginine MARylation modification of GRP78/BiP in KRAS-mutant CRC cells, thereby participating in the regulation of the UPR signaling pathway. Assuming that KRAS-mutant cells need more arginine-MARylated GRP78 to relieve the pressure of unfolded proteins, KRAS-mutant CRC cells are more sensitive to the application of ART1 inhibitors.

### MARylation at Arg214 of GRP78/BiP is involved in mediating ERS in KRAS-mutant cells.

**Figure 7:**
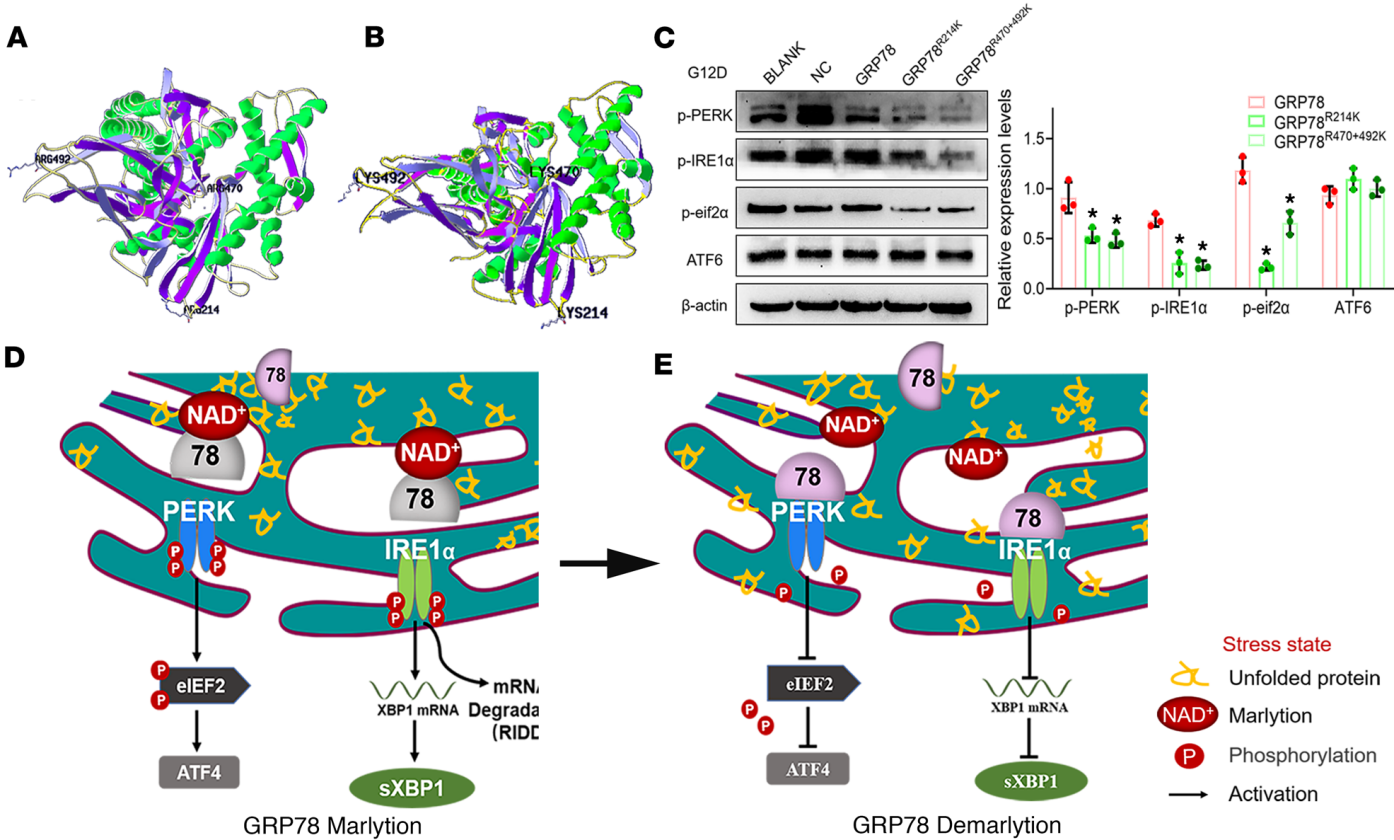
Preliminary study on arginine mono-ADP-ribosylation site of GRP78/BiP. (**A**) The position of arginines 470, 492, and 214 on the GRP78 protein. (**B**) Three-dimensional protein structure of GRP78 after arginines 470, 492, and 214 were mutated to lysine. (**C**) Effects of downstream UPR signaling pathways after GRP78/BiP^R470+492K^ and GRP78/BiP^R214K^ plasmids transfected into G12D cells. *P < 0.01 by 1-way ANOVA with Tukey’s HSD test (mean ± SEM, *n* = 3). (**D**) Schematic diagram of arginine–mono-ADP-ribosylated GRP78/BiP involved in the regulation of ER homeostasis under the state of ER stress caused by KRAS mutation: ART1 catalyzes the arginine mono-ADP-ribosylation of GRP78/BiP to inactivate it and release the receptors bound to it. The downstream UPR signaling pathway is continuously activated to help maintain the homeostasis of the ER and the survival of tumor cells. (**E**) Schematic diagram of the effect of interference with arginine–mono-ADP-ribosylated GRP78/BiP on the UPR signaling pathway. Interfering with GRP78/BiP arginine mono-ADP-ribosylation modification can downregulate the expression of GRP78/BiP; limited GRP78/BiP can bind to unfolded protein and sensors PERK and IRE1α, inhibiting the IRE1α/XBP1/TFAF2/JNK pathway. The PERK/eIF2α/ATF4 signaling pathway further leads to the reduction of GRP78 expression, damages the ER stress regulation mechanism, increases the pressure of the internal environment, hinders the growth of tumor cells, and the cells tend to undergo apoptosis.

Since the effect of MARylation is closely related to the modification site, this modification has different functional effects because of the different substrate proteins and modification sites used (17, 24). We further detected the modification site of GRP78/BiP in the KRAS-mutant CRC cell lines SW480 and LoVo by using matrix-assisted laser desorption/ionization–time-of-flight mass spectrometry (MALDI-TOF MS) and detected MARylation at Arg214 of the GRP78/BiP protein in the 2 cell lines (Table 7). In addition, Chambers et al. (19) reported that this modification also occurs at Arg470 and Arg492 on the GRP78/BiP protein of 293T cells via MALDI-TOF MS and that Arg492 is necessary for the function of Arg470. Therefore, we constructed mutant-overexpressing plasmids that simultaneously mutated Arg470 and Arg492 on GRP78/BiP to lysine or mutated Arg214 to lysine alone (Figure 7, A and B). Plasmid transfection followed by Western blot detection revealed that the expression level of the key proteins p-PERK, p-eIF2α, and p-IRE1α in the UPR signaling pathway was significantly lower in the GRP78/BiP^R470+492K^ and GRP78/BiP^R214K^ groups than in the control group (*P* < 0.01) (Figure 7C). These findings indicate that MARylation at Arg470, Arg492, and Arg214 of GRP78/BiP plays a certain role in the regulation of ERS in KRAS-mutant CRC.

## Discussion

Most CRCs are already at an advanced stage or have metastasized when they are discovered. In recent years, the application of molecular targeted drugs has increased in popularity, and chemotherapy combined with targeted therapy has become the first choice to improve the OS of patients with CRC. However, the mutation status of the KRAS gene is the most critical reference factor in the selection of targeted drugs for the treatment of CRC (25, 26), and patients with KRAS mutations develop comprehensive resistance to anti-EGFR drugs and have a shorter survival time and poorer prognosis. Similarly, comparisons of OS, PFS, DFS, and DSS between patients with KRAS-mutant and -WT CRC revealed that the OS and PFS of patients with KRAS-mutant CRC were significantly shorter than those of patients with KRAS-WT CRC. Currently, the notion that there are no available drugs to treat patients with KRAS mutations is very controversial not only because of the influence of a single factor of the KRAS mutation itself but also because other intracellular changes caused by KRAS mutations may be key factors shortening patient survival. Therefore, this study aimed to focus on the molecular biological responses of tumor cells driven by KRAS gene mutations in CRC and to conduct in-depth exploration, which can provide clues and lay the foundation for the development of targeted therapies.

KRAS mutations impair GTPase activity, which in turn irreversibly activates the KRAS protein, resulting in overactive signals that initiate and maintain tumorigenesis. KRAS-driven abnormal proliferation requires considerable protein synthesis, resulting in ER overload and the accumulation of unfolded/misfolded proteins. The only way to cope with synthetic stress is to continuously activate the UPR and promote the cascade of downstream sensors such as PERK, IRE1α, and ATF6 (12). For example, the overexpression of KRAS-mutant protein increases the ERS level in pancreatic cancer cells, which enables the further development of pancreatic tumors (27). GRP78/BiP is the most important marker that reflects the stress level of the ER, which can be automatically combined with polypeptide chains in the ER in the form of noncovalent signals to promote the correct folding and assembly of proteins (28–30), and high levels of GRP78/BiP expression have been reported in various tumor tissues, such as those of CRC, pancreatic cancer, breast cancer, and prostate cancer (31). However, only Shen et al. (32) have explored the difference between the expression levels of GRP78/BiP in mouse KRAS-mutant pancreatic cancer and KRAS-WT pancreatic cancer, and the results revealed that the expression of GRP78/BiP in mouse KRAS^G12D^-mutant pancreatic cancer tissues was significantly greater than that in KRAS-WT pancreatic cancer tissues. However, whether KRAS mutation can alter the level of ERS in CRC remains unclear. This study revealed that in CRC, the expression of the ERS markers GRP78/BiP, HSC70, and CHOP was increased in KRAS-mutant tumors. HSC70 is a molecular chaperone involved in the folding and transportation of polypeptides in the ER and plays an important role in ensuring the correct folding process, and CHOP, as a multifunctional transcription factor in the ERS response, responds to multiple stimuli in a timely manner; moreover, CHOP is abundantly expressed in the cytoplasm under normal physiological conditions and can enter the nucleus in large quantities and act as a transcription factor to participate in the regulation of the internal environment of the ER when ERS is activated (33, 34). Because the degree of ER swelling can reflect the degree of ERS to a certain extent (35, 36), we also observed obvious swelling of the ER in LoVo, HCT116, and SW480 cells via transmission electron microscopy, suggesting that the KRAS mutation status of CRC can trigger an increase in the level of ERS. Arginine MARylation is the most vital way to regulate the activity and function of GRP78/BiP. According to the GeneCards database, the arginine-MARylating enzyme ART1 is abundant in the ER, and studies have shown that ART1 colocalizes with GRP78/BiP (21). There is currently no research on the effect of KRAS mutation on MARylation modification in lung adenocarcinoma or pancreatic cancer patients with KRAS mutations. On the basis of the solid research foundation of our research group on MARylation, we will first conduct this study in patients with CRC with KRAS mutations. The in vivo and in vitro experiments suggest that arginine MARylation is related to KRAS-mutant CRC, which may play a role by affecting the occurrence of the arginine MARylation of GRP78/BiP through ART1 catalysis.

During tumor growth, sustained ERS leads to rapid cellular tolerance and confers greater viability to malignant cells (37, 38). Therefore, because the levels of ART1 and ERS markers in KRAS-mutant CRC are greater than those in KRAS-WT CRC, ART1 may alter the growth and apoptosis of KRAS-mutant CRC cells by affecting the level of arginine-MARylated GRP78/BiP. Owing to the lack of specific detection tools, the current research on the target protein of MARylation and its modification level is lacking and has focused mainly on the enzyme that catalyzes the modification. However, Dani et al. identified a fusion protein, mAf1521, capable of interacting with MARylated proteins using the specific protein module “macro domain,” which provides a better method for detecting ribosylated proteins modified by endogenous ARTs and their modification levels (39, 40). In addition, Dani et al. reported that the MARylated-GRP78/BiP level could be changed significantly by the overexpression or knockdown of ART1, suggesting that MARylation of the GRP78/BiP protein occurs at arginine(s) and is catalyzed by ART1. Therefore, this study fully exploited the ability of the fusion protein mAf1521 to bind to MARylated proteins and detected the level of arginine-MARylated GRP78/BiP in KRAS-mutant CRC cells by GST pull-down and far-Western blot analyses. The results showed that after the application of the ART1 inhibitor MIBG, the level of the arginine-MARylated GRP78/BiP protein in the KRAS G12D and G13D cells was significantly reduced. More importantly, in the subsequent detection of growth and apoptosis in vivo and in vitro, although inhibiting ART1 can inhibit the growth of KRAS-WT, G12D, and G13D cells, the inhibition of G12D and G13D growth was more obvious in the mutant group than in the WT group, and compared with KRAS-WT CRC cells, KRAS-mutant CRC cells experience greater apoptotic responses after ART1 inhibitor treatment. These results indicate that the application of an ART1 inhibitor may weaken the process by which the ERS centered on GRP78/BiP is regulated and that KRAS-mutant CRC cells with higher levels of ERS are more vulnerable to altered levels of MARylated GRP78/BiP.

Next, we explored the molecular mechanism by which ART1 affects the level of MARylated GRP78/BiP and further affects the proliferation and apoptosis of KRAS-mutant CRC cells. Studies have shown that application of an ERS activator (tunicamycin) increases the expression level of ART1 and the level of MARylated GRP78/BiP, which is the first response of HeLa cells to stress, and then manifests as the upregulation of GRP78/BiP expression, which promotes the homeostasis of the ER and is beneficial for cell growth (21). In this study, ART1 inhibitors could downregulate the expression of GRP78/BiP earlier in KRAS-mutant CRC cells than in KRAS-WT CRC cells, indicating that KRAS-mutant CRC cells are more sensitive to ART1 inhibitors. In addition, more GRP78/BiP can attach to unfolded proteins and dissociate from the 3 receptors PERK, IRE1α, and ATF6, thereby activating the UPR when tumor cells are in a state of ERS in the face of various sudden stimuli and further generate more GRP78/BiP to bind to unfolded proteins through the IRE1α/XBP1/TFAF2/JNK, PERK/eIF2α/ATF4, and ATF6/S1P/S2P-CHOP cascades, subsequently relieving ER pressure to promote ER homeostasis and accelerating tumor progression (41–43). The IRE1α signaling pathway activates and upregulates the transcription of genes related to protein folding, transport, and degradation and reduces the protein load of the ER by mediating the inhibition of mRNA transcription, which is called regulated IRE1-dependent decay (IRE1-dependent decay, RIDD) (44, 45). Activated PERK mediates NF-κB phosphorylation through the activation of eIF2α, inhibits the expression of apoptosis-related proteins, and promotes the expression of cell cycle proteins to prevent apoptosis and promote cell growth (46, 47). In our previous research, we reported that the differentially expressed genes produced by knocking down ART1 in mouse CRC CT-26 cells could be enriched in the ER protein synthesis signaling pathway in KEGG, and gene enrichment analysis revealed that the UPR signaling pathway was significantly downregulated. Therefore, the effect of MARylated GRP78/BiP on KRAS-mutant CRC cells also depends on the regulatory effect of ART1 on the binding and dissociation of GRP78/BiP and its receptors, as well as the regulation of the UPR signaling pathway. Therefore, more PERK and IRE1α can interact with GRP78/BiP when ART1 is inhibited, and the extent of binding between ATF6 and GRP78/BiP did not change significantly before and after ART1 inhibition in KRAS G12D and G13D cells. In addition, in KRAS-WT cells, the binding of PERK and IRE1α to GRP78/BiP did not change significantly after ART1 inhibition. These findings indicate that ART1 affects the binding of GRP78/BiP to PERK and IRE1α, thereby participating in the regulation of the UPR signaling pathway in KRAS-mutant CRC cells.

The effect of MARylation is closely related to its modification site, and this modification will produce different functional effects due to different substrate proteins and modification sites (17, 24). Chambers et al. discovered this modification at Arg470 and Arg492 of the GRP78/BiP protein in 293T cells by MALDI-TOF MS, and Arg492 is necessary for the function of the Arg470 modification (19). However, to date, there has been no corresponding research on the specific site of arginine MARylation on GRP78/BiP in CRC cells. In this study, through a preliminary study via MALDI-TOF MS, we revealed that arginine MARylation occurs at Arg214 of the GRP78/BiP protein in the human CRC cell lines LOVO and SW480, which carry the mutated KRAS gene. Therefore, we further mutated Arg214, Arg492, and Arg470 of the GRP78/BiP protein to lysine, and subsequent detection of key proteins in the UPR signaling pathway also revealed that the IRE1α/XBP1/TFAF2/JNK and PERK/eIF2α/ATF4 signaling pathways were significantly inhibited. These findings suggest that MARylation at Arg470, Arg492, and Arg214 of GRP78/BiP plays a vital role in the regulation of ERS in KRAS-mutant CRC. However, the major and minor relationships and the degree of strength of these sites when they play a role in the regulation of the UPR signaling pathway still need further research and verification.

### Conclusion.

In KRAS-mutant cells, more MARylated GRP78/BiP assists the ER in relieving the pressure of unfolded proteins, whereas interfering with the modification of the GRP78/BiP protein can inhibit the IRE1α/XBP1/TFAF2/JNK and PERK/eIF2α/ATF4 signaling pathways by promoting GRP78/BiP binding to PERK and IRE1α, thereby inhibiting the growth of KRAS-mutant CRC cells and promoting their apoptosis. Moreover, compared with KRAS-WT CRC cells, KRAS-mutant cells were more sensitive to intervention. The research presented in this paper aims to provide ideas and a certain experimental basis for the targeted therapy of KRAS-mutant CRC (Figure 7, D and E).

## Methods

### Sex as a biological variable.

Only female nude mice were used for subcutaneous xenografts in order to minimize hormonal variability and aggressive behavior and ensure consistency in tumor growth kinetics. The findings are expected to be relevant for more than one sex.

### Reagents.

A streptomycin-biotin-peroxidase immunohistochemical staining kit was purchased from Maxim Biological Technology Corporation (catalog KIT-9710). BeyoClick EdU Cell Proliferation Kit with Alexa Fluor 594 (catalog C0071S), One Step TUNEL Apoptosis Assay Kit (catalog C1090), Cell Counting Kit (catalog C0038), and Hoechst 33258 (catalog C1017) were obtained from Beyotime Technologies. Lipofectamine 2000 (catalog 11668) was obtained from Thermo Fisher Scientific. MIBG (dissolved in DMSO) was purchased from Sigma-Aldrich (catalog I9890) for inhibiting ART1. The following antibodies were used: ATF6 (catalog 3192), PERK (catalog 65880), IRE1α (catalog 3294), eIF2α (catalog 5324), p-eIF2α (catalog 3398) (all Cell Signaling Technology); GRP78/BiP (catalog sc-13539), HSC70 (catalog sc-24), Bcl2 (catalog sc-7382) (all Santa Cruz Biotechnology); p-IRE1α (catalog ab124945), XBP1u/s (catalog ab220783) (both Abcam); p-PERK (catalog DF7576, Affinity Biosciences); CHOP (catalog NB600-1335SS, Novus Biotechnology); ART1 (catalog A10103, Abclonal Technology Co., Ltd.); Caspase-3 (catalog WL02117), PARP/cleaved-PARP (catalog WL01932) (both Wanlei Biotechnology); β-actin (catalog 6609-1-Ig), Ki67 (catalog 27309-1-AP) (both Proteintech). Anti-rabbit, anti-rat, and anti-mouse secondary antibodies (Proteintech) were also obtained. 4,6-Diamidino-2-phenylindole (DAPI), FITC-conjugated anti-rabbit IgG antibody, and FITC-conjugated anti-mouse IgG antibody were purchased from Proteintech.

### Survival analysis based on KRAS mutation status.

The clinical information, mutation status of KRAS, whole-transcriptome sequencing data, and gene mutation information of 534 patients were obtained from the Colorectal Adenocarcinoma (TCGA, PanCancer Atlas) dataset in the Cbioportal database (https://www.cbioportal.org). Among 534 patients with CRC, KRAS was mutated in 218 tissues, and KRAS was WT in 316 cases. The drawing of survival curves, the classification of KRAS mutation types, the statistics of accompanying gene mutations, and the analysis of differentially expressed genes were all analyzed online in the Cbioportal database.

### Human samples and immunohistochemical staining.

We randomly selected 79 formalin-fixed, paraffin-embedded (FFPE) samples from patients with CRC from the Department of Pathology of Chongqing Medical University, and the KRAS mutation status was confirmed by the patient’s individualized tumor therapy target test report and Sanger sequencing simultaneously. NucleoSpin DNA FFPE XS (catalog MNG-740980.250, MACHEREY-NAGEL) was used for DNA extraction from paraffin sections in Sanger sequencing.

Immunohistochemical staining was performed with an Ultra-Sensitive SP (Mouse/Rabbit) IHC Kit according to the manufacturer’s instructions (Maixin Biotech). In brief, the section was deparaffinized and pressurized in EDTA (15 minutes) antigen retrieval solution to expose the antigen, then incubated with primary antibody against GRP78 (1:500), HSC70 (1:500), CHOP (1:200), ART1 (1:500), or Ki67 (ready-to-use) at 4°C overnight. Finally, 3,3′-diaminobenzidine (DAB) was used as a chromogenic agent to indicate positive staining. The staining intensity and percentage of positive cells in immunohistochemical results were scored according to the method of Fromowitz et al. (48). For the proportion (%) of positive tumor cells per 100 tumor cells observed under low magnification, <5% positive = 0 points, 5%–25% positive = 1 point, 26%–50% positive = 2 points, 51%–75% positive = 3 points, and 76%–100% positive = 4 points. For staining intensity, no coloring = 0 points, light yellow = 1 point, brownish yellow = 2 points, and dark brown = 3 points. The above two were multiplied to obtain a positive grade: 0, negative (–); 1–4, weakly positive (+); 5–8 moderately positive (++); and 9–12, strongly positive (+++). This process was carried out double-blindly by 2 pathologists, and the average value was used for statistics.

### Electron microscopy.

SW48, Caco2, HT-29, HCT116, LoVo, and SW480 cells in culture (not less than 1 × 10^7^ per flask) were digested with trypsin, centrifuged at 160*g* for 5 minutes, supernatant discarded, and 2.5% ice-cold glutaraldehyde fixative slowly added and then incubated at 4°C for 48 hours. Afterwards, samples were sent to the Electron Microscopy Lab of Chongqing Medical University, then embedded, sectioned, stained with uranyl acetate and lead citrate, and finally observed with a transmission electron microscope (Hitachi).

### Cell culture and the construction of G12D- and G13D-mutated KRAS CRC cell lines.

Human CRC cell lines SW48 (KRAS WT), HT-29 (KRAS WT), SW480 (G12V), and HCT116 (G13D) were obtained from American Type Culture Collection (ATCC). The Lovo (G13D+A14V) cell line was donated by Wei-Xue Tang (Chongqing Medical University), and the Caco2 (KRAS WT) cell line was a gift from Tingxiu Xiang (The First Affiliated Hospital of Chongqing Medical University). SW48, HT-29, SW480, HCT116, and Caco2 cell lines were routinely cultured in Roswell Park Memorial Institute 1640 (RPMI-1640) medium supplemented with 10% fetal bovine serum (FBS) and 1% penicillin-streptomycin at 37°C in a humidified incubator with 5% CO_2_.

The first 2 mutation sites with the highest KRAS gene mutation rate in CRC were obtained from the International Cancer Genome Consortium (ICGC) database (https://dcc.icgc.org/), which were G12D (the 35th base in codon 12 is mutated from G to A) and G13D (the 38th base in codon 13 is mutated from G to A). Lentiviruses carrying mutated KRAS genes were commercially constructed by HanBio Technology. Lentiviral vectors were produced by using a 3-plasmid system (LV, psPAX2, pMD2G).

To generate a G12D- and G13D-mutated KRAS CRC cell line, a cell multiplicity of infection of 10 was used to calculate the volume of virus, and 3 × 10^5^ cells (12-well plate) were infected at 30%–40% confluence. The virus-infected cells were selected with 8 μg/mL puromycin for 2 weeks, during which the fluorescence brightness and percentage were continuously observed, and recorded with Bio-Rad fluorescence microscope (ZOE Fluorescent Cell Imager). The constructed KRAS-mutant cells were retained as cell pellets (at least 1 × 10^7^ cells per group) and sent to Sangon Biotechnology for Sanger sequencing to confirm that the mutations were successfully constructed.

### siRNA transfection and MIBG treatment.

Cells in logarithmic growth phase were digested with trypsin into a single-cell suspension, seeded in a 6-well plate at a density of 5 × 10^4^ cells per well, and cultured at 37°C with 5% CO_2_ until the cell fusion degree reached 30%–50%. siRNAs against ART1 (siART1), negative control (NC) siRNAs, and Lipofectamine 2000 were mixed separately with serum-free medium and incubated 5 minutes at room temperature. siART1 and NC siRNAs were then added to separate mixtures of Lipofectamine 2000/serum-free medium, mixed 8–10 times, and then added to the cells. Six hours after transfection, the fresh complete medium was replaced, and the cells were collected 48 hours later. The synthesis of the 3 siART1 and NC siRNAs required for the experiment was completed by HanBio technology Co., Ltd. The sequences are shown in Table 8.

The inhibitory effect of MIBG on ART1 was detected by Western blot, and the concentration gradient was set as 0, 50, 100, 150, 200, and 250 μmol/L. The results showed that at a concentration of 100 μmol/mL, the expression of ART1 in HT-29 cells was significantly inhibited. Combined with the IC_50_ values of each group of cells, the final concentration of 100 μmol/L was selected for subsequent experiments.

### Western blot analysis.

For protein analysis, the cells of each group were fully lysed with RIPA buffer supplemented with protease inhibitors and phosphatase inhibitors at a ratio of 1:100, fully pipetted and lysed on ice, and then the supernatant was extracted after being centrifuged at 13,800*g* for 15 minutes. The protein concentration was determined using a BCA Protein Assay Kit (Beyotime, catalog P0012S). Subsequently, 30 μg of protein per group was added to the well of an SDS-PAGE gel, and Western blot was performed on a Bio-Rad Powerpac HC PowerSupply. Gels were electrophoresed at a constant voltage of 80 V for 30 minutes followed by 110 V for 90 minutes and then transferred onto the nitrocellulose membrane at 0.3 A for 2 hours. Antibody binding was visualized on Bio-Rad Chemidoc MP Western multifunctional imaging analyzer.

### Macrodomain GST pull-down assay and far-Western blot analysis.

Taking advantage of the property that the fusion protein mAf1521 can bind to MARylated proteins, the levels of arginine-MARylated GRP78/BiP in KRAS-WT and -mutant CRC cells were detected by macrodomain GST pull-down and far-Western blot (39, 49). The amino acid sequence of the Af1521 bait protein derived from *Archaeoglobus* *fulgidus* was obtained from the NCBI database (WP_048064404.1) and synthesized by Abiocenter Biotechnology Co., Ltd.

Macrodomain GST pull-down assay was performed with a Pierce GST Protein Interaction Pull-Down Kit according to the manufacturer’s protocol. Equal amounts of mAf1521 protein (conjugated on beads) were incubated with 1 mL cell lysates with end-over-end rotation at 4°C overnight. The beads were washed with glutathione in Tris-buffered saline (TBS) solution and were eluted from the beads with 2× SDS-PAGE sample buffer and detected by Coomassie blue staining.

For far-Western blot analysis, during electrophoresis, the total amount of sample loaded in each well was 100 μg. After transfer to a PVDF membrane, the membrane was incubated with 20 ng/mL glutathione *S*-transferase–labeled (GST-labeled) mAf1521 protein in TBS buffer at 4°C overnight. After blocking with 5% FBS for 1 hour at room temperature, the membrane was washed 3 times. Subsequently, the PVDF membrane was incubated with a polyclonal rabbit anti-GST antibody overnight at 4°C. After washing with TBS plus 0.1% Tween 20 (TBST), the PVDF membrane was incubated with mouse secondary antibody at room temperature for 1 hour. Far-Western blot binding was visualized on a Bio-Rad Chemidoc MP Western multifunctional imaging analyzer.

### GST-mAf1521 immunofluorescent double staining.

For immunocytochemistry, cells on slides were fixed with 4% paraformaldehyde for 20 minutes, permeabilized with 0.3% Triton X-100 for 30 minutes at room temperature, and incubated with GST-mAf1521 (6 ng/μL) in goat serum diluted in 1% BSA for 1 hour. The primary antibody (anti-GST, 1:1000; anti-GRP78/BiP, 1:100) diluted in 0.1% BSA was added dropwise to the slides, and left overnight at 4°C; the control group was replaced by PBS, placed at 4°C for overnight incubation. Secondary antibodies (1:200 in 0.1% BSA) — Cy3-labeled for anti-GST, FITC-labeled for anti-GRP78/BiP — were added and incubated at room temperature for 1 hour in the dark. Slides were mounted with DAPI-containing mounting medium. Finally, the samples were analyzed with a laser-scanning confocal fluorescence microscope (Nikon A1R).

### Cell growth and apoptosis assays.

For CCK8 analysis, briefly, the number of cells inoculated in each well of a 96-well plate was 1 × 10^4^, and at least 5 replicate wells were used in each group. When the cells grew to 50% confluence, the inhibitor MIBG was added at a concentration gradient of 0, 50, 100, 150, 200, 250, and 300 μmol/L. CCK8 reagent was added under dark conditions after 24 hours of MIBG treatment, and the OD value at 450 nm was detected with the Bio-Rad enzyme-labeled instrument 30 minutes later. Finally, the cell inhibition rate was calculated according to the following formula: inhibition rate (%) = ([Ac – As]/[Ac – Ab]) × 100. EdU cell proliferation analysis was performed with a BeyoClick EdU-594 proliferation kit according to the manufacturer’s protocol. Fluorescence images were visualized by Bio-Rad ZOE Fluorescent Cell Imager. For colony-forming assay, 500 cells were inoculated in each well of a 5-well plate, and treated with MIBG at a concentration of 20 μmol/mL for 15 days. Medium was changed every 2 days. Cells in the 6-well plate were fixed with 4% paraformaldehyde for 20 minutes, stained with Giemsa staining solution for 30 minutes, and dried at room temperature. A sheet of grid paper was superimposed under the 6-well plate for photographing, and the number of colonies formed within each grid was counted: colony formation rate = (number of clones/number of seeded cells) × 100%.

To measure apoptosis by Annexin V–FITC/PI double staining, Annexin V–FITC and PI staining was performed before sorting by flow cytometry. A TUNEL apoptosis assay was performed with a One-Step TUNEL cell apoptosis detection kit according to the manufacturer’s protocol (Beyotime Technologies). For Hoechst 33258 staining, cells were fixed with 4% paraformaldehyde for 20 minutes and 50 μL of Hoechst 33258 staining solution was added to each slide, incubated for 5 minutes, and covered with antifade mounting medium. Fluorescence images were visualized by Bio-Rad ZOE Fluorescent Cell Imager.

### RNA isolation and real-time PCR.

Total RNA in each group was extracted with Trizol reagent (Invitrogen) following the manufacturer’s protocol. For cDNA synthesis, 500–1000 ng total RNA was reverse transcribed using a PrimeScript 1st Strand cDNA Synthesis Kit (Takara). mRNA expression was analyzed using the SYBR Premix Ex Taq (Takara) on a 7500 system (Applied Biosystems) following the manufacturer’s recommendations.

The human-specific primers used for quantitative PCR are described in Table 9.

### Animals and xenografts.

Nude mice used in the experiment were purchased from HFK Bioscience. ART1 knockdown sequences were Forward: 5′-GCGAGUACAUCAAAGACAAdTd-3′, Reverse: 5′-UUGUCUUUGAUGUACUCGCdTd-3′. HanBio technology Co., Ltd embedded the sequence into the vector and packaged it into lentivirus, and the constructed vector is pCLenti-U6-shRNA (ART1)-CMV-mCherry-F2A-BSR-WPRE, characterized by red fluorescence and resistance to blasticidin (BSD). KRAS-WT, G12D, and G13D cells were infected with ART1 knockdown lentivirus, and empty lentivirus was used as control. Cells were filtrated with BSD at a concentration of 70 μg/mL according to the manufacturer’s recommendation. The cells were named KRAS WT-shART1, G12D-shART1, and G13D-shART1, respectively, and KRAS WT-NC, G12D-NC, and G13D-NC were used as controls. In MIBG treatment experiment, after the 15th day of subcutaneous tumor formation of KRAS G13D and KRAS WT HT-29 cells in nude mice, MIBG (PBS as a control) was intraperitoneally injected once every 3 days to observe the growth trend of the transplanted tumor. The final concentration of MIBG was 30 mg/kg. Five-week-old nude mice were injected subcutaneously with 5 × 10^6^ cells collected from each group, and tumor size and weight were followed for up to 4 weeks. The formula for calculating the tumor volume (*V*) is as follows: *V* = 0.52 × *L* × *W*^2^ (where *L* is the longest diameter of the tumor mass, *W* is the longest transverse diameter perpendicular to the long diameter). Tissues were stored at –80°C or fixed in 4% paraformaldehyde for later use.

### Co-IP assays.

In order to collect the protein lysate, 20–30 μL of IP protein lysate containing protease inhibitors was added to 1 × 10^5^ cells, mixed well, mixed on ice for 30 minutes, and then centrifuged at 4°C for 10 minutes (12,750*g*). Diluted anti-GRP78/BiP antibody (1:50) was added to the protein A–Sepharose suspension (MedChemExpress) and incubated in an inverting mixer (4°C, 2 hours). Then, cell lysate previously prepared was added and incubated in an inverting mixer (4°C, overnight) and after thorough washing, 1× SDS-PAGE loading buffer was used to denature and separate the magnetic beads, followed by SDS-PAGE detection.

### GRP78/BiP^R214K^ and GRP78/BiP^R470+492K^ plasmid transfection.

GRP78/BiP^R214K^ and GRP78/BiP^R470+492K^ overexpression plasmids were synthesized by OBiO Technology Co., Ltd. In GRP78/BiP^R214K^, R214 on the GRP78/BiP protein is mutated to lysine (K). In GRP78/BiP^R470/492K^, R470 and R492 on the GRP78/BiP protein are mutated to lysine (K). The empty vector is H2713-pcDNA3.1(+)-3xFLAG-P2A-EGFP, and the constructed vectors are pcDNA3.1(+)-HSPA5(R214K)-3xFLAG-P2A-EGFP and pcDNA3.1(+)-HSPA5(R470K&R492K)-3xFLAG-P2A-EGFP. Plasmid (4 μg) was mixed with 125 μL serum-free medium (tube A), and in a separate tube, 5 μL Lipofectamine 2000 was mixed with 125 μL serum-free medium (tube B), and both incubated at room temperature for 5 minutes. Tubes A and B were then combined to prepare a transfection complex, and incubated at room temperature for 10 minutes. The transfection complex was added to the adherent cells, gently agitated, and cells were collected 24 hours later.

### MALDI-TOF MS.

The experimental method of detecting LOVO and SW480 by MALDI-TOF MS has been described previously by our research group (50).

### Statistics.

In the survival analysis, the survival time was set at 150 months, and the log rank test was used for statistical analysis. IBM SPSS 22.0 software was used for statistical analysis of immunohistochemical results by the Wilcoxon rank-sum test and Mantel-Haenszel χ^2^ test. The Spearman correlation coefficient was calculated for correlation analysis. ART1 mRNA expression in patients with CRC was compared between KRAS-WT and KRAS-mutant groups using an unpaired 2-tailed *t* test, based on data acquired from the cBioPortal database. The gray values of all the bands in all Western blots, far-Western blots, and co-IP experiments were measured by ImageJ (v1.53a; NIH). Comparisons between groups were performed using the 2-tailed *t* test or 1-way ANOVA and Tukey’s HSD test. GraphPad Prism 8.0 software was used for data analysis and graphing. Independent unpaired 2-tailed *t* tests were performed in EdU, plate cloning, TUNEL, and Hoechst assays to compare the proliferation and apoptosis of cells in the DMSO and MIBG groups with different KRAS status. Statistical differences in tumor volume and weight between the groups were determined by unpaired Student’s *t* test or 1-way ANOVA and Tukey’s HSD test. All experiments were repeated more than 3 times, and a *P* value of less than 0.05 was considered significant.

### Ethics approval and consent to participate.

The proposed use of human participants in this study was reviewed and approved by the Ethics Committee of Chongqing Medical University, and all patients provided written informed consent. In addition, the Ethics Committee of the First Affiliated Hospital of Chongqing Medical University reviewed and approved this research proposal, which also conforms to the ethical principles of experimental animal research and the Declaration of Helsinki (2022-K510).

### Data availability statement.

The RNA sequencing data, mutation data, and clinical information of CRC samples were obtained from TCGA (https://www.cancer.gov/tcga). The data that support the findings of this study and raw data of MALDI-TOF MS are available on request from the corresponding author upon reasonable request. Values for all data points in graphs are reported in the Supporting Data Values file.

## Author contributions

SZ processed the data download, integration, and analysis, and developed methodology and wrote the manuscript. XC was responsible for the cell culture and RT-PCR experiments. QG and JH performed statistical analysis. YT and MX participated in judgment of the positive intensity of pathological sections. ML and QL provided technical support. YW provided funding support and was responsible for manuscript revision. All authors read and approved the final manuscript.

## Funding support

- Chongqing Natural Science Foundation Postdoctoral Project CSTB2022NSCQ-BHX0678.
- Innovation Project of Graduate Student in Chongqing, no. CYB19160.
- National Nature Science Foundation of China grant 30870946.

## Supplementary Material

Supplemental data

Unedited blot and gel images

Supporting data values

## Figures and Tables

**Table 7:**
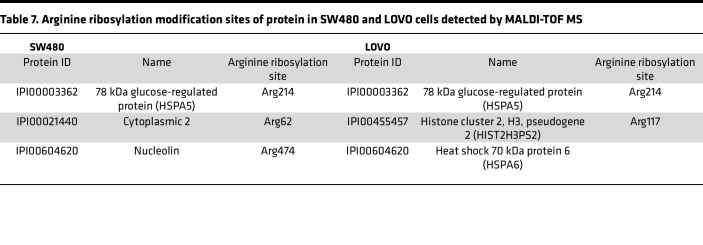
Arginine ribosylation modification sites of protein in SW480 and LOVO cells detected by MALDI-TOF MS

**Table 6:**
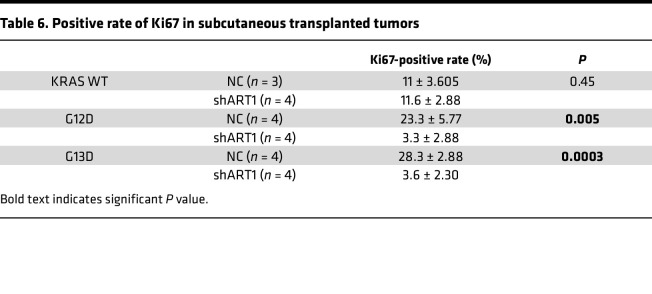
Positive rate of Ki67 in subcutaneous transplanted tumors

**Table 1:**
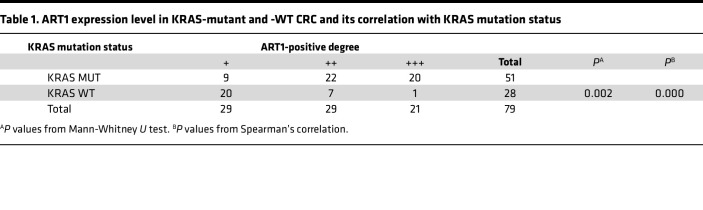
ART1 expression level in KRAS-mutant and -WT CRC and its correlation with KRAS mutation status

**Table 2:**
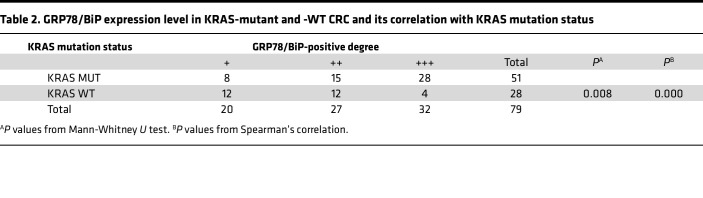
GRP78/BiP expression level in KRAS-mutant and -WT CRC and its correlation with KRAS mutation status

**Table 3:**
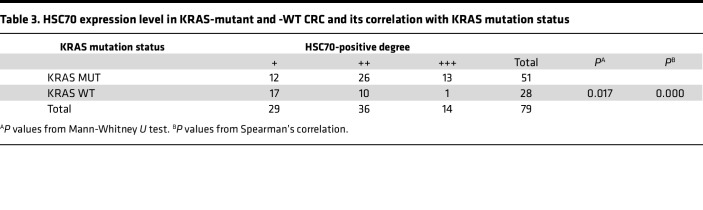
HSC70 expression level in KRAS-mutant and -WT CRC and its correlation with KRAS mutation status

**Table 4:**
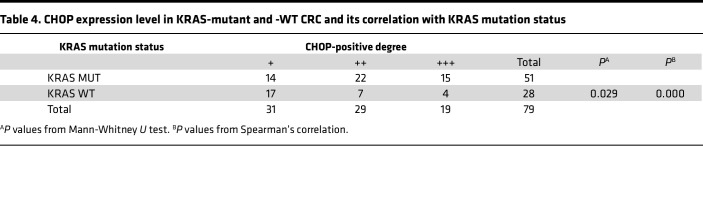
CHOP expression level in KRAS-mutant and -WT CRC and its correlation with KRAS mutation status

**Table 5:**
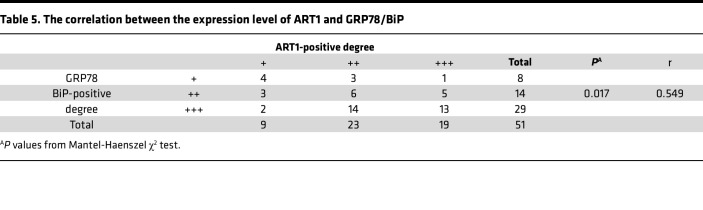
The correlation between the expression level of ART1 and GRP78/BiP

**Table 8:**
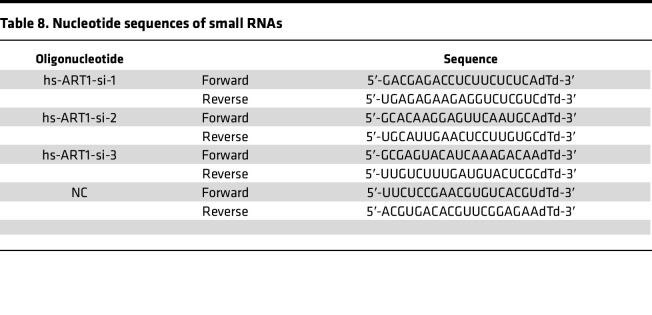
Nucleotide sequences of small RNAs

**Table 9:**
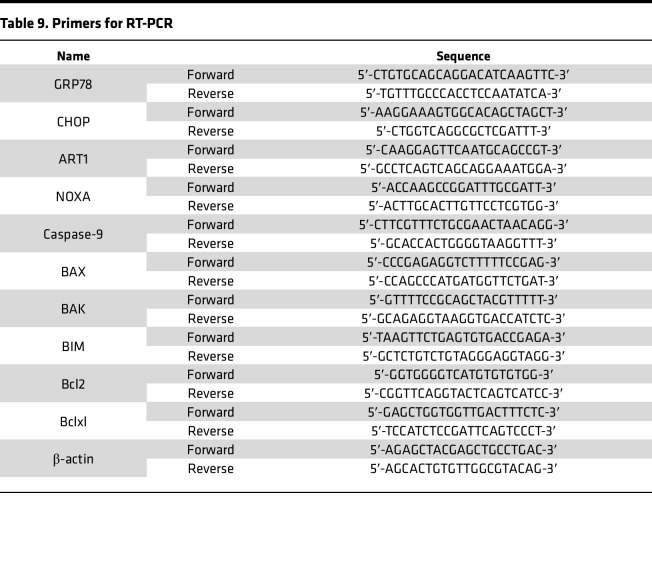
Primers for RT-PCR

## Version 1 01/23/2026

Electronic publication

## References

[1] Siegel RL (2022). Cancer statistics, 2022. CA Cancer J Clin.

[2] Nash GM (2010). KRAS mutation correlates with accelerated metastatic progression in patients with colorectal liver metastases. Ann Surg Oncol.

[3] Corcoran RB (2018). Combined BRAF, EGFR, and MEK inhibition in patients with BRAF^V600E^-mutant colorectal cancer. Cancer Discov.

[4] Pant S (2018). Clinical update on K-Ras targeted therapy in gastrointestinal cancers. Crit Rev Oncol Hematol.

[5] Rosty C (2013). Colorectal carcinomas with KRAS mutation are associated with distinctive morphological and molecular features. Mod Pathol.

[6] Benson AB (2021). Colon cancer, version 2.2021, NCCN clinical practice guidelines in oncology. J Natl Compr Canc Netw.

[7] Ebi H (2014). Not just gRASping at flaws: finding vulnerabilities to develop novel therapies for treating KRAS mutant cancers. Cancer Sci.

[8] Izar B (2013). The impact of EGFR mutation status on outcomes in patients with resected stage I non-small cell lung cancers. Ann Thorac Surg.

[9] Wagner S (2019). Suppression of interferon gene expression overcomes resistance to MEK inhibition in KRAS-mutant colorectal cancer. Oncogene.

[10] Kawaguchi S, Ng DTW (2011). Cell biology. Sensing ER stress. Science.

[11] Mokarram P (2017). New frontiers in the treatment of colorectal cancer: autophagy and the unfolded protein response as promising targets. Autophagy.

[12] De Raedt T (2011). Exploiting cancer cell vulnerabilities to develop a combination therapy for Ras-driven tumors. Cancer Cell.

[13] Denoyelle C (2006). Anti-oncogenic role of the endoplasmic reticulum differentially activated by mutations in the MAPK pathway. Nat Cell Biol.

[14] Yang L (2016). Arginine ADP-ribosyltransferase 1 promotes angiogenesis in colorectal cancer via the PI3K/Akt pathway. Int J Mol Med.

[15] Xu JX (2017). Effect of ART1 on the proliferation and migration of mouse colon carcinoma CT26 cells in vivo. Mol Med Rep.

[16] Xiao M (2013). ART1 silencing enhances apoptosis of mouse CT26 cells via the PI3K/Akt/NF-κB pathway. Cell Physiol Biochem.

[17] Butepage M (2015). Intracellular mono-ADP-ribosylation in signaling and disease. Cells.

[18] Verheugd P (2016). Players in ADP-ribosylation: readers and erasers. Curr Protein Pept Sci.

[19] Chambers JE (2012). ADP ribosylation adapts an ER chaperone response to short-term fluctuations in unfolded protein load. J Cell Biol.

[20] Ledford BE, Leno GH (1994). ADP-ribosylation of the molecular chaperone GRP78/BiP. Mol Cell Biochem.

[21] Fabrizio G (2015). ARTC1-mediated ADP-ribosylation of GRP78/BiP: a new player in endoplasmic-reticulum stress responses. Cell Mol Life Sci.

[22] Hetz C (2012). The unfolded protein response: controlling cell fate decisions under ER stress and beyond. Nat Rev Mol Cell Biol.

[23] Hetz C (2020). Mechanisms, regulation and functions of the unfolded protein response. Nat Rev Mol Cell Biol.

[24] Poltronieri P (2021). Mono(ADP-ribosyl)ation enzymes and NAD^+^ metabolism: a focus on diseases and therapeutic perspectives. Cells.

[25] Fakih MG (2015). Metastatic colorectal cancer: current state and future directions. J Clin Oncol.

[26] Fearon ER (2011). Molecular genetics of colorectal cancer. Annu Rev Pathol.

[27] Dumartin L (2017). ER stress protein AGR2 precedes and is involved in the regulation of pancreatic cancer initiation. Oncogene.

[28] Dokladny K (2015). Heat shock response and autophagy--cooperation and control. Autophagy.

[29] Malhi H, Kaufman RJ (2011). Endoplasmic reticulum stress in liver disease. J Hepatol.

[30] Ramirez MU (2019). Endoplasmic reticulum stress pathway, the unfolded protein response, modulates immune function in the tumor microenvironment to impact tumor progression and therapeutic response. Int J Mol Sci.

[31] Lu G (2020). Targeting the GRP78 pathway for cancer therapy. Front Med (Lausanne).

[32] Shen J (2017). GRP78 haploinsufficiency suppresses acinar-to-ductal metaplasia, signaling, and mutant *Kras*-driven pancreatic tumorigenesis in mice. Proc Natl Acad Sci U S A.

[33] Rao RV (2002). Coupling endoplasmic reticulum stress to the cell death program: role of the ER chaperone GRP78. FEBS Lett.

[34] Melber A, Haynes CM (2018). UPR^mt^ regulation and output: a stress response mediated by mitochondrial-nuclear communication. Cell Res.

[35] Antonucci L (2015). Basal autophagy maintains pancreatic acinar cell homeostasis and protein synthesis and prevents ER stress. Proc Natl Acad Sci U S A.

[36] Yang Q (2017). Cuprous oxide nanoparticles trigger ER stress-induced apoptosis by regulating copper trafficking and overcoming resistance to sunitinib therapy in renal cancer. Biomaterials.

[37] Chen X, Cubillos-Ruiz JR (2021). Endoplasmic reticulum stress signals in the tumour and its microenvironment. Nat Rev Cancer.

[38] Song M, Cubillos-Ruiz JR (2019). Endoplasmic reticulum stress responses in intratumoral immune cells: implications for cancer immunotherapy. Trends Immunol.

[39] Dani N (2009). Combining affinity purification by ADP-ribose-binding macro domains with mass spectrometry to define the mammalian ADP-ribosyl proteome. Proc Natl Acad Sci U S A.

[40] Kustatscher G (2005). Splicing regulates NAD metabolite binding to histone macroH2A. Nat Struct Mol Biol.

[41] Gifford JB, Hill R (2018). GRP78 influences chemoresistance and prognosis in cancer. Curr Drug Targets.

[42] Lee AS (2007). GRP78 induction in cancer: therapeutic and prognostic implications. Cancer Res.

[43] Madhavan S, Nagarajan S (2020). GRP78 and next generation cancer hallmarks: an underexplored molecular target in cancer chemoprevention research. Biochimie.

[44] Hetz C, Papa FR (2018). The unfolded protein response and cell fate control. Mol Cell.

[45] Chang TK (2018). Coordination between two branches of the unfolded protein response determines apoptotic cell fate. Mol Cell.

[46] Rozpedek W (2016). The role of the PERK/eIF2α/ATF4/CHOP signaling pathway in tumor progression during endoplasmic reticulum stress. Curr Mol Med.

[47] Hughes D, Mallucci GR (2019). The unfolded protein response in neurodegenerative disorders - therapeutic modulation of the PERK pathway. FEBS J.

[48] Fromowitz FB (1987). Ras p21 expression in the progression of breast cancer. Hum Pathol.

[49] Nowak K (2020). Engineering Af1521 improves ADP-ribose binding and identification of ADP-ribosylated proteins. Nat Commun.

[50] Ling F (2017). Mono-ADP-ribosylation of histone 3 at arginine-117 promotes proliferation through its interaction with P300. Oncotarget.

