# Supplementary material for: The critical role of GRP78/BiP MARylation in ER stress of KRAS-mutant colorectal cancer
Source: JCI Insight. 2026 Jan 23;11(2):e182809. doi: 10.1172/jci.insight.182809 (PMC12892896; doi:10.1172/jci.insight.182809)
Supplement: Supplemental data [file jciinsight-11-182809-s187.pdf]

Supplemental Figure.1

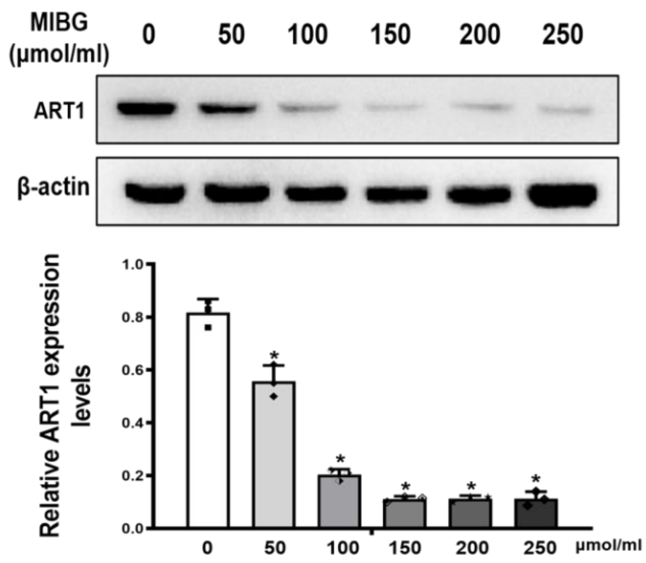

Figure.S1. Effects of concentration gradient of ART1 inhibitor MIBG on ART1 expression in HT-29 cell lines

Supplemental Figure.2

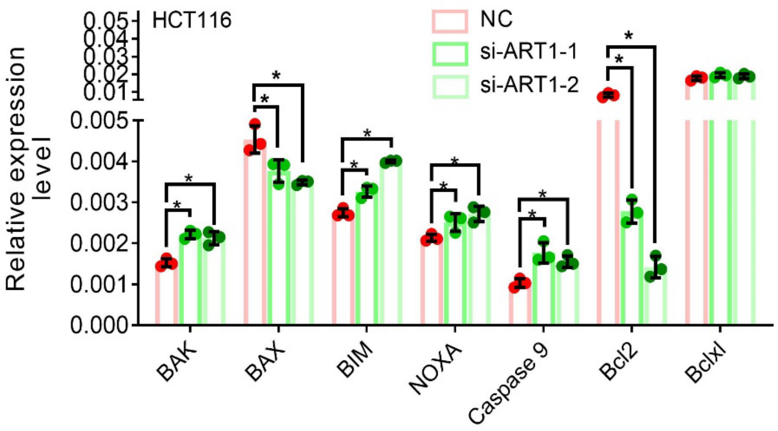

Figure.S2. The mRNA levels of Bcl2, Bclxl and apoptosis-related genes Bax, Bak, Bim, caspase9 and NOXA in HCT116 cells with knockdown of ART1, *p<0.01, mean ± SEM, n=3.

Supplemental Figure.3

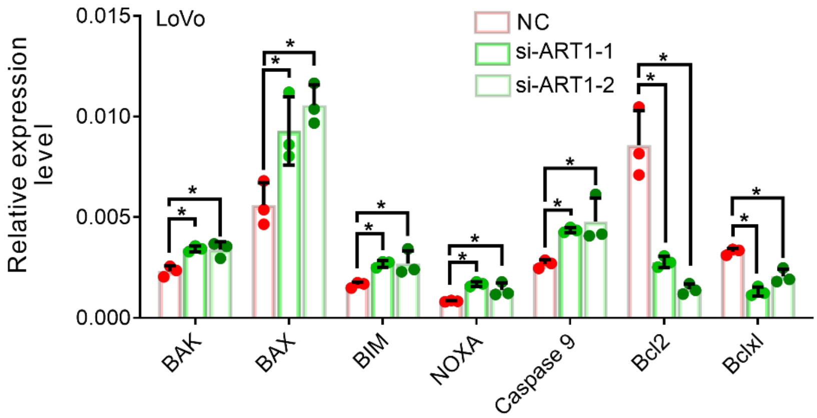

Figure.S3. The mRNA levels of Bcl2, Bclxl and apoptosis-related genes Bax, Bak, Bim, caspase9 and NOXA in LoVo cells with knockdown of ART1, *p<0.01, mean ± SEM, n=3.

Supplemental Figure.S4

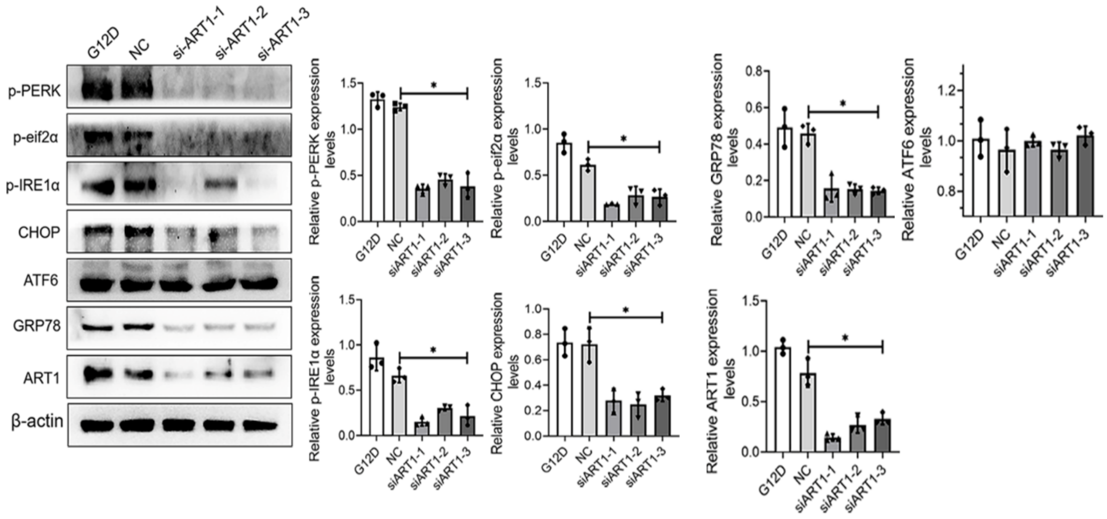

Figure.S4. The effect of siART1 on the key proteins in the UPR signaling pathway in G12D (H) and G13D (I)cells, *p<0.01, mean ± SEM, n=3.

Supplemental Figure.5

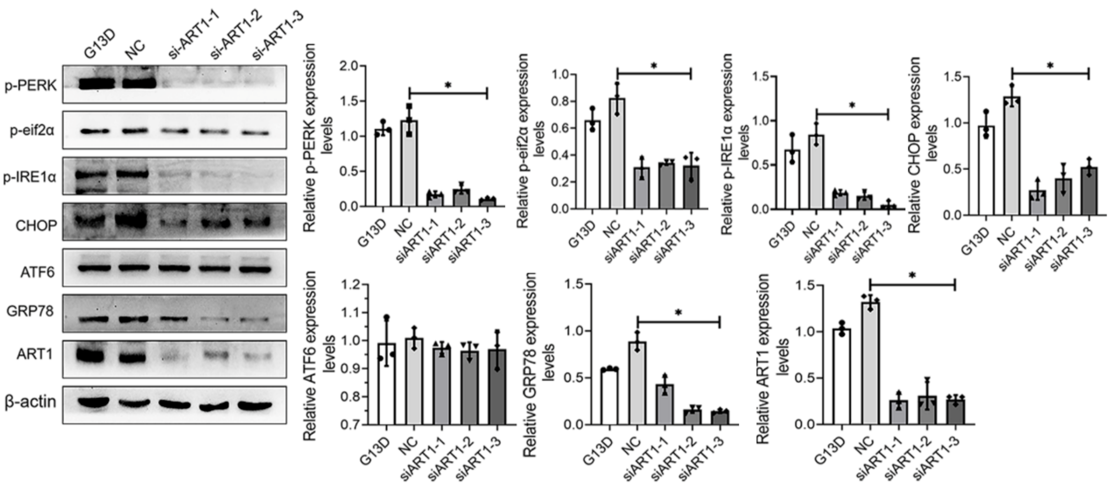

Figure.S5. The effect of siART1 on the key proteins in the UPR signaling pathway in G13D cells, *p<0.01, mean ± SEM, n=3.
