# Supplementary material for: The critical role of GRP78/BiP MARylation in ER stress of KRAS-mutant colorectal cancer
Source: JCI Insight. 2026 Jan 23;11(2):e182809. doi: 10.1172/jci.insight.182809 (PMC12892896; doi:10.1172/jci.insight.182809)
Supplement: Unedited blot and gel images [file jciinsight-11-182809-s188.pdf]

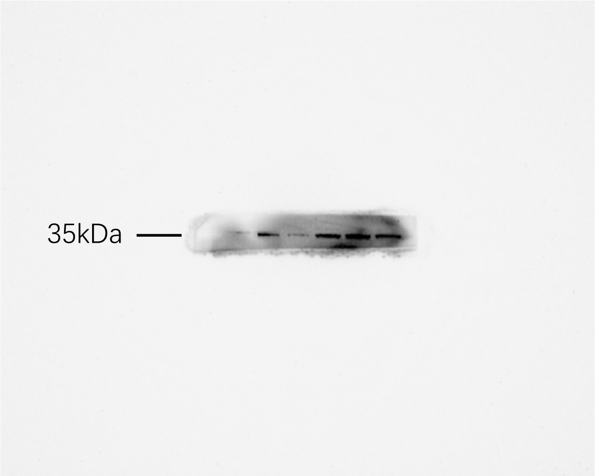

FIGURE-1K-ART1(37kDa)

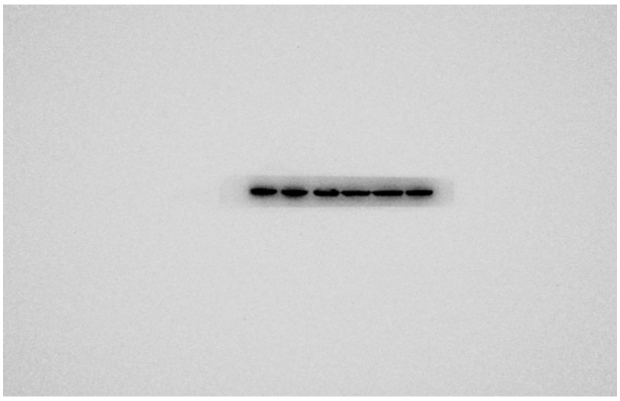

FIGURE1K-β-actin

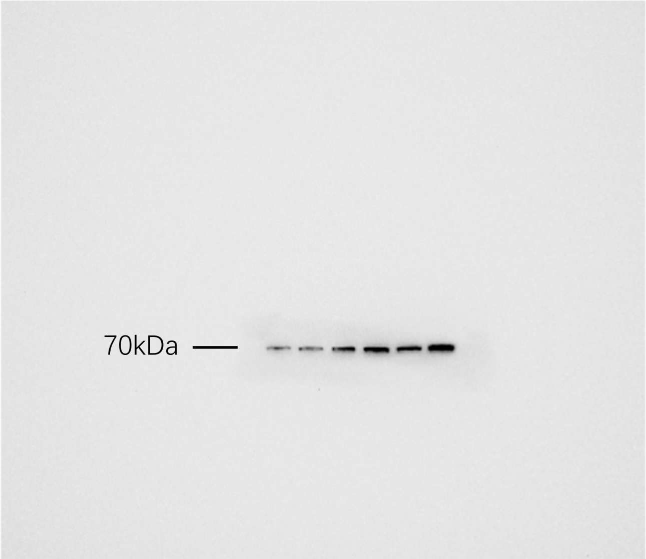

FIGURE1O-HSC70 (70kDa)

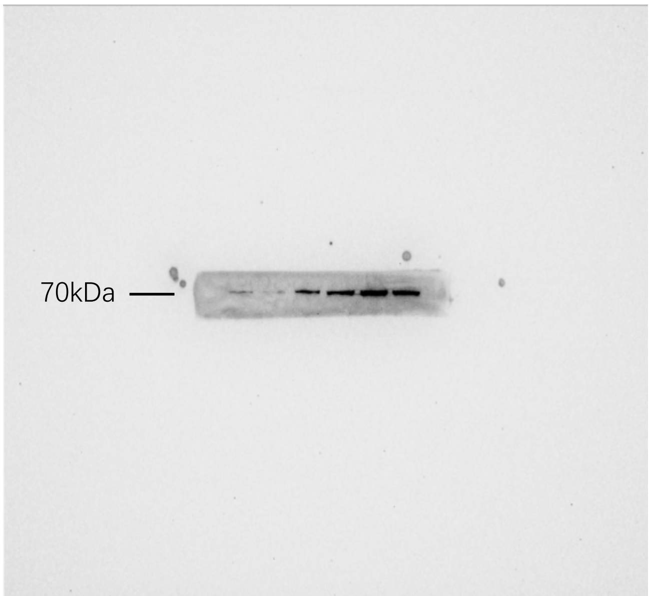

FIGURE1O-GRP78(78kDa)

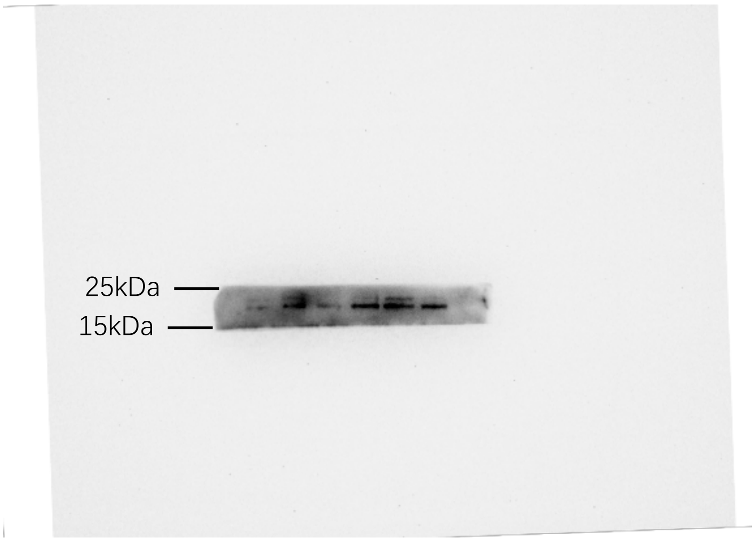

FIGURE1O-CHOP（19kDa）

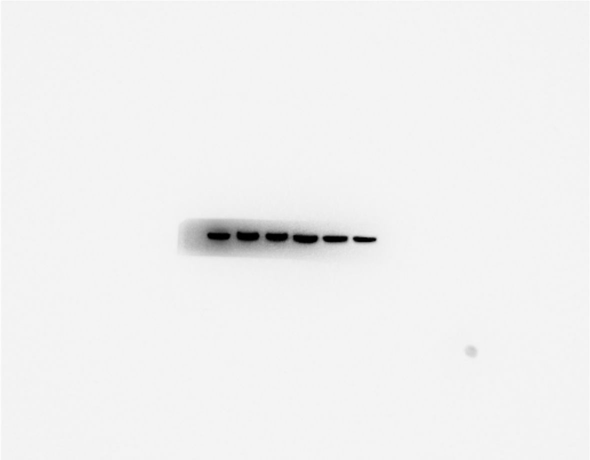
FIGURE1O-β-actin

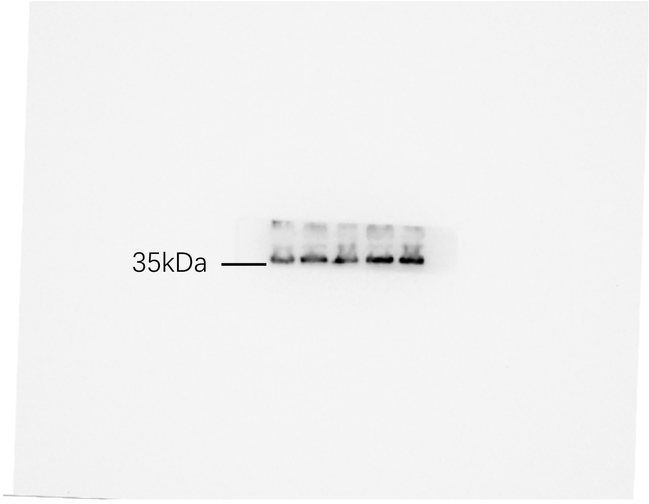

FIGURE2B-ART1（37kDa）

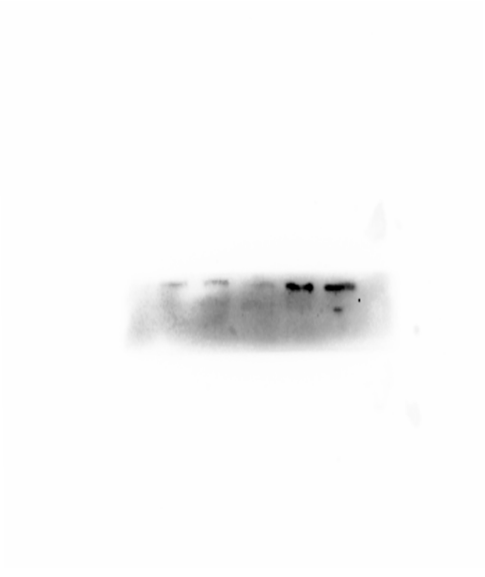

FIGURE2B-CHOP

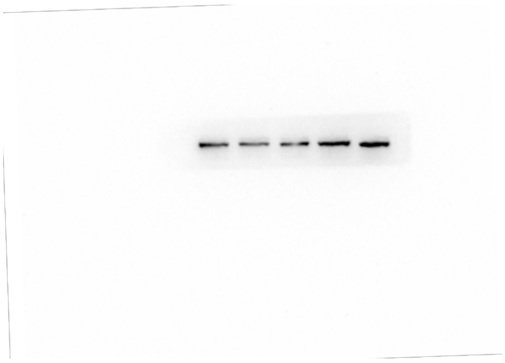

FIGURE2B-GRP78

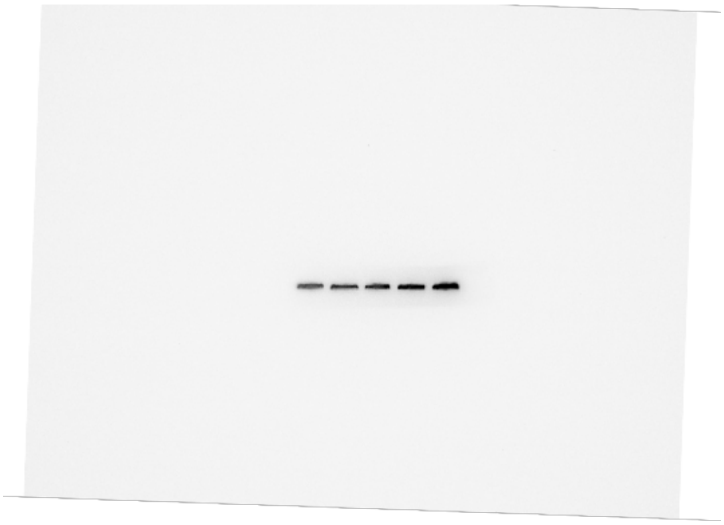

FIGURE2B-HSC70

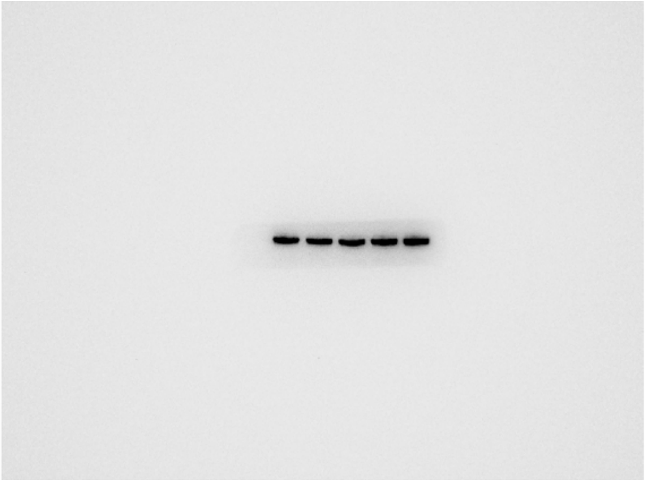

FIGURE2B-β-actin

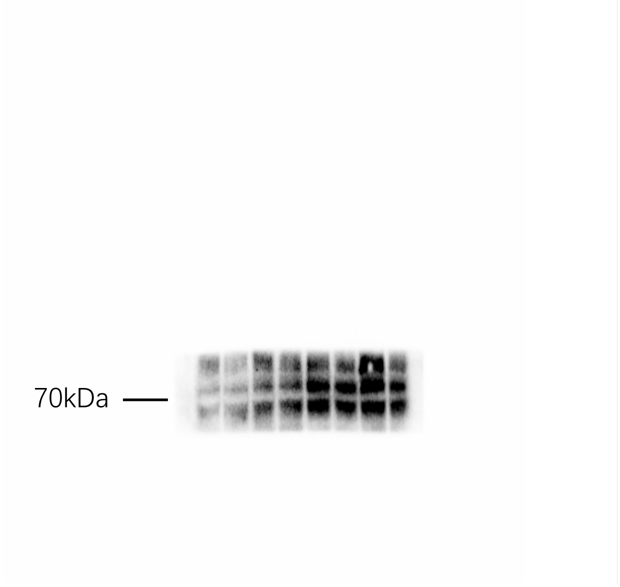

FIGURE2D-AMF1521

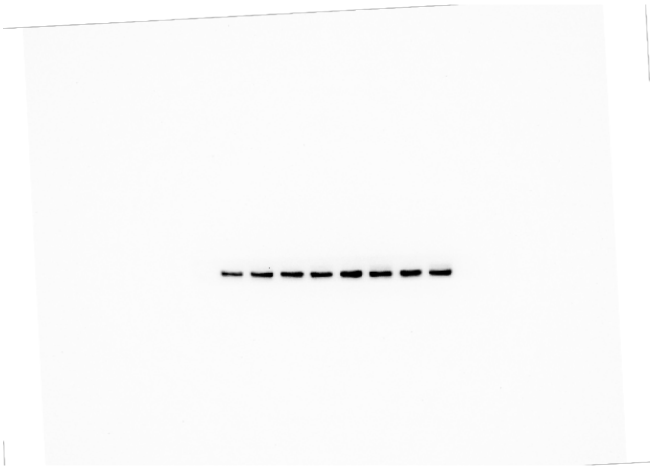

FIGURE2D-GRP78

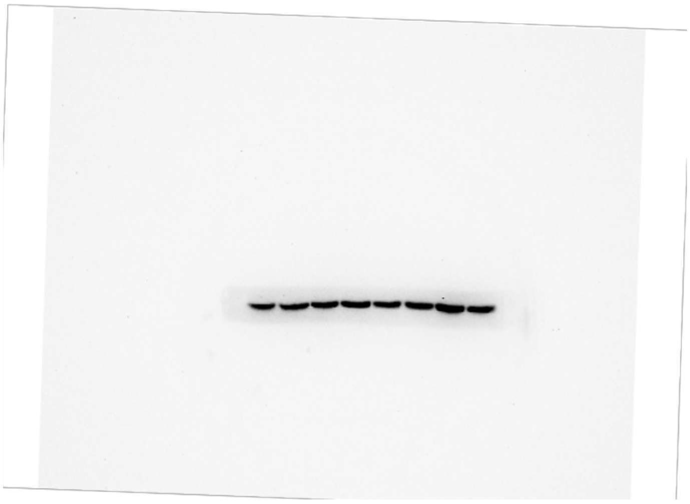

FIGURE2D-β-actin

FIGURE5F-ART1

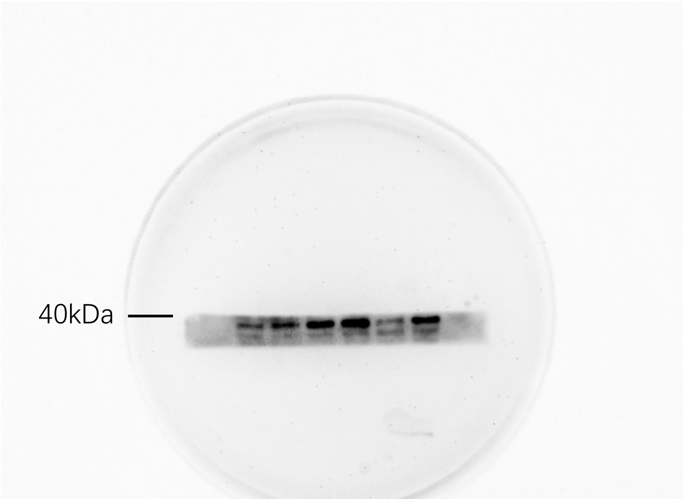

FIGURE5F-CASPASE9(46kDa)

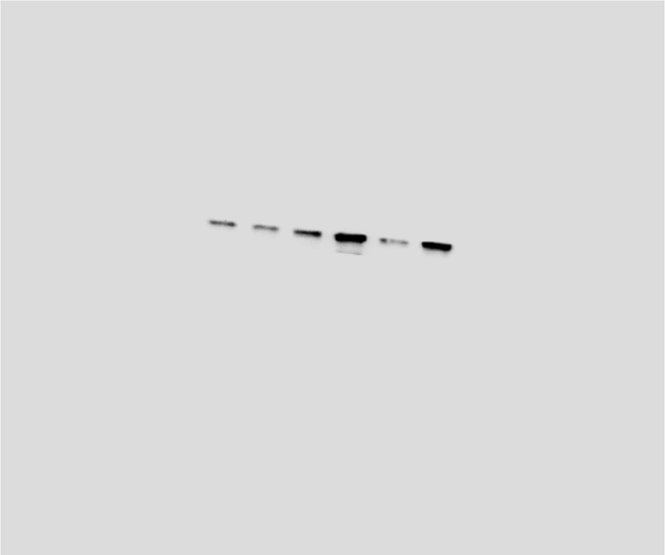

FIGURE5F-CLEAVED-CASPASE3

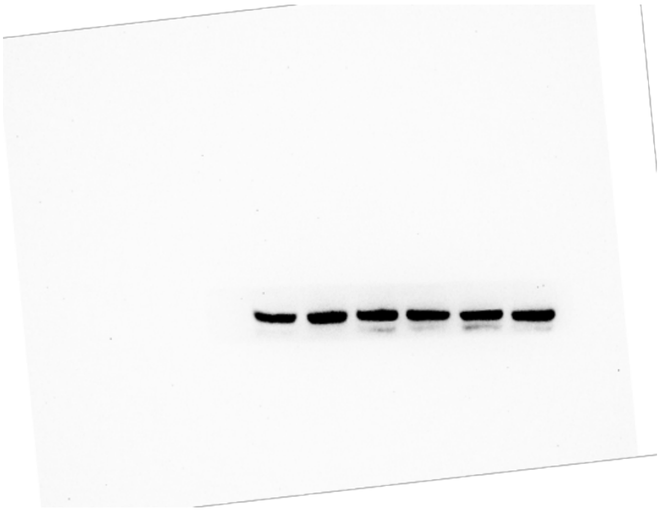

FIGURE5F-β-actin

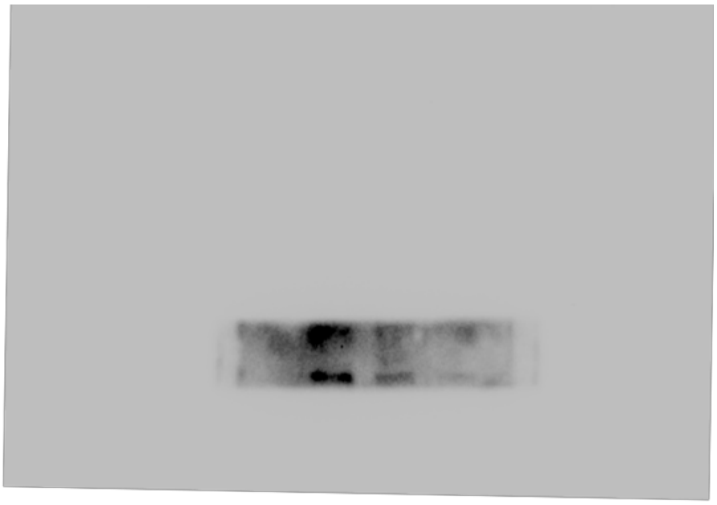

FIGURE5G-CACO2-ART1

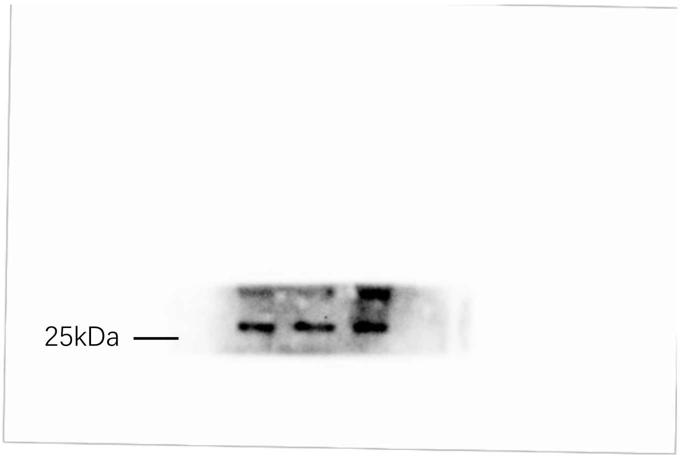

FIGURE5G-CACO2-BCL2(26kDa)

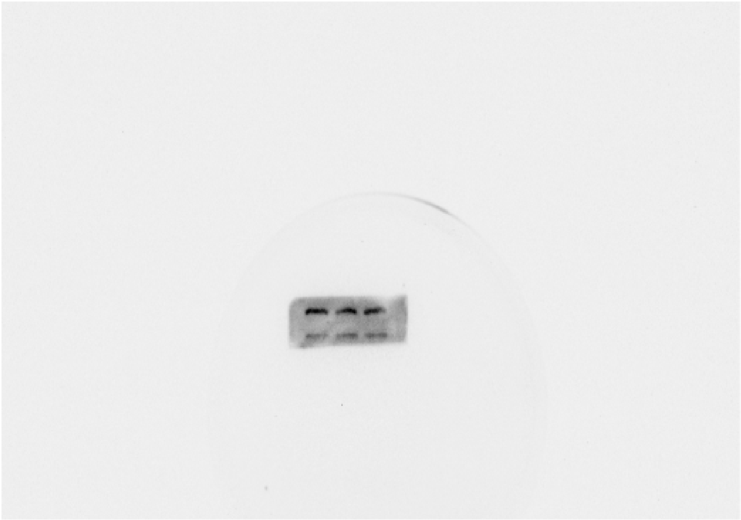

FIGURE5G-CACO2-CLEAVED-CASPASE3

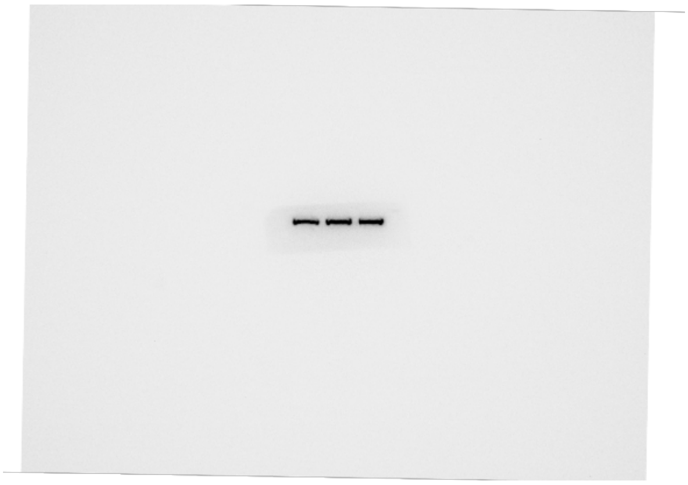

FIGURE5G-CACO2-GRP78

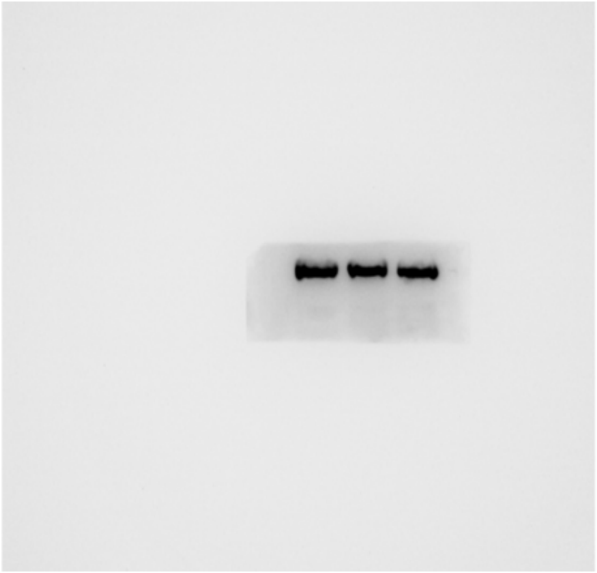

FIGURE5G-CACO2-β-actin

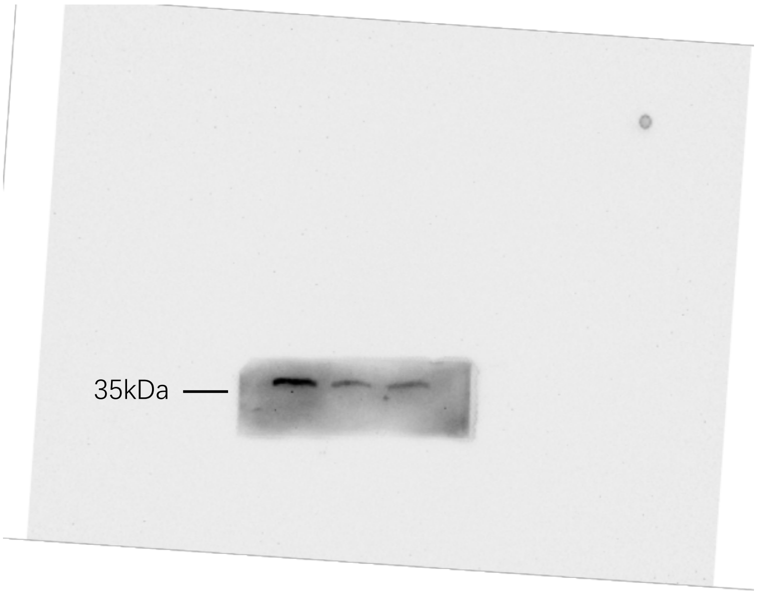

FIGURE5G-HCT116-ART1

(37kDa)

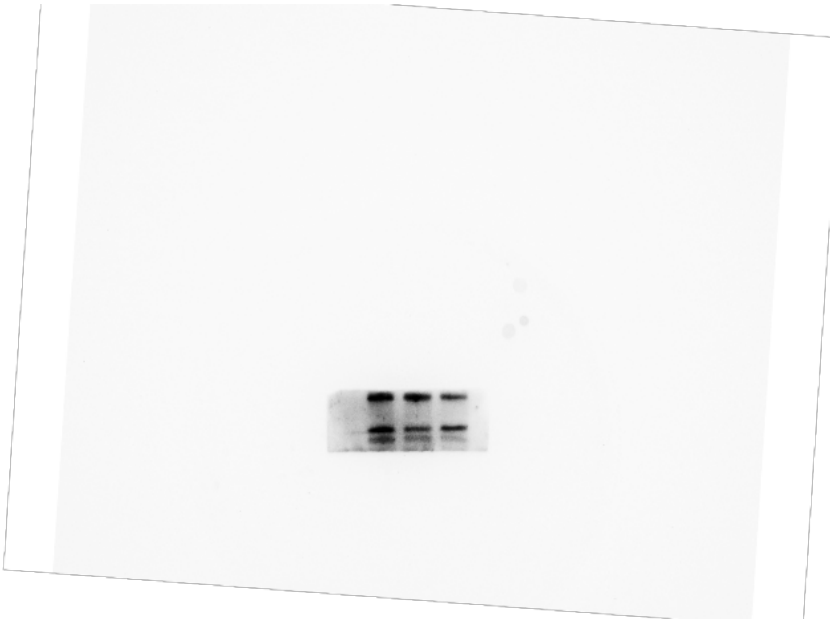

FIGURE5G-HCT116-

BCL2

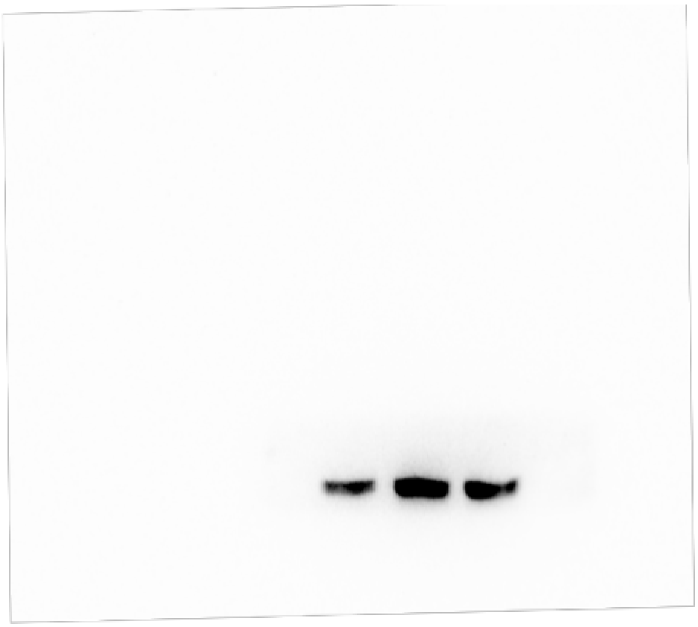

FIGURE5G-HCT116-

CLEAVED-CASPASE3

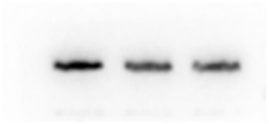

FIGURE5G-HCT116-GRP78

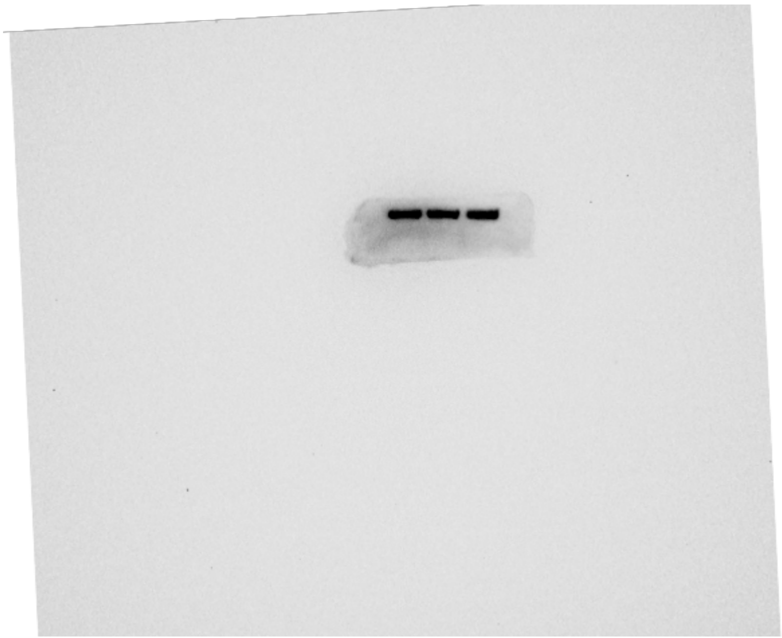

FIGURE5G-HCT116-β-actin

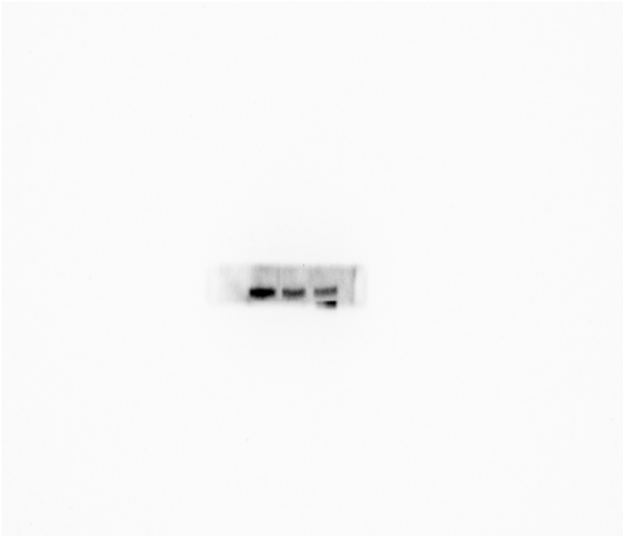

FIGURE5G-HT-29-ART1

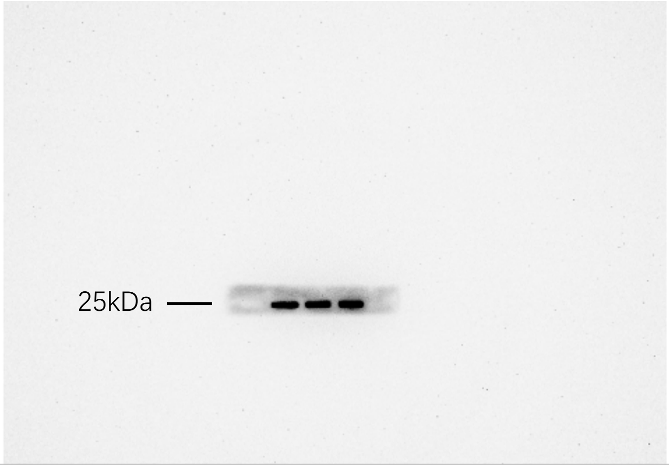

FIGURE5G-HT-29-BCL2

(26kDa)

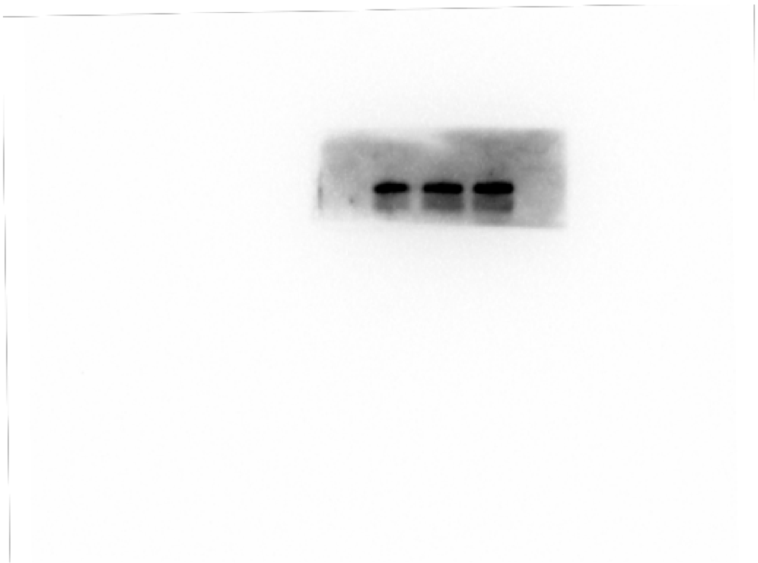

FIGURE5G-HT-29-

CLEAVED-CASPASE3

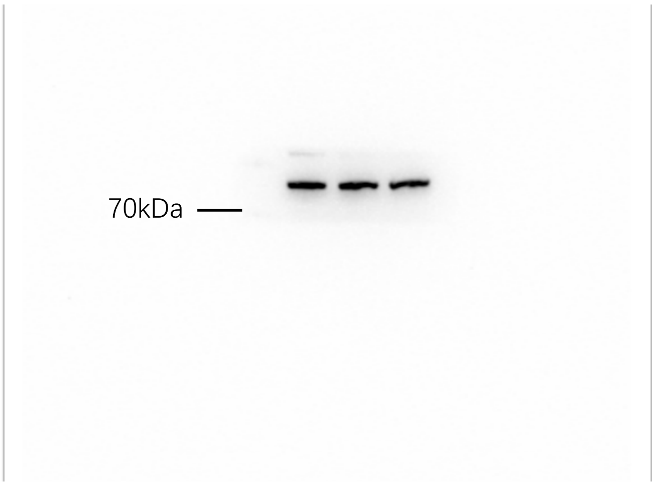

FIGURE5G-HT-29-GRP78(70kDa)

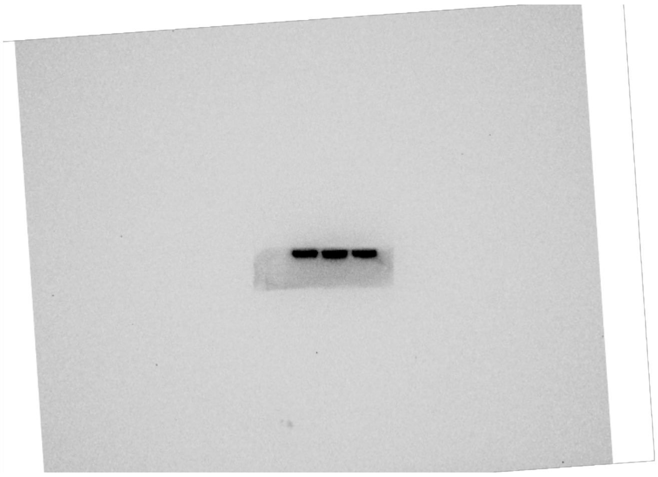
FIGURE5G-HT-29-β-actin

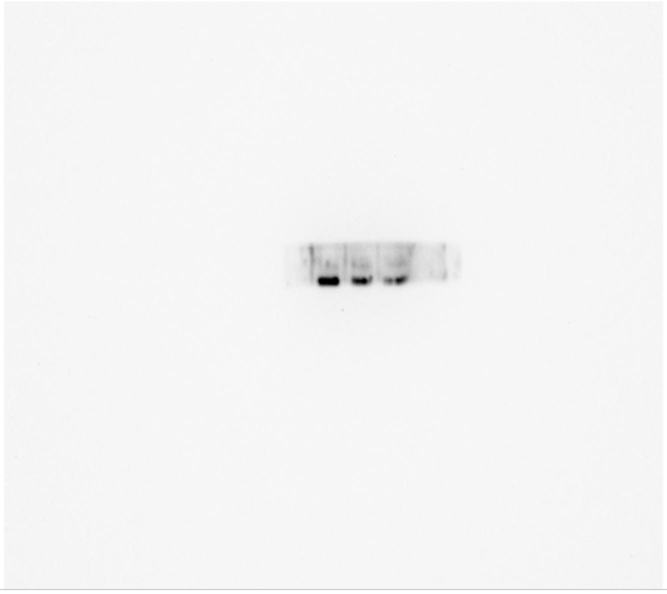
FIGURE5G-LOVO-ART1

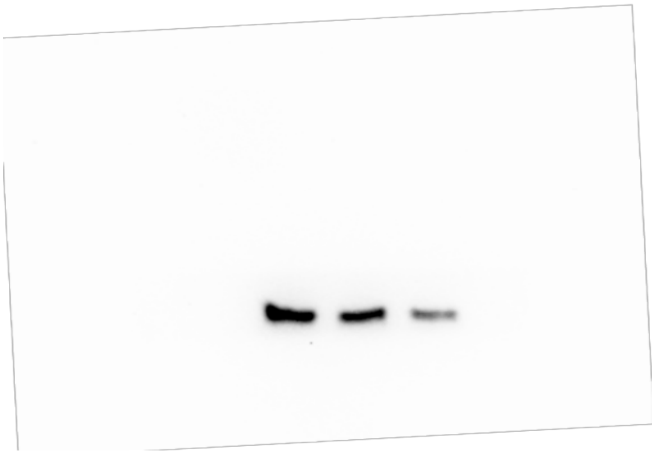

FIGURE5G-LOVO-BCL2

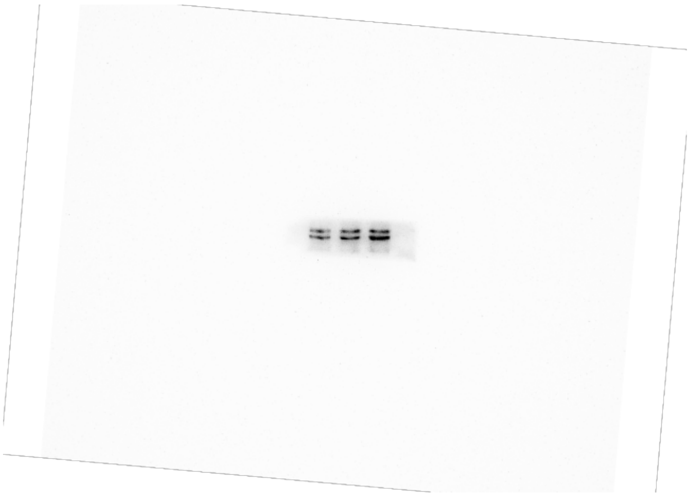

FIGURE5G-LOVO-CLEAVED-CASPASE3

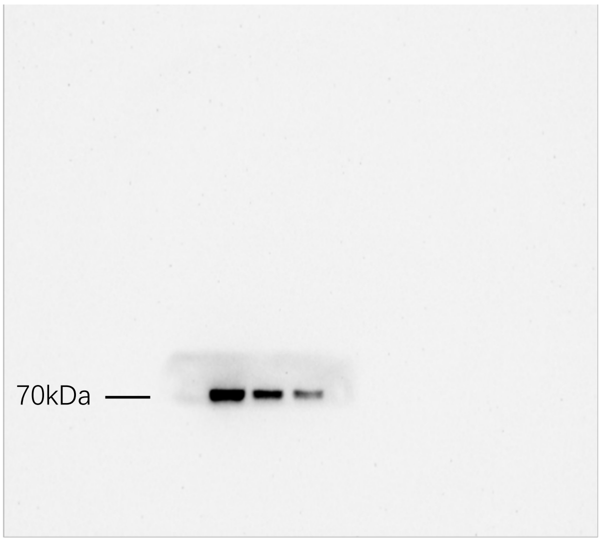

FIGURE5G-LOVO-GRP78(78kDa)

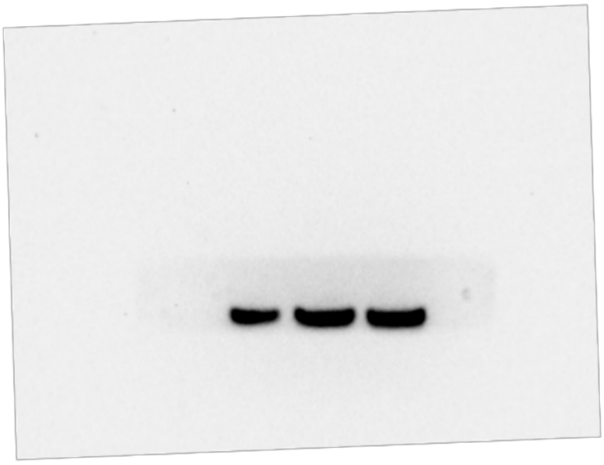

FIGURE5G-LOVO-β-actin

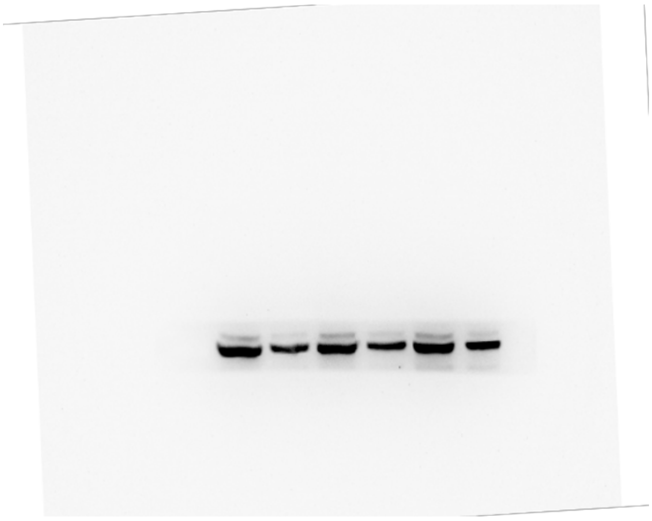

FIGURE5H-ART1

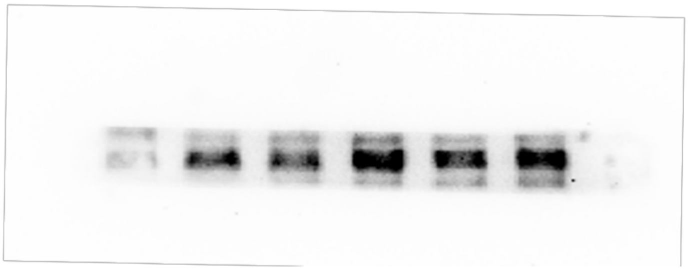

FIGURE5H-CLEAVED-CASPASE3

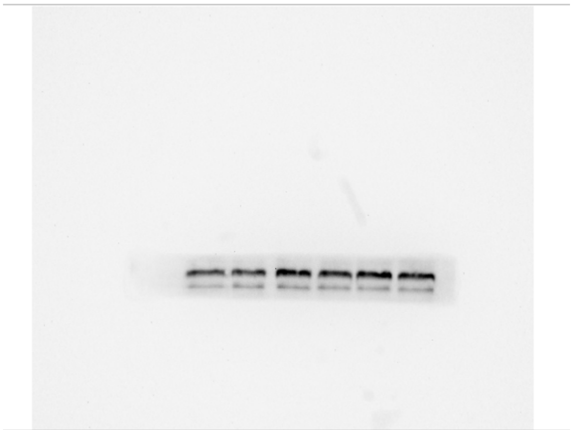

FIGURE5H-GRP78

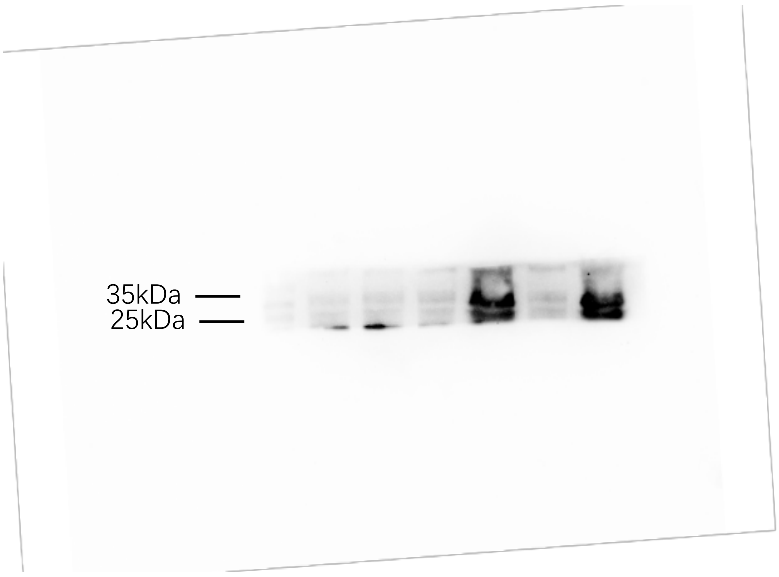

FIGURE5H-CLEAVED-PARP(26kDa)

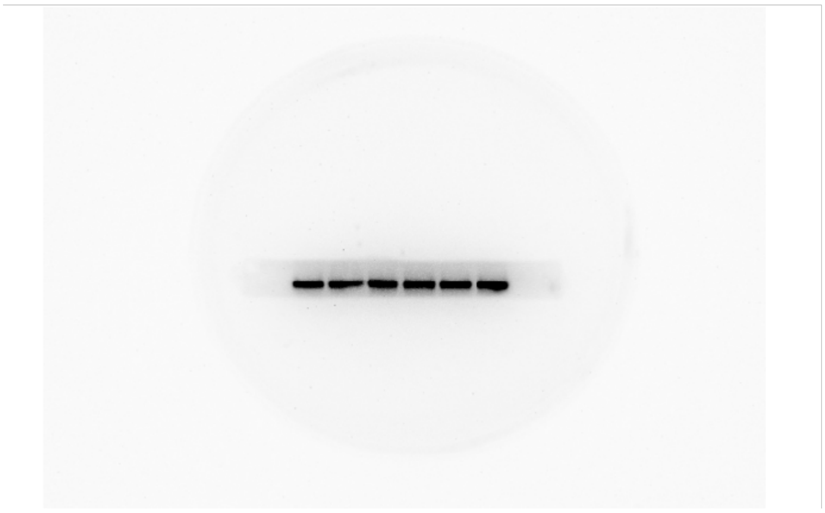

FIGURE5H-β-actin

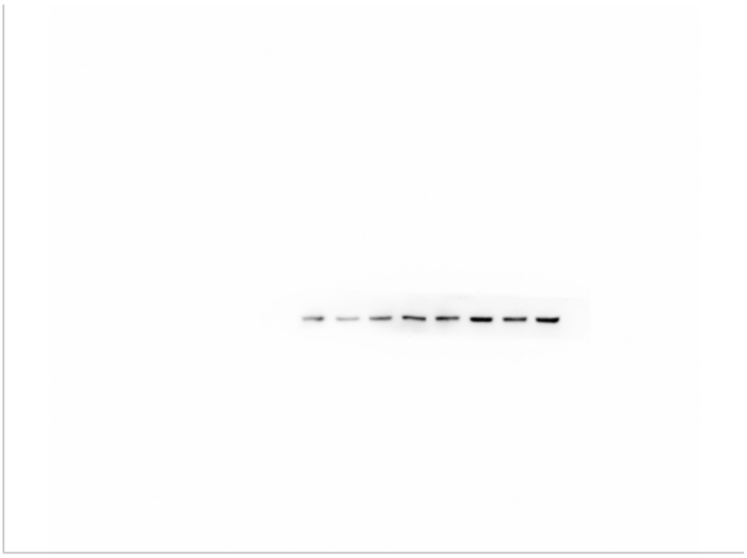

FIGURE6C-12H-GRP78

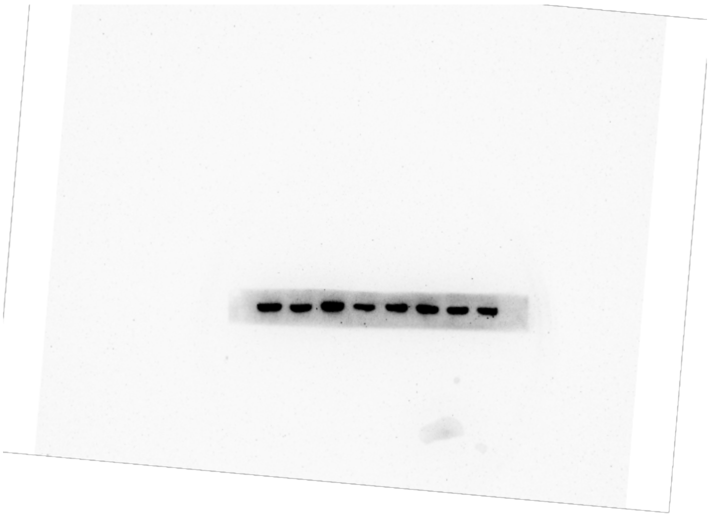

FIGURE6C-12H-β-actin

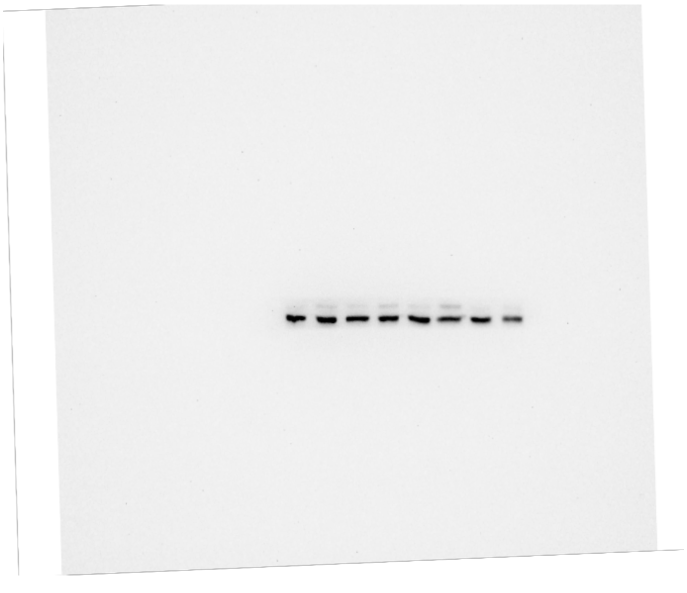

FIGURE6C-24H-GRP78

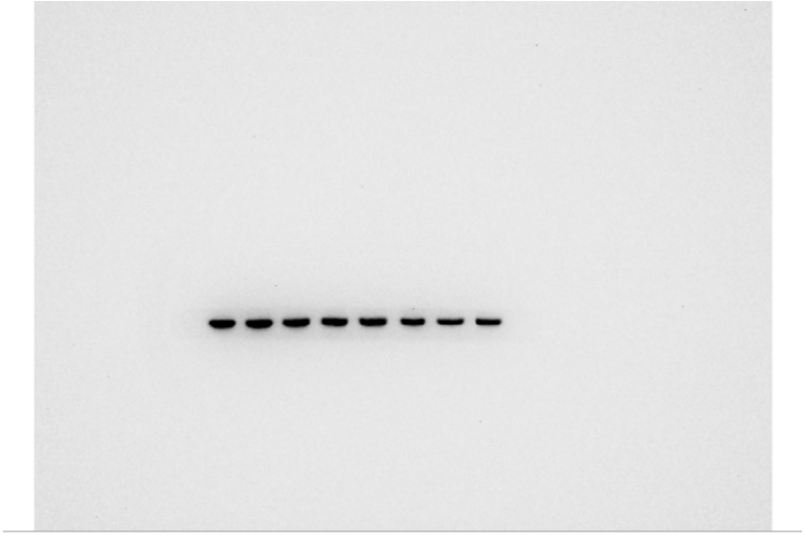

FIGURE6C-24H-β-actin

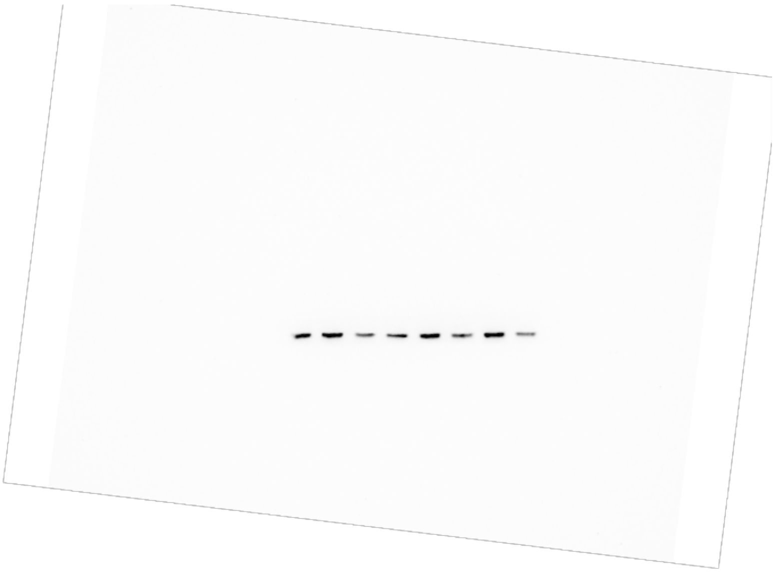

FIGURE6C-36H-GRP78

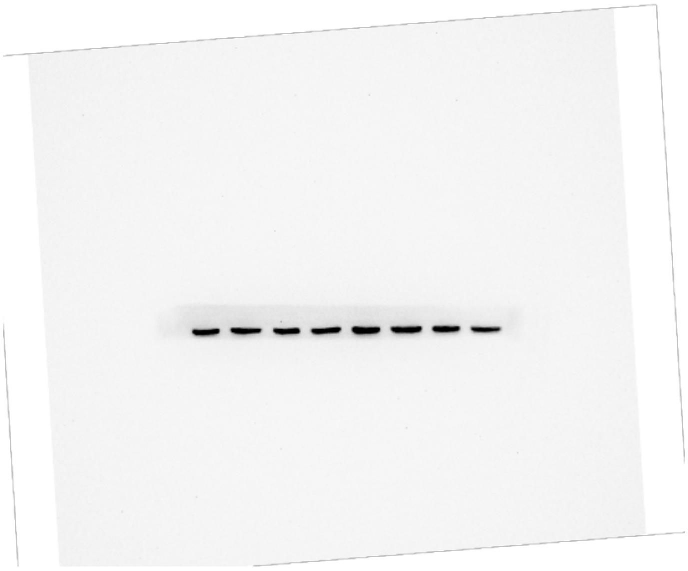

FIGURE6C-36H-β-actin

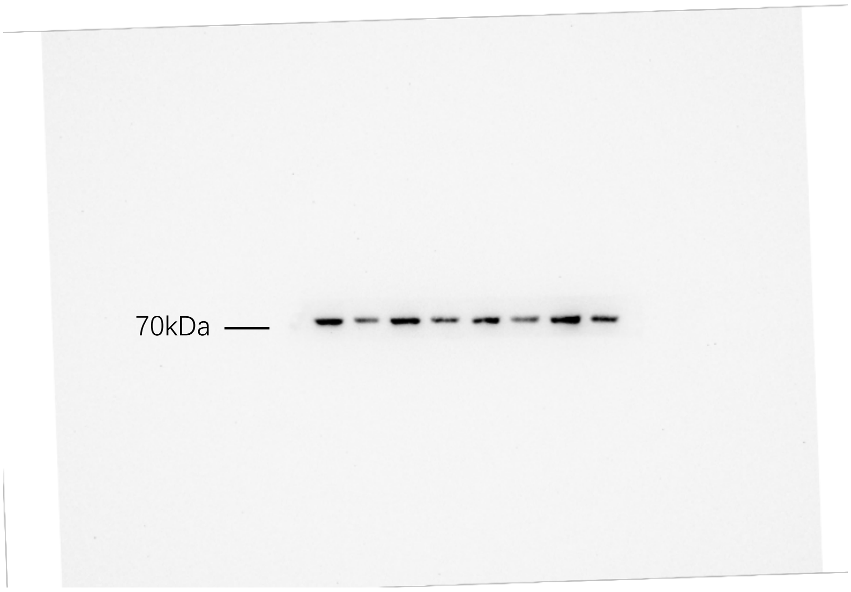

FIGURE6C-48H-GRP78(78kDa)

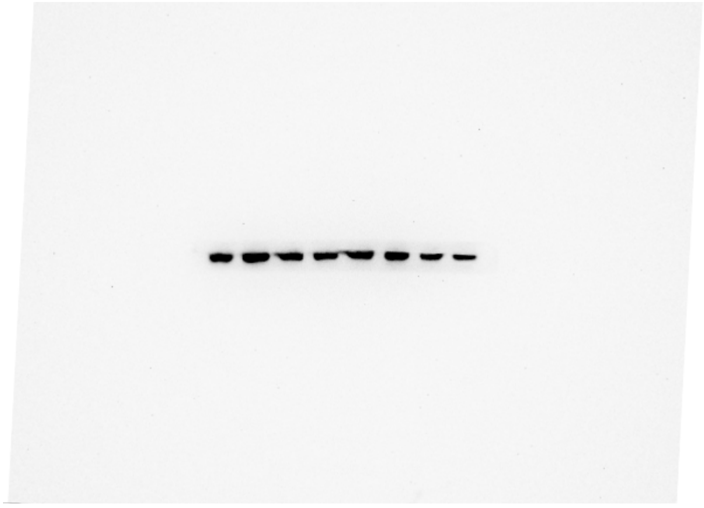

FIGURE6C-48H-β-actin

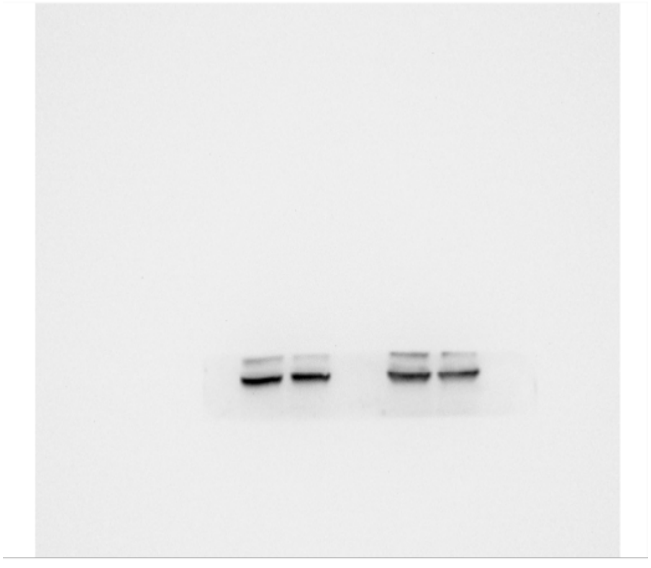

FIGURE6D-IB-ATF6

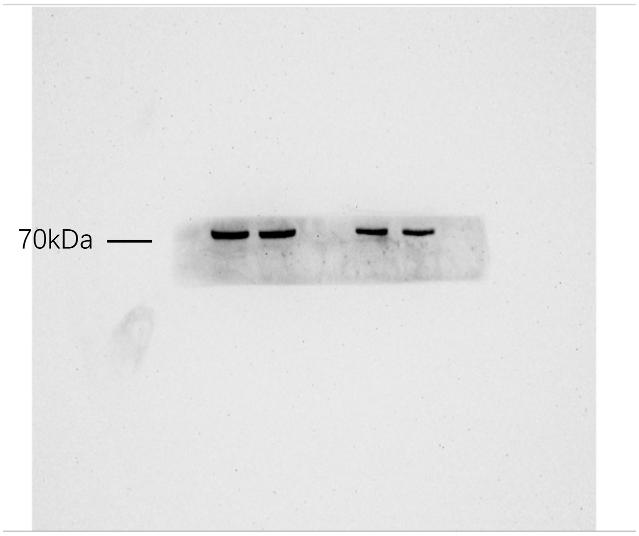

FIGURE6D-IB-GRP78(78kDa)

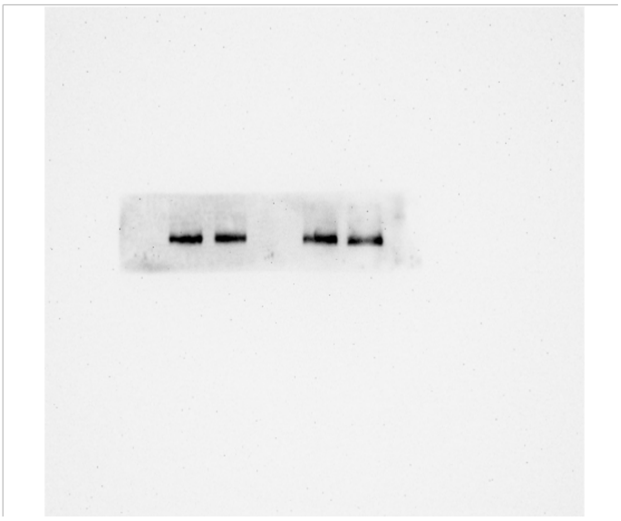

FIGURE6D-IB-IRE1

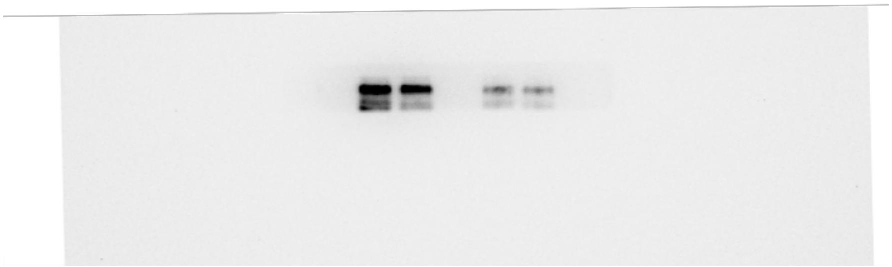

FIGURE6D-IB-PERK

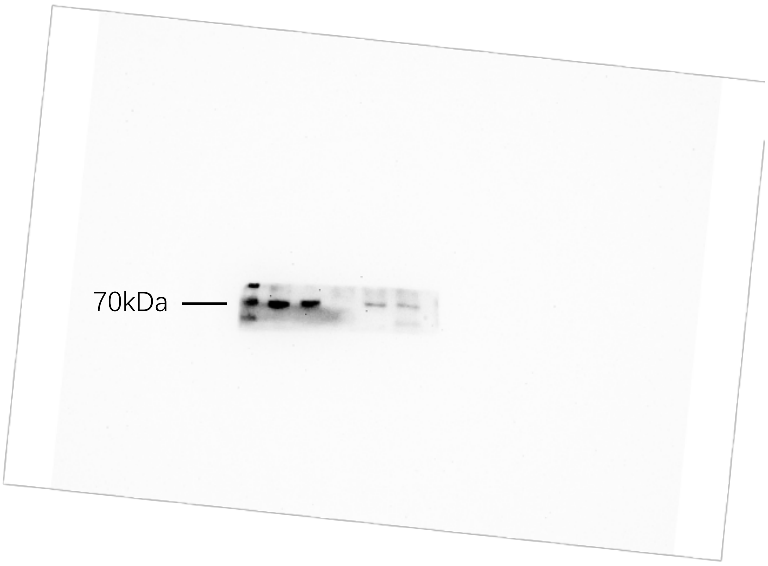

FIGURE6E-IB-ATF6(75kDa)

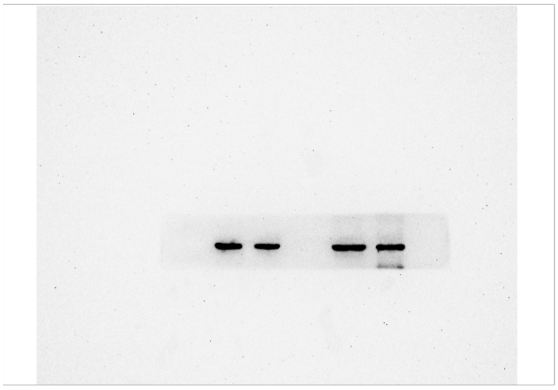

FIGURE6E-IB-GRP78

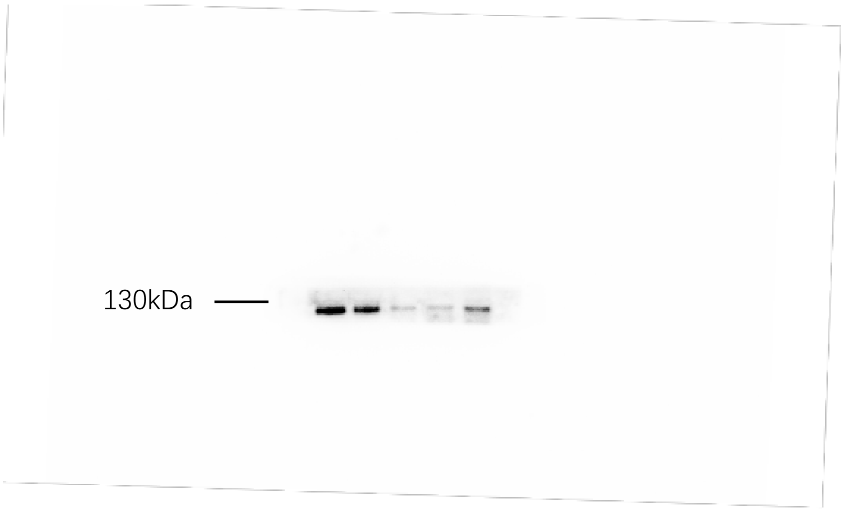

FIGURE6E-IB-IRE1(110kDa)

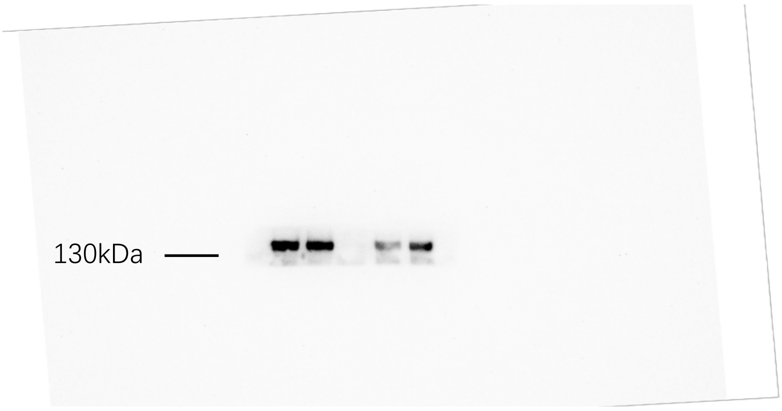

FIGURE6E-IB-PERK(140kDa)

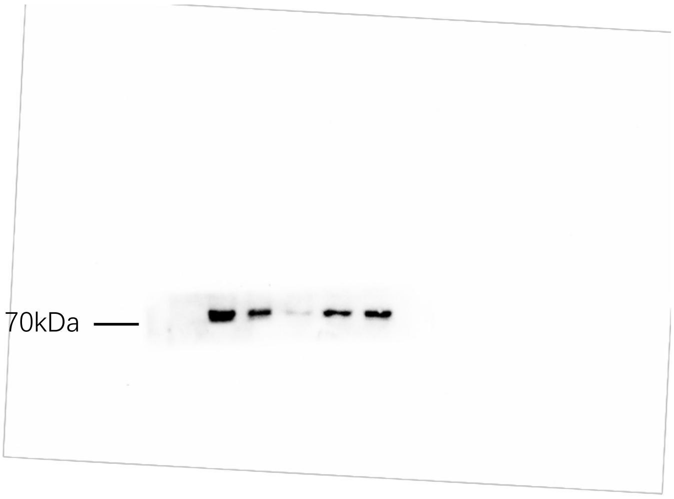

FIGURE6F-IB-ATF6(75kDa)

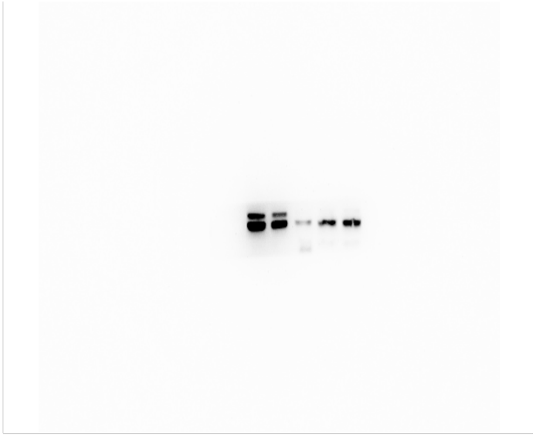

FIGURE6F-IB-GRP78

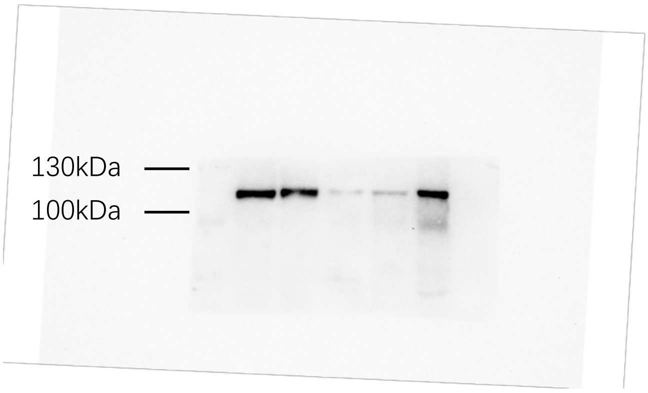

FIGURE6F-IB-IRE1(110kDa)

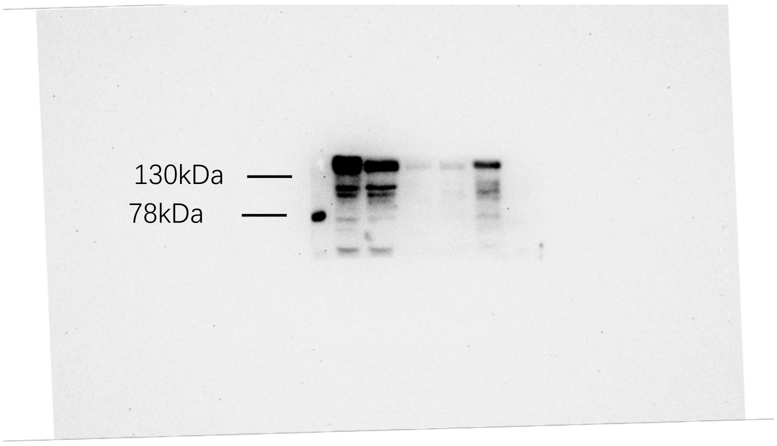

FIGURE6F-IB-PERK

(140kDa)

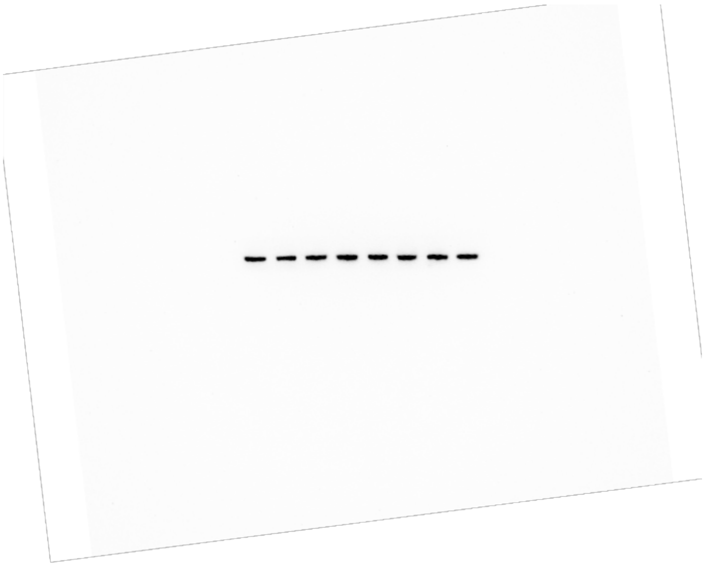

FIGURE6G-ATF6

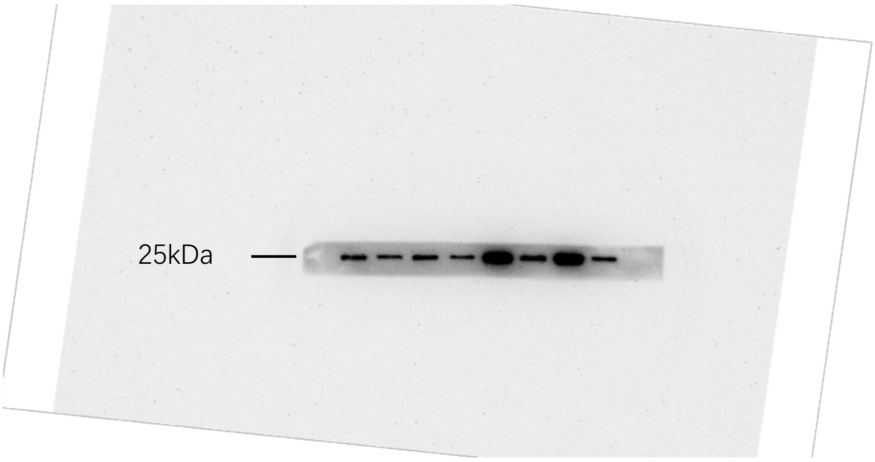

FIGURE6G-CHOP

(19kDa)

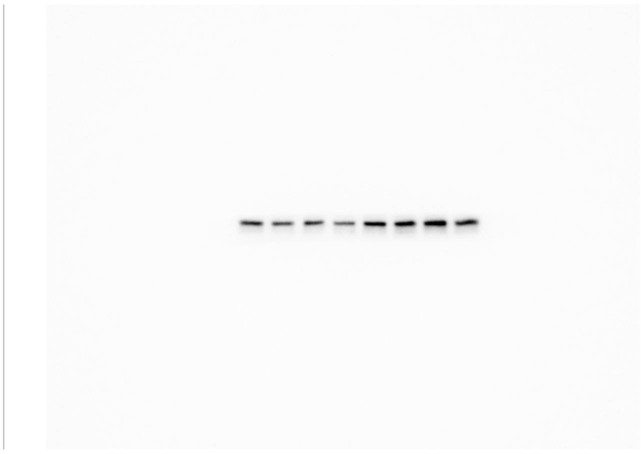

FIGURE6G-EIF2

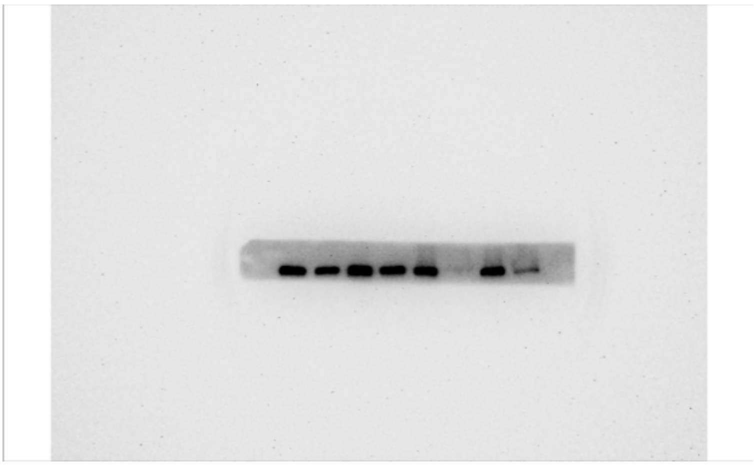

FIGURE6G-IRE1

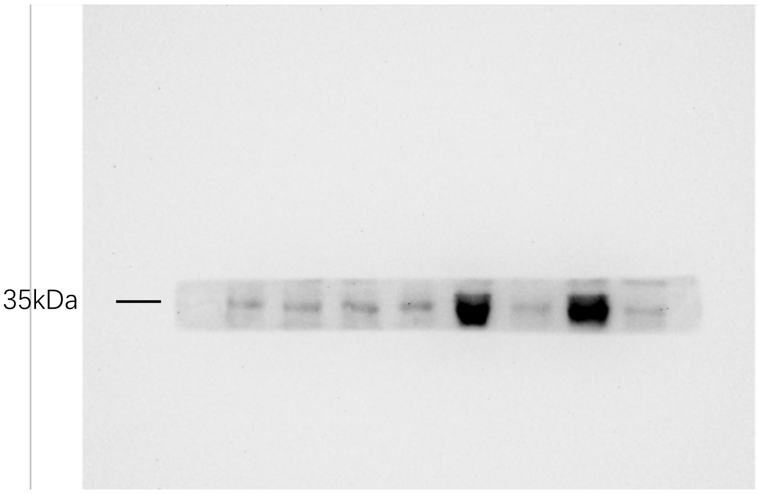

FIGURE6G-p-EIF2

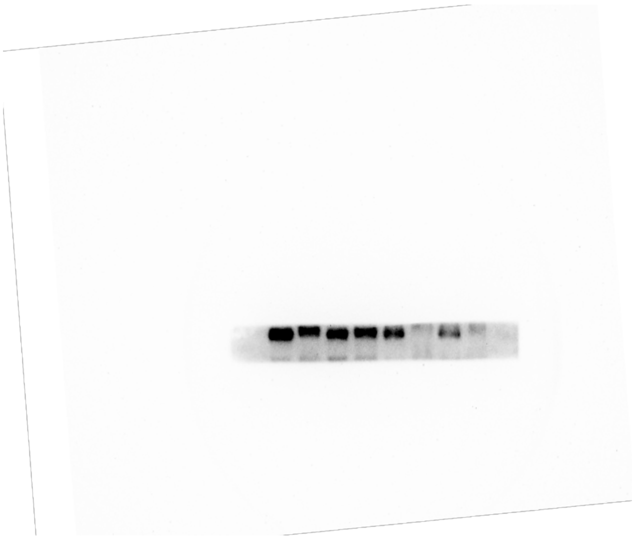

FIGURE6G-PERK

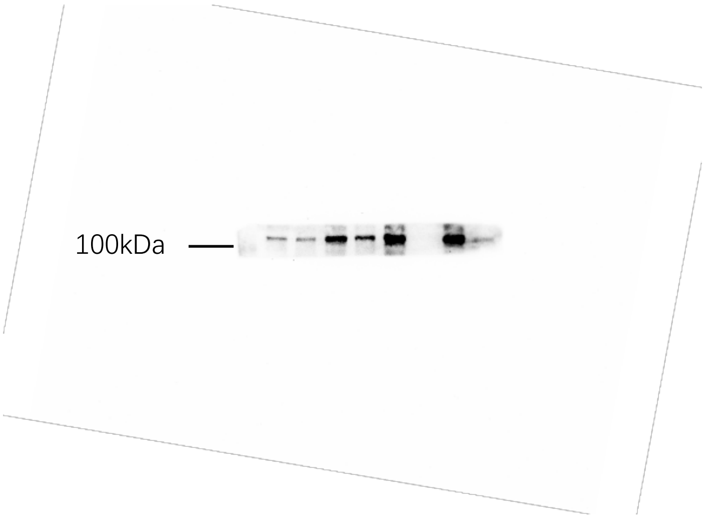

FIGURE6G-p-IRE1（110kDa）

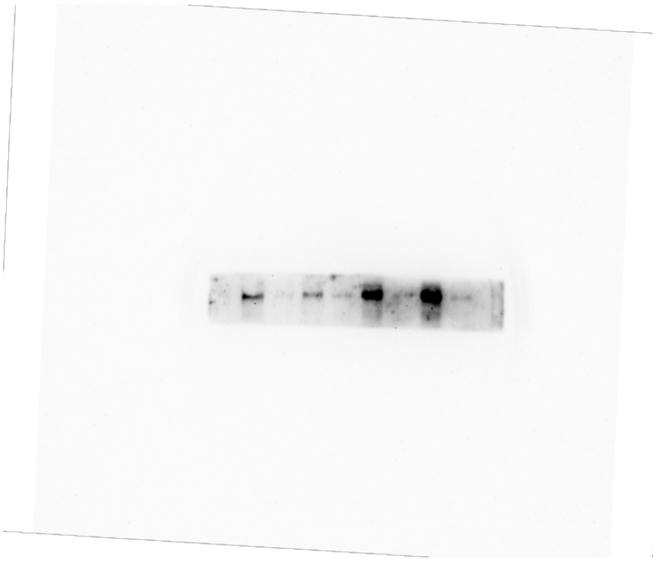

FIGURE6G-p-PERK

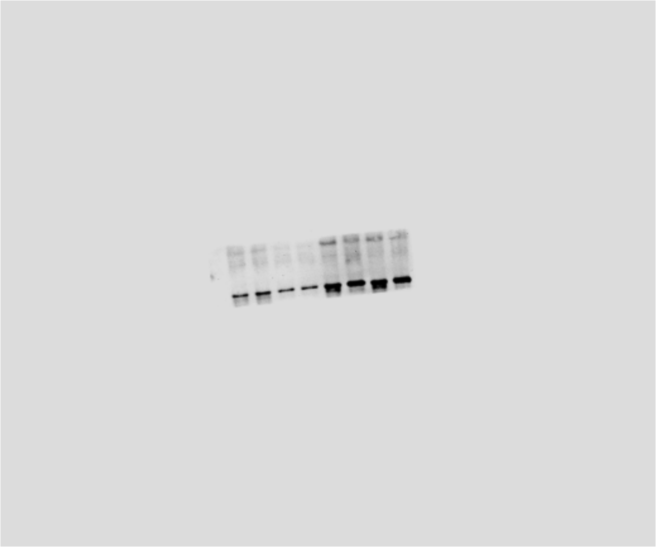

FIGURE6G-XBP1

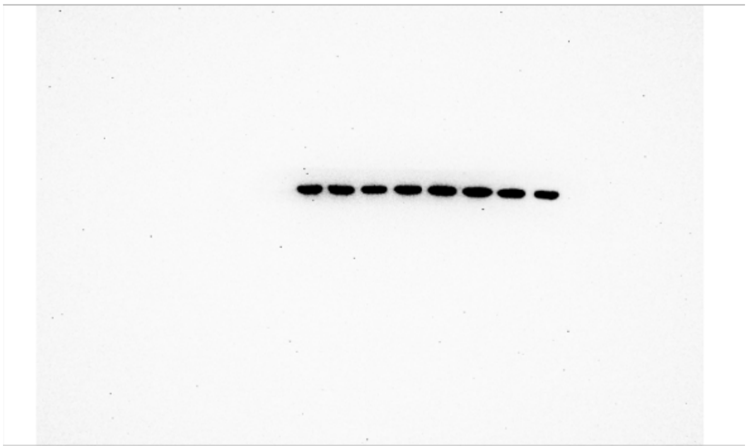

FIGURE6G-β-actin

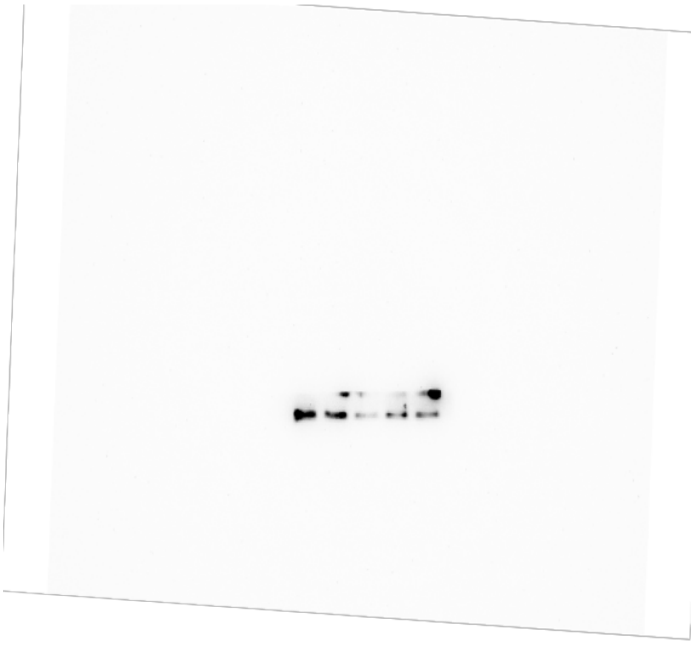

FIGURE.S4-ART1

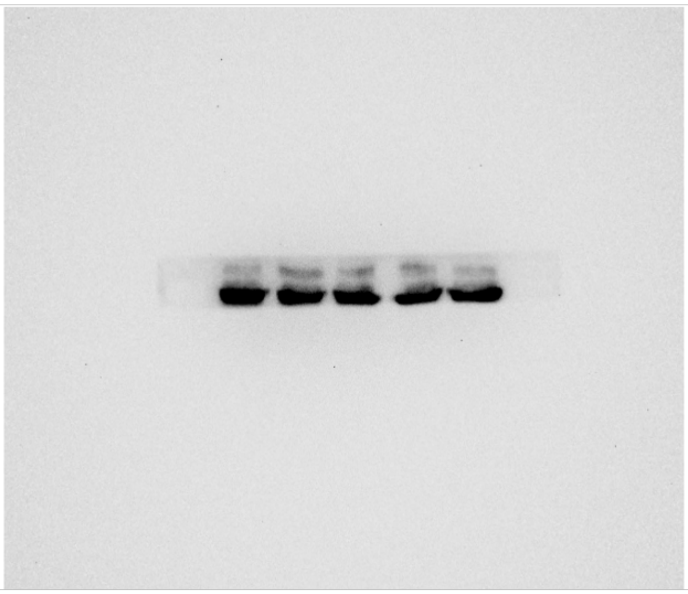

FIGURE.S4-ATF6

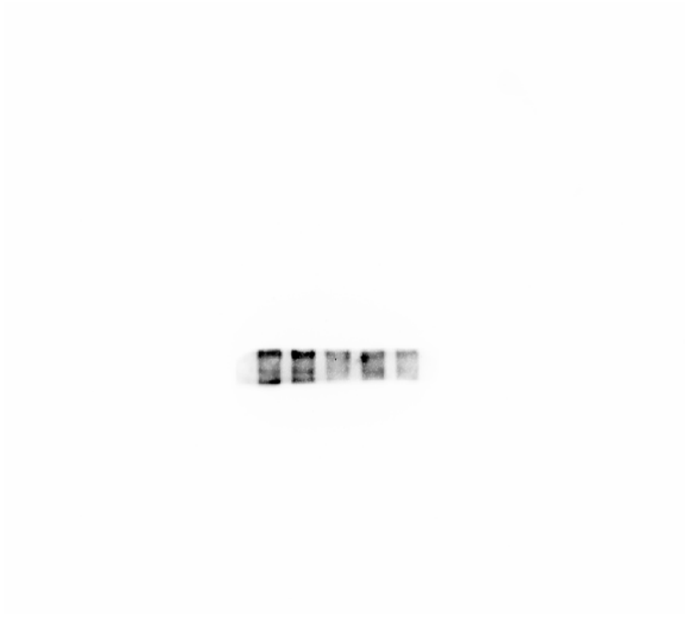

FIGURE.S4-CHOP

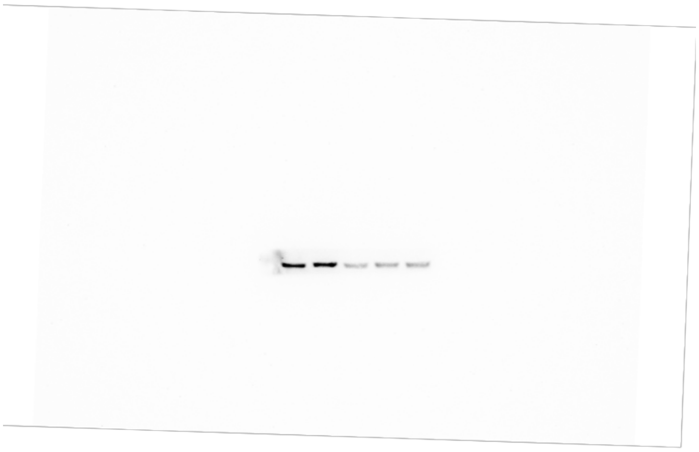

FIGURE.S4-GRP78

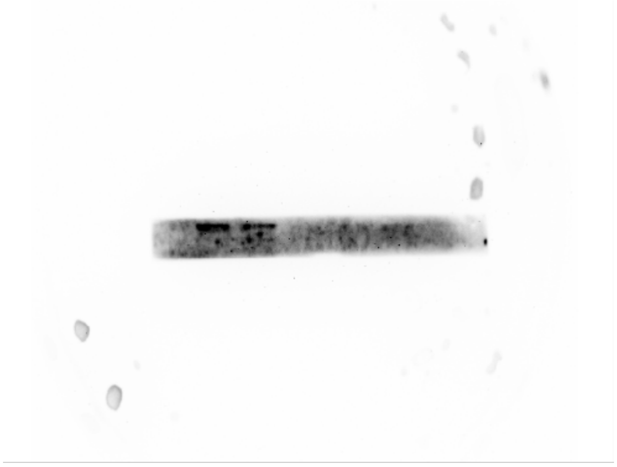

FIGURE.S4-p-EIF2

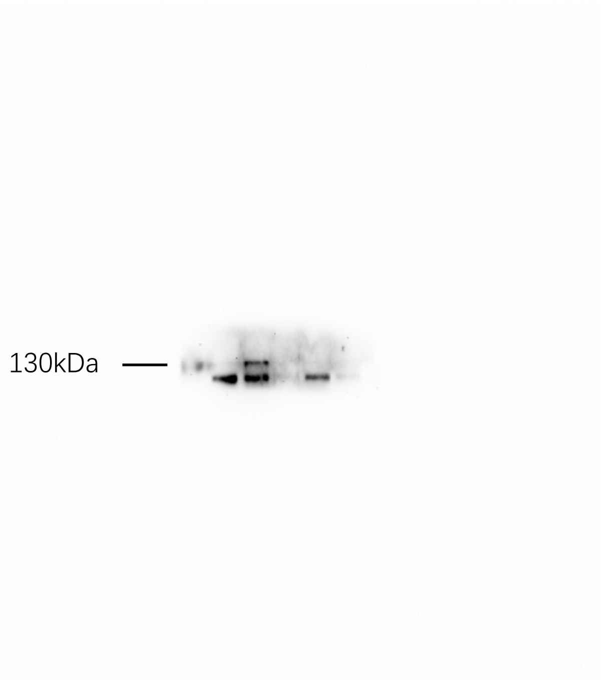

FIGURE.S4-p-IRE1（110kDa）

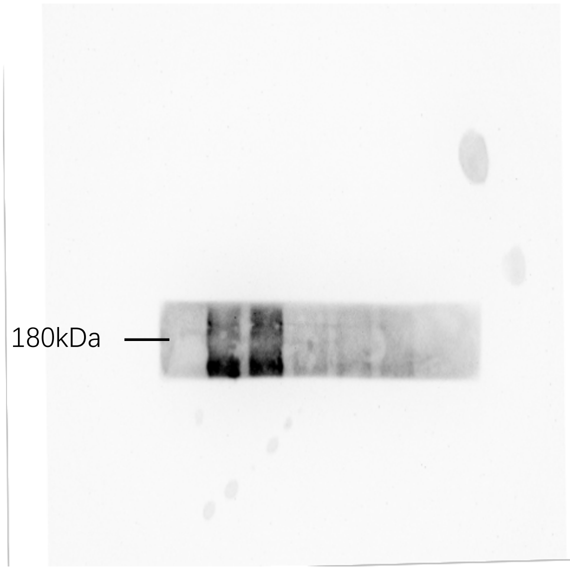

FIGURE.S4-p-PERK（140kDa）

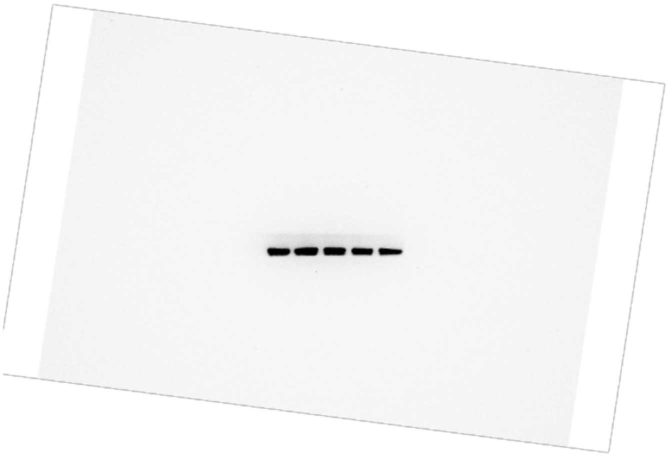

FIGURE.S4-β-actin

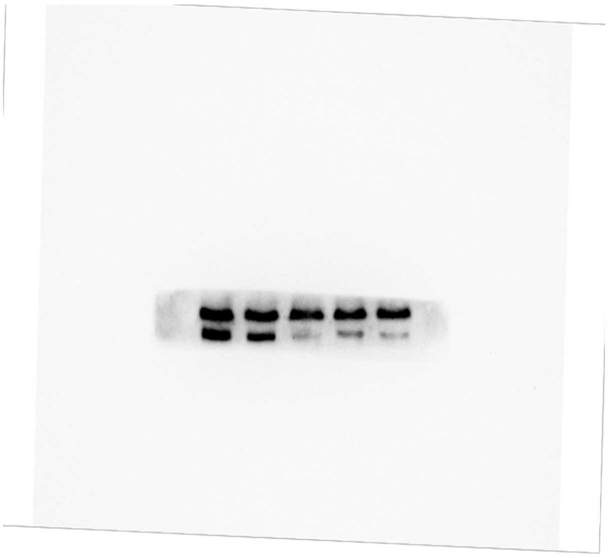
FIGURE.S5-ART1

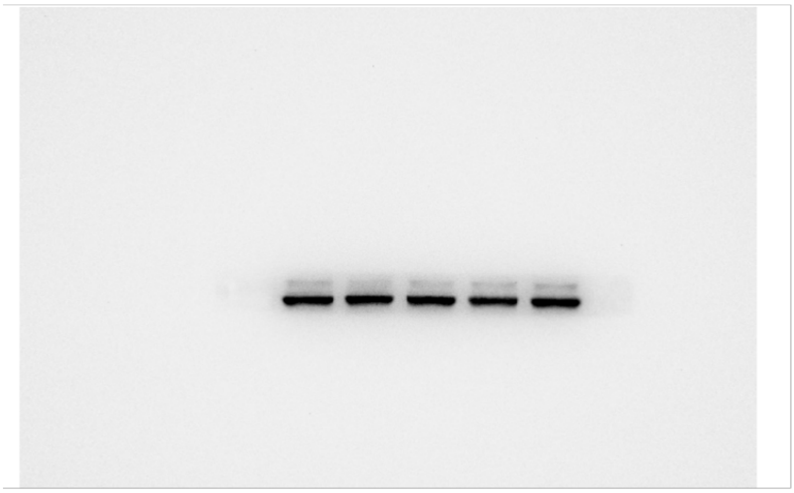
FIGURE.S5-ATF6

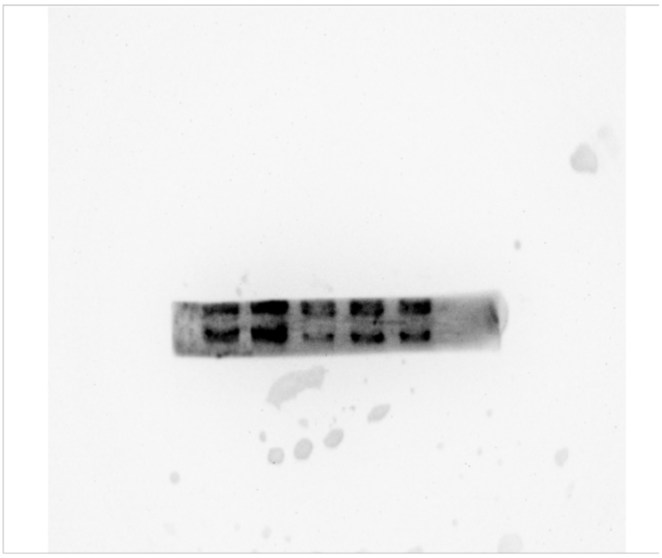
FIGURE.S5-CHOP

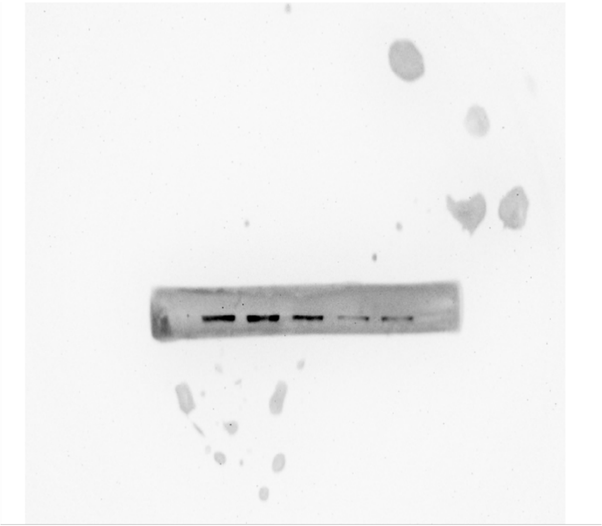

FIGURE.S5-GRP78

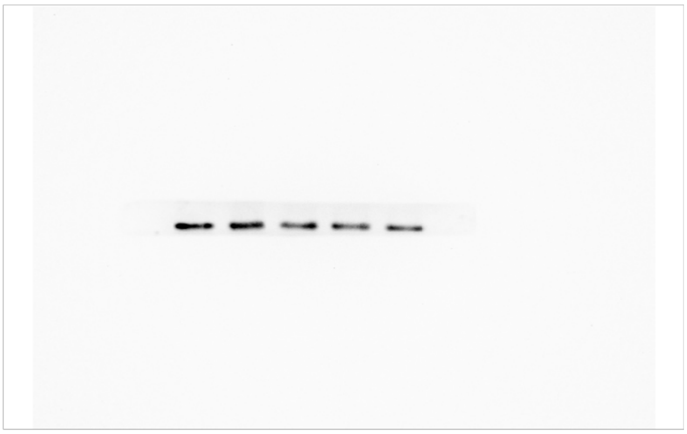

FIGURE.S5-p-EIF2

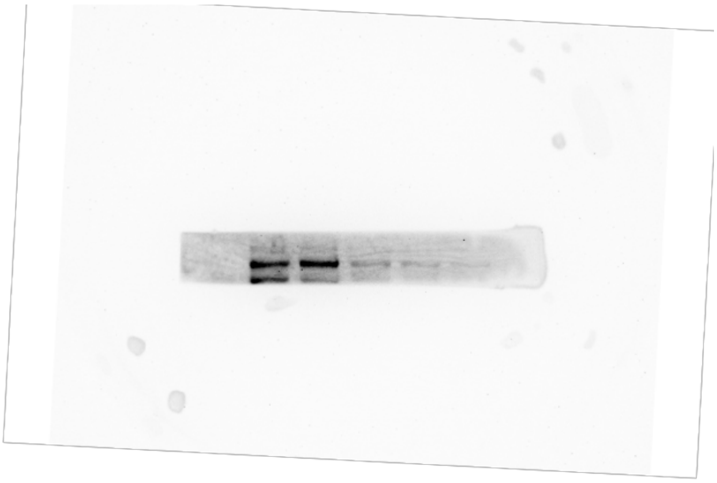

FIGURE.S5-p-IRE1

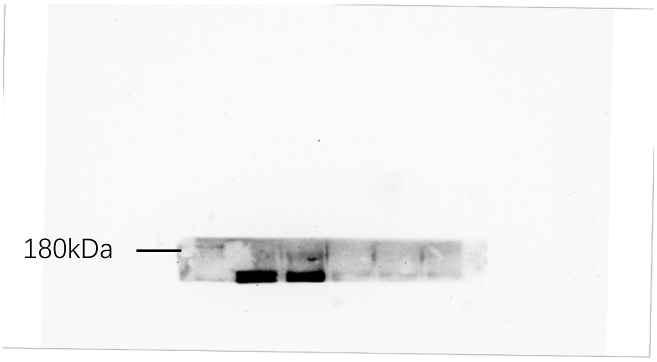

FIGURE.S5-p-PERK（170kDa）

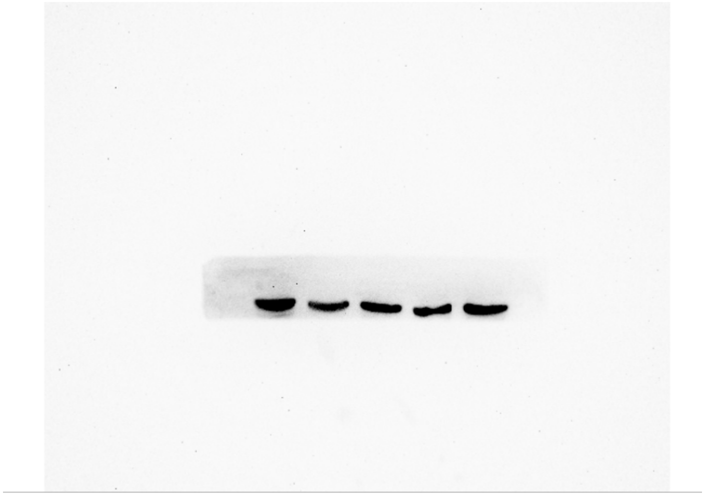
FIGURE.S5-β-actin

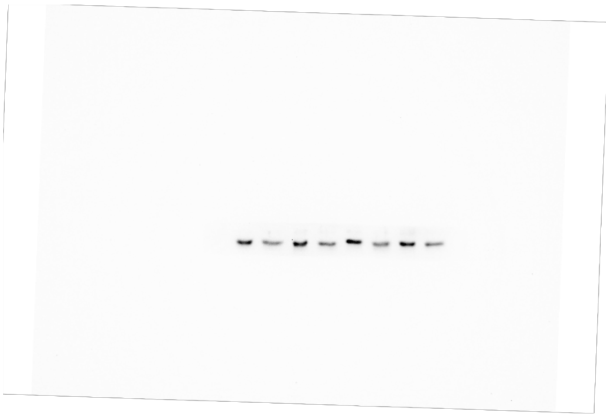
FIGURE6I-ART1

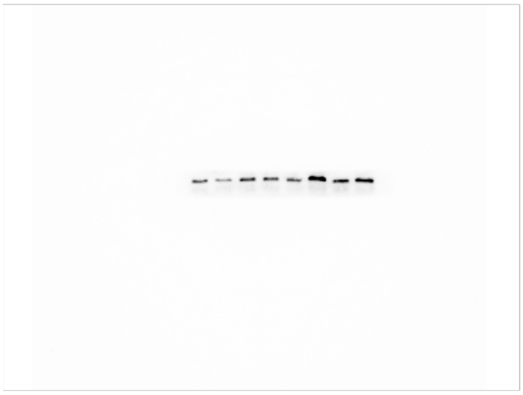
FIGURE6I-Cleaved-caspase3

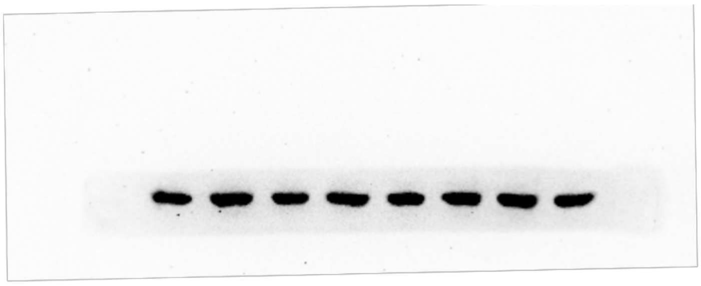

FIGURE6I-β-actin

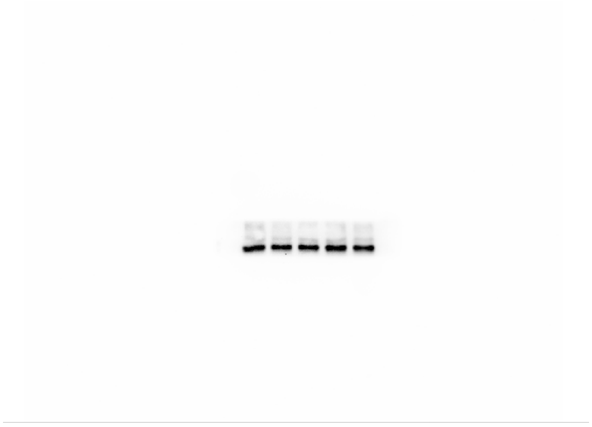

FIGURE7C-ATF6

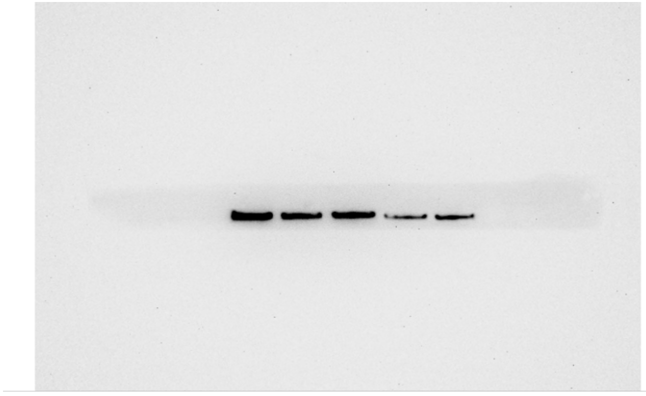

FIGURE7C-P-EIF2

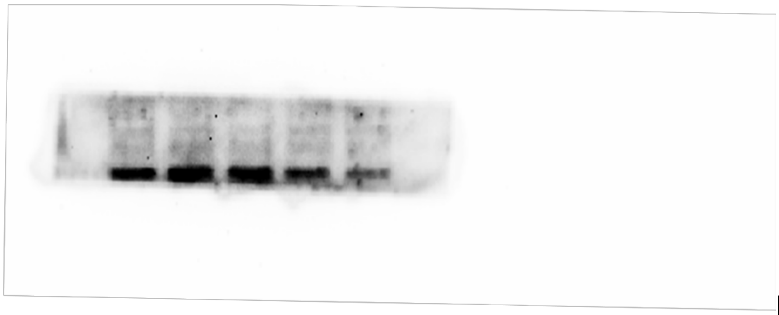

FIGURE7C-P-IRE1

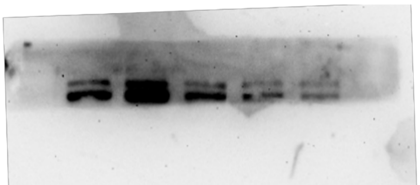

FIGURE7C-P-PERK

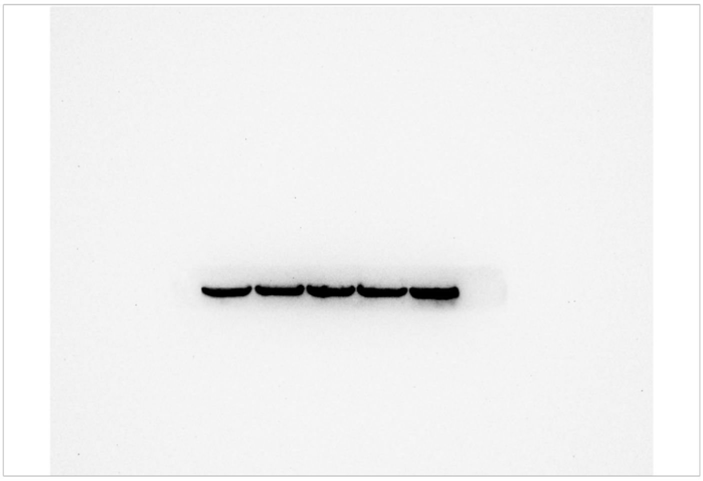

FIGURE7C-β-actin
